# Design, Synthesis, and Evaluation of Novel 2-Methoxyestradiol Derivatives as Apoptotic Inducers through an Intrinsic Apoptosis Pathway

**DOI:** 10.3390/biom10010123

**Published:** 2020-01-10

**Authors:** Li-Xin Sheng, Jiang-Yu Zhang, Li Li, Xiao Xie, Xiao-An Wen, Ke-Guang Cheng

**Affiliations:** 1 State Key Laboratory for the Chemistry and Molecular Engineering of Medicinal Resources, School of Chemistry and Pharmacy of Guangxi Normal University, Guilin 541004, China; 18874339441@163.com (L.-X.S.); 17864262597@163.com (J.-Y.Z.); lili770215@aliyun.com (L.L.); 2 Unité 1177 (Drugs and Molecules for Living Systems)—Inserm, Université Lille 2, Institut Pasteur de Lille, F-59000 Lille, France; 3 Jiangsu Key Laboratory of Drug Discovery for Metabolic Diseases and State Key Laboratory of Natural Medicines, Center of Drug Discovery, China Pharmaceutical University, 24 Tongjia Xiang, Nanjing 210009, China; wxagj@cpu.edu.cn

**Keywords:** 2-methoxyestradiol, anti-tumor activity, intrinsic apoptosis pathway

## Abstract

In order to discover novel derivatives in the anti-tumor field, reported anti-tumor pharmacophores (uridine, uracil, and thymine) were combined with 2-methoxyestradiol, which has been characterized as having excellent biological properties in terms of anti-tumor activity. Thus, 20 hybrids were synthesized through etherification at the 17*β*-OH or 3-phenolic hydroxyl group of 2-methoxyestradiol, and evaluated for their biological activities against the human breast adenocarcinoma MCF-7 cell lines, human breast cancer MDA-MB-231 cell lines, and the normal human liver L-O2 cell lines. As a result, all the uridine derivatives and single-access derivatives of uracil/thymine possessed good anti-proliferative activity against tested tumor cells (half maximal inhibitory concentration values from 3.89 to 19.32 µM), while only one dual-access derivative (**21b**) of thymine possessed good anti-proliferative activity (half maximal inhibitory concentration ≈ 25 µM). Among them, the uridine derivative **11** and the single-access derivative of uracil **12a** possessed good anti-proliferative selectivity against tested tumor cells. Furthermore, basic mechanism studies revealed that hybrids **11** and **12a** could induce apoptosis in MCF-7 cells through mitochondrial pathway. These hybrids induced morphological changes in MCF-7 cells, causing mitochondrial depolarization. These two hybrids also had the following effects: arrest of the cell cycle at the G2 phase; up regulation of Apaf-1, Bax, and cytochrome c; down regulation of Bcl-2 and Bcl-xL for both mRNA and protein; and increase of the expression for caspase-8 and -9. Finally, apoptotic effector caspase-3 was increased, which eventually caused nuclear apoptosis at least through an intrinsic pathway in the mitochondria. Additionally, hybrids **11** and **12a** could specifically bind to estradiol receptor alpha in a dose-dependent manner.

## 1. Introduction

**Figure 1:**
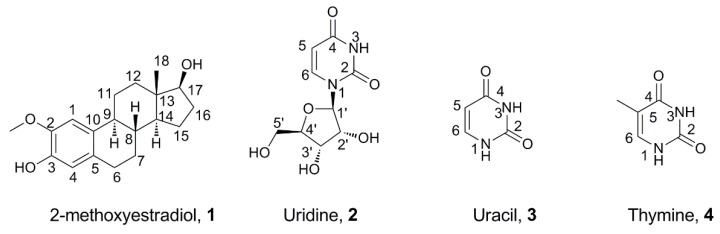
Chemical structures of 2-methoxyestradiol, uridine, uracil, and thymine.

2-Methoxyestradiol (**1**, Figure 1), which is the methylation metabolite of 2-hydroxyetradiol under the action of catechol-*O*-methyltransferase (COMT), has been elucidated as a highly potent anti-proliferative metabolite against different tumor cells and can induce apoptosis both in vitro and in vivo [1,2,3,4]. It is a well-tolerated small molecule and has been used in several clinical trial development programs [5,6,7]. Furthermore, 2-methoxyestradiol has attracted considerable interest in terms of its biological activities, due to its anti-proliferation efficacy with lower toxicity [1,8]. Most biological studies have found that 2-methoxyestradiol possesses many marked anticarcinogenic properties without any undesirable estrogen activities [9,10] and is associated with the disruption of cellular events, such as affecting cell cycle events including arresting mitotic cells during metaphase by disrupting correct microtubule assembly, and inducing cellular alterations of apoptosis [9,11]. For example, 2-methoxyestradiol could induce apoptosis and arrest in the G2 phase of the cell cycle both against nasopharyngeal carcinoma [12] and human prostate cancer cell lines [13], inhibit the expression of caspase-3 [14], protect the brain from ischemic injury [15], and regulate the protein level of Bax or Bcl-2 [16].

Based on the published studies, 2-methoxyestradiol could inhibit the growth of different tumors cell lines [17,18,19], and the main model is breast cancer [20,21,22,23,24]. Furthermore, it has been indicated that 2-methoxyestradiol is selective to some specific cancer cell lines [9,11]. Furthermore, it has also been found that combination treatment with 2-methoxyestradiol could lead to a significant growth inhibition, which could not have been achieved with the individual components, especially with regards to additive or synergistic inhibition of cell proliferation [11,25]. Thus, there are many structure–activity relationship studies conducted with 2-methoxyestradiol as a lead compound, which consist of many estrogen-like molecules derived from various sources [20,26,27,28].

In recent years, uridine, uracil, and thymine (**2**, **3**, and **4**, Figure 1) have attracted much interest due to their promising potential biological activities, and their modified derivatives have exhibited excellent activities, such as antibacterial, inhibition against 1,4-*β*-galactosyltransferase, antivirus, anticancer, and so on [29,30,31,32,33,34,35,36]. Our previous studies [37,38] have also shown that uridine/uracil/thymine attached to the natural compound oleanolic acid could afford derivatives that possess high anti-proliferative activities against tumor cell lines and exhibit clearly inducing effects on cell apoptosis.

**Scheme 1:**
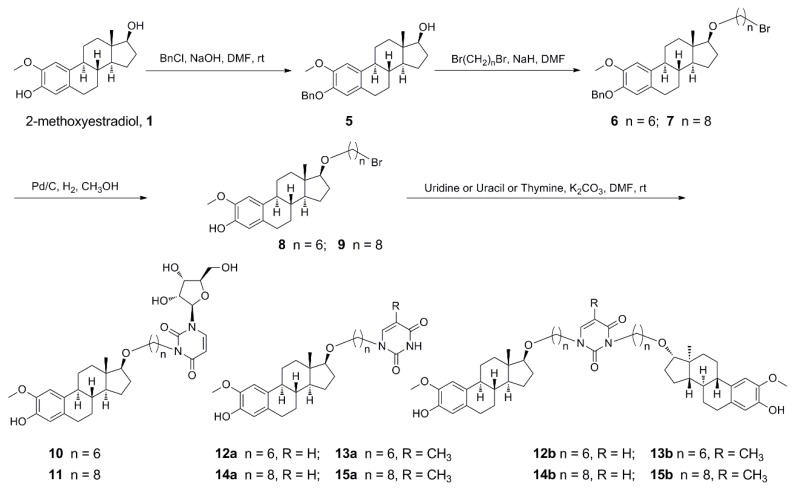
Synthetic pathway for derivatives of etherification of 2-methoxyestradiol at 17*β*-OH.

**Scheme 2:**
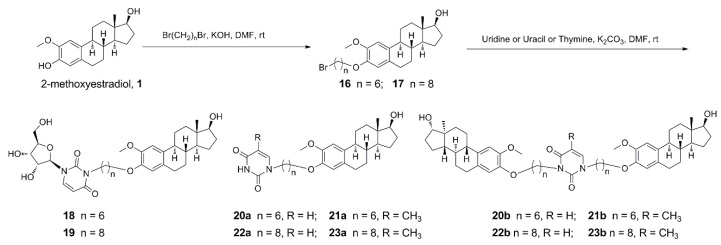
Synthetic pathway for derivatives of etherification of 2-methoxyestradiol at 3-phenolic hydroxyl.

Based on the above information, to the best of our knowledge, the hybridization of uridine/uracil/thymine and 2-methoxyestradiol is reasonable in the quantitative structure–activity relationship (QSAR) study and has potential activities in anticancer therapy. Therefore, in this study, uridine/uracil/thymine was linked to 2-methoxyestradiol through varying lengths of carbon chains to afford several targeted 2-methoxyestradiol-uridine/uracil/thymine conjugates (Scheme 1 and Scheme 2). Their anti-tumor activities were evaluated to explore the structure–activity relationships of a series of derivatives from 2-methoxyestradiol.

## 2. Materials and Methods

### 2.1. Chemicals and Biology

All the chemical reagents and solvents used were of analytical grade and used without further purification unless specified. All commercial reagents were purchased from Aladdin (Shanghai, China) Industrial Corporation. Melting points were measured on a RY-1 melting point apparatus (TianJin, China). Proton nuclear magnetic resonance (^1^H NMR) and Carbon nuclear magnetic resonance (^13^C NMR) spectra were recorded on a Bruker AV-500 (500/125 MHz for ^1^H/^13^C) spectrometer (Fällanden, Switzerland). Chemical shifts were reported as values from an internal tetramethylsilane standard. High-resolution mass spectra (HRMS) measurements were recorded on a Thermo Scientific Accela Exactive high-resolution accurate mass spectrometer (Waltham, MA, USA) or an Agilent high-resolution liquid mass spectrometer (Santa Clara, CA, USA). All the tests of biological activities were conducted at Nanjing KeyGen Biotech Co. Ltd. (Nanjing, China).

### 2.2. General Procedure for the Synthesis of 2-Methoxy-3-Benzyloxy-Estra-1,3,5 (10)-Triene-17β-ol (***5***)

A solution of 2-methoxyestradiol (0.200 g, 0.661 mmol) and NaOH (0.053 g, 1.322 mmol) in dry DMF (3 mL) was stirred at room temperature for 30 min, and benzyl chloride (0.15 mL, 1.322 mmol) was added. After stirring at room temperature for 24 h, the mixture was diluted with H_2_O (20 mL) and extracted with EtOAc (3 × 15 mL). The combined organic layers were washed successively with 1 N HCl, saturated aqueous NaHCO_3_ and brine, dried (Na_2_SO_4_), filtered, and concentrated. The residue was purified by flash column chromatography [39]. Yield: 0.213 g, 82%, white solid, melting point (mp) 84–85 °C. R_f_ = 0.68 (Petroleum ether: EtOAc = 1:4). ^1^H NMR (500 MHz, CDCl_3_) δ 0.79 (s, 3 H, CH_3_), 1.16–2.33 (m, 13 H), 2.75 (m, 2 H, H-6), 3.73 (t, 1 H, *J* = 8.5 Hz, H-17), 3.86 (s, 3 H, OCH_3_), 5.10 (s, 2 H, Ph-CH_2_-O), 6.63 and 6.86 (2 s, each 1 H, H-4, H-1), 7.29 (t, 1 H, *J* = 7.3 Hz, Ph-4), 7.36 (t, 2 H, *J* = 7.4 Hz, Ph-3, Ph-5), 7.45 (d, 2 H, *J* = 7.5 Hz, Ph-2, Ph-6). HRMS (ESI) m/z: [M + H]^+^ calcd for C_26_H_33_O_3_, 393.2430; found 393.2429 (see Supplementary Materials).

### 2.3. General Procedure I for Etherification at 17β-OH from Compound ***5***

To a solution of compound **5** (0.201 g, 0.511 mmol) in dry DMF (3 mL) stirred in an ice-water bath, NaH (60%, 0.210 g, 5.11 mmol) was slowly added, and the reaction was continuing with stirring in the ice-water bath for 1 h. Then, α, ω-dibromoalkane (5.11 mmol) was added, and the ice-water bath was removed. After stirring at room temperature for 24 h, the mixture was diluted with H_2_O (20 mL) and extracted with EtOAc (3 × 15 mL). The combined organic layers were washed successively with 1 N HCl, saturated aqueous NaHCO_3_ and brine, dried (Na_2_SO_4_), filtered, and concentrated. The residue was purified by flash column chromatography.

#### 2.3.1. 17 β-(6-Bromohexyloxy)-2-Methoxy-3-Benzyloxy-Estra-1,3,5(10)-Triene (**6**)

Prepared from compound **5** (0.200 g, 0.511 mmol) and 1,6-dibromohexane (0.78 mL, 5.11 mmol) according to general procedure I. The residue was purified by flash column chromatography. Yield: 0.260 g, 92%, white solid, mp 114–115 °C. R_f_ = 0.8 (Petroleum ether: EtOAc = 3:1). ^1^H NMR (500 MHz, CDCl_3_) δ 0.80 (s, 3 H, CH_3_), 1.37–2.28 (m, 21 H), 2.75 (m, 2 H, H-6), 3.36–3.52 (m, overlapping, 5 H, OCH_2_, CH_2_Br, H-17), 3.87 (s, 3 H, OCH_3_), 5.11 (s, 2 H, Ph-CH_2_-O), 6.63 and 6.85 (2 s, each 1 H, H-4, H-1), 7.30 (t, 1 H, *J* = 7.3 Hz, Ph-4), 7.37 (t, 2 H, *J* = 7.5 Hz, Ph-3, Ph-5), 7.45 (d, 2 H, *J* = 7.4 Hz, Ph-2, Ph-6). HRMS (ESI) m/z: [M + H]^+^ calcd for C_32_H_44_BrO_3_, 555.2474; found 555.2287.

#### 2.3.2. 17 β-(8-Bromooctyloxy)-2-Methoxy-3-Benzyloxy-Estra-1,3,5(10)-Triene (**7**)

Prepared from compound **5** (0.268 g, 0.683 mmol) and 1,8-dibromooctane (1.26 mL, 6.83 mmol) according to general procedure I. The residue was purified by flash column chromatography. Yield: 0.350 g, 88%, white solid, mp 121–122 °C. R_f_ = 0.75 (Petroleum ether: EtOAc = 3:1). ^1^H NMR (500 MHz, CDCl_3_) δ 0.79 (s, 3 H, CH_3_), 1.32–2.42 (m, 25 H), 2.75 (m, 2 H, H-6), 3.39–3.41 (m, overlapping, 5 H, OCH_2_, CH_2_Br, H-17), 3.86 (s, 3 H, OCH_3_), 5.10 (s, 2 H, Ph-CH_2_-O), 6.62, 6.84 (2 s, each 1 H, H-4, H-1), 7.29 (t, 1 H, *J* = 7.3 Hz, Ph-4), 7.36 (t, 2 H, *J* = 7.5 Hz, Ph-3, Ph-5), 7.44 (d, 2 H, *J* = 7.5 Hz, Ph-2, Ph-6). ^13^C NMR (125 MHz, CDCl_3_) δ 11.7, 15.3, 23.1, 26.2, 28.1, 28.7, 29.3, 29.8, 30.2, 33.4, 34.0, 38.2, 38.6, 43.4, 44.3, 50.3, 56.4, 61.9, 66.1, 70.2, 70.9, 71.2, 74.1, 89.1, 109.9, 114.7, 127.3, 127.7, 128.5, 128.9, 137.5, 146.3, 147.6. HRMS (ESI) m/z: [M + Na]^+^ calcd for C_34_H_47_BrNaO_3_, 605.2606; found 605.2609.

### 2.4. General Procedure II for Deprotection of the Benzyl Group

To a solution of compound **6**/**7** (0.900 mmol) in dry CH_3_OH (5 mL), Pd/C (10%, 0.050 g) was added. After stirring in an atmosphere of H_2_ at room temperature for 2.5 h, the mixture was filtered and concentrated. The residue was purified by flash column chromatography.

#### 2.4.1. 17 β-(6-Bromohexyloxy)-2-Methoxy-Estra-1,3,5(10)-Triene-3-ol (**8**)

Prepared from compound **6** (0.500 g, 0.900 mmol) according to general procedure II. The residue was purified by flash column chromatography. Yield: 0.37 g, 88%, white solid, mp 101–103 °C. R_f_ = 0.26 (Petroleum ether: EtOAc = 10: 1). ^1^H NMR (500 MHz, CDCl_3_) δ 0.81 (s, 3 H, CH_3_), 1.26–2.25 (m, 21 H), 2.77 (m, 2 H, H-6), 3.36–3.56 (m, overlapping, 5 H, OCH_2_, CH_2_Br, H-17), 3.87 (s, 3 H, OCH_3_), 5.43 (s, 1 H, OH), 6.65 and 6.80 (2 s, each 1 H, H-4, H-1). ^13^C NMR (125 MHz, CDCl_3_) δ 11.7, 23.1, 25.5, 26.8, 27.3, 28.0, 28.2, 29.0, 30.0, 32.6, 32.8, 33.8, 33.9, 38.1, 38.6, 43.4, 44.3, 50.3, 56.1, 69.9, 89.1, 100.1, 108.2, 114.6, 129.6, 131.9, 143.5, 144.6. HRMS (ESI) m/z: [M + Na]^+^ calcd for C_25_H_37_Br NaO_3_, 487.1824; found 487.1803.

#### 2.4.2. 17 β-(8-Bromooctyloxy)-2-Methoxy-Estra-1,3,5(10)-Triene-3-ol (**9**)

Prepared from compound **7** (0.500 g, 0.857 mmol) according to general procedure II. The residue was purified by flash column chromatography. Yield: 0.38 g, 90%, mp 107–109 °C. R_f_ = 0.38 (Petroleum ether: EtOAc = 10: 1). ^1^H NMR (500 MHz, CDCl_3_) δ 0.79 (s, 3 H, CH_3_), 1.25–2.26 (m, 25 H), 2.77 (m, 2 H, H-6), 3.35–3.49 (m, overlapping, 5 H, OCH_2_, CH_2_Br, H-17), 3.86 (s, 3 H, OCH_3_), 5.43 (s, 1 H, OH), 6.64 and 6.79 (2 s, each 1 H, H-4 and H-1). ^13^C NMR (125 MHz, CDCl_3_) δ 11.7, 23.1, 26.2, 26.8, 27.3, 28.1, 28.2, 28.7, 29.0, 29.3, 30.2, 32.8, 34.0, 38.1, 38.6, 43.3, 44.3, 50.3, 56.1, 70.2, 89.1, 108.1, 109.8, 114.6, 129.6, 131.9, 143.4, 144.6. HRMS (ESI) m/z: [M − H]^+^ calcd for C_27_H_40_BrO_3_, 491.2167; found 491.2175.

### 2.5. General Procedure III for N-Alkylation of Uridine/Uracil/Thymine

To a solution of compound **8**/**9** (0.730 mmol) in DMF (8 mL), K_2_CO_3_ (2.19 mmol) and uridine/uracil/thymine (2.19 mmol) were added. After stirring at 50 °C for 6 h, the mixture was diluted with H_2_O (30 mL) and extracted with EtOAc (3 × 20 mL). The combined organic layers were washed successively with 1 N HCl, saturated aqueous NaHCO_3_ and brine, dried (Na_2_SO_4_), filtered, and concentrated. The residue was purified by flash column chromatography.

#### 2.5.1. 17 β-[6-(1-β-d-Ribofuranosyluracil-3-yl)-Hexyloxy]-2-Methoxy-Estra-1,3,5(10)-Triene-3-ol (**10**)

Prepared from compound **8** (0.340 g, 0.730 mmol) and uridine (0.535 g, 2.19 mmol) according to general procedure III. The residue was purified by flash column chromatography. Yield: 0.320 g, 70%, white solid, mp 79–81 °C. R_f_ = 0.59 (CH_2_Cl_2_:CH_3_OH = 6:1). ^1^H NMR (500 MHz, CDCl_3_) δ 0.77 (s, 3 H, CH_3_), 1.15–2.24 (m, 21 H), 2.75 (m, 2 H, H-6), 3.43 (m, overlapping, 5 H, OCH_2_, H-5′′and H-17), 3.85 (s, 3 H, OCH_3_), 3.89 (m, 2 H_,_ NCH_2_), 3.96 (dd, 1 H, *J* = 12.9 and 3.2 Hz, H-2′′), 4.17 (m, 1 H, H-4′′), 4.31 (m, 1 H, H-3′′), 5.54 (s, 1 H, OH), 5.69 (d, 1 H, *J* = 4.1 Hz, H-1′′), 5.76 (d, 1 H, *J* = 8.1 Hz, H-5*^Uri^*), 6.62 and 6.78 (2 s, each 1 H, H-4, H-1), 7.67 (d, 1 H, *J* = 8.1 Hz, H-6*^Uri^*). ^13^C NMR (125 MHz, CDCl_3_) δ 11.7, 13.7, 19.1, 23.0, 25.9, 26.7, 28.19, 29.0, 30.5, 38.1, 38.6, 43.3, 44.2, 50.3, 56.1, 62.1, 65.5, 70.1, 71.0, 75.1, 85.9, 89.0, 93.7, 101.9, 108.1, 114.6, 128.8, 130.9, 138.6, 143.4, 144.5. HRMS (ESI) m/z: [M + Cl]^−^ calcd for C_34_H_48_ClN_2_O_9_, 663.3048; found 663.3060.

#### 2.5.2. 17 β-[8-(1-β-d-Ribofuranosyluracil-3-yl)-Octyloxy]-2-Methoxy-Estra-1,3,5(10)-Triene-3-ol (**11**)

Prepared from compound **9** (0.262 g, 0.530 mmol) and uridine (0.389 g, 1.59 mmol) according to general procedure III. The residue was purified by flash column chromatography. Yield: 0.266 g, 76%, white solid, mp 95–96 °C. R_f_ = 0.60 (CH_2_Cl_2_:CH_3_OH = 6:1). ^1^H NMR (500 MHz, CDCl_3_) δ 0.78 (s, 3 H, CH_3_), 1.17–2.23 (m, 25 H), 2.74 (m, 2 H, H-6), 3.37–3.47 (m, overlapping, 5 H, OCH_2_, H-5′′and H-17), 3.84 (s, 3 H, OCH_3_), 3.94–4.34 (m, 5 H, NCH_2_, H-2′′, H-3′′, H-4′′), 5.59 (s, 1 H, OH), 5.69 (s, 1 H, H-1′′), 5.74 (d, 1 H, *J* = 8.1 Hz, H-5*^Uri^*), 6.62 and 6.78 (2 s, each 1 H, H-4, H-1), 7.68 (d, 1 H, *J* = 8.1 Hz, H-6*^Uri^*). ^13^C NMR (125 MHz, CDCl_3_) δ 11.6, 15.2, 23.0, 26.1, 26.8, 27.4, 28.1, 29.30, 29.6, 30.1, 38.1, 38.6, 41.2, 43.3, 44.2, 50.2, 56.0, 62.0, 66.1, 70.8, 85.7, 89.0, 93.6, 101.9, 108.1, 114.6, 129.5, 131.8, 138.7, 144.5, 151.7, 162.6. HRMS (ESI) m/z: [M + H]^+^ calcd for C_36_H_53_N_2_O_9_, 657.3751; found 657.3742.

#### 2.5.3. 17 β-[6-(Uracil-3-yl)-Hexyloxy]-2-Methoxy-Estra-1,3,5(10)-Triene-3-ol (**12a**) and 1,3-bis-{6-[2-Methoxy-3-Hydroxy-Estra-1,3,5(10)-Triene-17β-yl-oxy]-Hexyl}-Uracil (**12b**)

Prepared from compound **8** (0.419 g, 0.918 mmol) and uracil (0.302 g, 2.75 mmol) according to general procedure III. The residue was purified by flash column chromatography to afford **12a** (white solid, 0.342 g, 77%) and **12b** (white solid, 0.126 g, 16%).

Compound **12a**: mp 76–78 °C. R_f_ = 0.37 (Petroleum ether: EtOAc = 1:4). ^1^H NMR (500 MHz, CDCl_3_) δ 0.78 (s, 3 H, CH_3_), 1.15–2.35 (m, 21 H), 2.75 (m, 2 H, H-6), 3.35 (t, 1 H, *J* = 8.3 Hz, H-17), 3.45 (m, 2 H, OCH_2_), 3.72 (t, 2 H, *J* = 7.2 Hz, NCH_2_), 3.85 (s, 3 H, OCH_3_), 5.70 (d, 1 H, *J* = 7.8 Hz, H-5*^Ura^*), 6.63, 6.78 (2 s, each 1 H, H-4, H-1), 7.15 (d, 1 H, *J* = 7.8 Hz, H-6*^Ura^*), 8.88 (s, 1 H, NH). ^13^C NMR (125 MHz, CDCl_3_) δ 11.7, 23.0, 25.9, 26.3, 26.7, 27.3, 28.2, 29.0, 29.9, 38.1, 38.6, 43.3, 44.2, 48.8, 50.2, 56.0, 69.8, 89.1, 102.1, 108.1, 114.6, 129.5, 131.8, 143.42, 144.5, 150.7, 163.6. HRMS (ESI) m/z: [M + H]^+^ calcd for C_29_H_41_N_2_O_5_, 497.3015; found 497.3004.

Compound **12b**: mp 71–73 °C. R_f_ = 0.66 (Petroleum ether: EtOAc = 1:4). ^1^H NMR (500 MHz, CDCl_3_) δ 0.78 (2 s, 6 H, 2 × CH_3_), 1.28–2.25 (m, 42H), 2.74 (m, 4 H, H-6 and H-6′), 3.35 (t, 2 H, *J* = 8.1 Hz, H-17 and H-17′), 3.44 (m, 4 H, 2 × OCH_2,_), 3.72 (t, 2 H, *J* = 7.2 Hz, NCH_2_), 3.85 (s, 6 H, 2 × OCH_3_), 3.93 (t, 2 H, *J* = 7.2 Hz, NCH_2_), 5.71 (d, 1 H, *J* = 7.8 Hz, H-5*^Ura^*), 6.63 (s, 2 H, H-4 and H-4′), 6.78 (s, 2 H, H-1 and H-1′), 7.09 (d, 1 H, *J* = 7.5 Hz, H-6*^Ura^*). ^13^C NMR (125 MHz, CDCl_3_) δ 11.7, 23.1, 26.0, 26.3, 26.8, 27.3, 28.2, 29.0, 38.1, 38.6, 41.2, 43.3, 44.3, 49.8, 50.3, 56.1, 69.8, 70.1, 89.0, 89.1, 101.6, 108.1, 114.6, 129.6, 131.8, 131.9, 142.1, 143.4, 143.5, 144.5, 151.4, 163.1. HRMS (ESI) m/z: [M + H]^+^ calcd for C_54_H_77_N_2_O_8_, 881.5680; found 881.5665.

#### 2.5.4. 17 β-[6-(5-Methyl-Uracil-3-yl)-Hexyloxy]-2-Methoxy-Estra-1,3,5(10)-Triene-3-ol (**13a**) and 1,3-Bis-{6-[2-Methoxy-3-Hydroxy-Estra-1,3,5(10)-Triene-17β-yl-oxy]-Hexyl}-5-Methyl-Uracil (**13b**)

Prepared from compound **8** (0.177 g, 0.381 mmol) and thymine (0.144 g, 1.14 mmol) according to general procedure III. The residue was purified by flash column chromatography to afford **13a** (white solid, 0.114 g, 59%) and **13b** (white solid, 0.072 g, 21%).

Compound **13a**: mp 80–82 °C. R_f_ = 0.29 (Petroleum ether: EtOAc = 1: 2). ^1^H NMR (500 MHz, CDCl_3_) δ 0.78 (s, 3 H, CH_3_), 1.92 (s, 3 H, CH_3_), 1.16–2.29 (m, 21 H), 2.74 (m, 2 H, H-6), 3.35 (t, 1 H, *J* = 8.4 Hz, H-17), 3.43 (m, 2 H, OCH_2_), 3.69 (td, 2 H, *J* = 6.5, 2.9 Hz, NCH_2_), 3.86 (s, 3 H, OCH_3_), 5.50 (s, 1 H, OH), 6.63 and 6.78 (2 s, each 1 H, H-4, H-1), 6.97 (s, 1 H, H-6*^Thy^*), 8.72 (s, 1 H, NH). ^13^C NMR (125 MHz, CDCl_3_) δ 11.7, 12.4, 15.3, 23.1, 25.9, 26.3, 28.2, 29.1, 30.0, 38.1, 38.6, 43.3, 44.2, 48.5, 50.3, 56.1, 66.1, 69.9, 70.4, 89.1, 108.1, 110.5, 114.6, 129.5, 131.8, 140.4, 143.4, 144.5, 150.7, 164.1. HRMS (ESI) m/z: [M + H]^+^ calcd for C_30_H_43_N_2_O_5_, 511.3172; found 511.3190.

Compound **13b**: mp 84–86 °C. R_f_ = 0.58 (Petroleum ether: EtOAc = 1: 2). ^1^H NMR (500 MHz, CDCl_3_) δ 0.79 (s, 6 H_,_ 2 × CH_3_), 1.92 (s, 3 H, CH_3_), 1.19–2.30 (m, 42 H), 2.76 (m, 4 H, 2 × H-6), 3.34–3.52 (m, overlapping, 6 H, 2 × H-17, 2 × OCH_2_), 3.71 (m, 2 H, NCH_2_), 3.87 (s, 6 H, 2 × OCH_3_), 3.95 (t, 2 H, *J* = 7.6 Hz, NCH_2_), 5.50 (s, 2 H, 2 × OH), 6.65 (s, 2 H, H-4 and H-4′), 6.79 (s, 2 H, H-1 and H-1′), 6.96 (s, 1 H, H-6 *^Thy^*). ^13^C NMR (125 MHz, CDCl_3_) δ 11.6, 13.1, 15.2, 23.0, 25.9, 26.7, 27.3, 27.59, 27.8, 28.2, 29.0, 29.1, 29.6, 29.7, 30.0, 30.1, 38.1, 38.6, 41.4, 41.5, 43.3, 43.4, 44.3, 49.4, 49.5, 50.2, 56.1, 66.0, 66.1, 69.9, 70.0, 70.4, 70.6, 89.0, 89.1, 108.1, 109.7, 114.6, 129.5, 131.8, 131.9, 138.3, 143.4, 144.5, 151.4, 163.8. HRMS (ESI) m/z: [M + Cl]^−^ calcd for C_55_H_78_ClN_2_O_8_, 929.5447; found 929.5464.

#### 2.5.5. 17 β-[8-(Uracil-3-yl)-Octyloxy]-2-Methoxy-Estra-1,3,5(10)-Triene-3-ol (**14a**) and 1,3-Bis-{8-[2-Methoxy-3-Hydroxy-Estra-1,3,5(10)-Triene-17β-yl-Oxy]-Octyl}-Uracil (**14b**)

Prepared from compound **9** (0.474 g, 0.960 mmol) and uracil (0.215 g, 1.92 mmol) according to general procedure III. The residue was purified by flash column chromatography to afford **14a** (white solid, 0.181 g, 36%) and **14b** (white solid, 0.192 g, 21%).

Compound **14a**: mp 68–69 °C. R_f_ = 0.14 (Petroleum ether: EtOAc = 1:1). ^1^H NMR (500 MHz, CDCl_3_) δ 0.78 (s, 3 H, CH_3_), 1.33–2.24 (m, 25 H), 2.76 (m, 2 H, H-6), 3.36 (t, 1 H, *J* = 8.4 Hz, H-17), 3.45 (m, 2 H, OCH_2_), 3.71 (t, 2 H, *J* = 7.3 Hz, NCH_2_), 3.85 (s, 3 H, OCH_3_), 5.51 (s, 1 H, OH), 5.70 (dd, 1 H, *J* = 7.9, 2.1 Hz, H-5*^Ura^*), 6.63 and 6.78 (2 s, each 1 H, H-4, H-1), 7.14 (d, 1 H, *J* = 7.9 Hz, H-6*^Ura^*), 8.98 (s, 1 H, NH). ^13^C NMR (125 MHz, CDCl_3_) δ 11.7, 26.2, 26.4, 26.8, 28.2, 29.0, 29.1, 29.2, 29.3, 30.2, 38.1, 38.6, 43.3, 44.2, 48.9, 50.3, 56.1, 70.1, 89.1, 102.1, 108.1, 114.6, 129.5, 131.8, 143.4, 144.5, 144.6, 150.7, 163.6. HRMS (ESI) m/z: [M − H]^−^ calcd for C_31_H_43_N_2_O_5_, 523.3172; found 523.3149.

Compound **14b**: mp 73–75 °C. R_f_ = 0.43 (Petroleum ether: EtOAc = 1:1). ^1^H NMR (500 MHz, CDCl_3_) δ 0.78 (2 s, 6 H_,_ 2 × CH_3_), 1.18–2.24 (m, 50 H), 2.76 (m, 4 H, H-6, H-6′), 3.36 (t, 2 H, *J* = 8.3 Hz, H-17, H-17′), 3.44 (m, 4 H, 2 × OCH_2,_), 3.71 (t, 2 H, *J* = 7.4 Hz, NCH_2_), 3.86 (s, 6 H, 2 × OCH_3_), 3.92 (t, 2 H, *J* = 7.6 Hz, NCH_2_), 5.44 (s, 2 H, 2 × OH), 5.71 (d, 1 H, *J* = 7.8 Hz, H-5*^Ura^*), 6.63 (s, 2 H, H-4 and H-4′), 6.78 (s, 2 H, H-1 and H-1′), 7.08 (d, 1 H, *J* = 7.8 Hz, H-6*^Ura^*). ^13^C NMR (125 MHz, CDCl_3_) δ 11.7, 23.0, 26.1, 26.7, 27.3, 28.2, 29.0, 29.2, 29.3, 29.4, 38.1, 38.6, 41.3, 43.3, 44.3, 49.8, 50.3, 56.0, 68.9, 70.2, 81.9, 89.0, 101.5, 108.0, 114.6, 129.5, 142.0, 143.4, 144.5, 151.4, 163.1. HRMS (ESI) m/z: [M + H]^+^ calcd for C_58_H_85_N_2_O_8_, 937.6306; found 937.6302.

#### 2.5.6. 17 β-[8-(5-Methyl-Uracil-3-yl)-Octyloxy]-2-Methoxy-Estra-1,3,5(10)-Triene-3-ol (**15a**) and 1,3-bis-{8-[2-Methoxy-3-Hydroxy-Estra-1,3,5(10)-Triene-17β-yl-oxy]-Octyl}-5-Methyl-Uracil (**15b**)

Prepared from compound **9** (0.415 g, 0.840 mmol) and thymine (0.321 g, 2.52 mmol) according to general procedure III. The residue was purified by flash column chromatography to afford **15a** (white solid, 0.156 g, 34%) and **15b** (white solid, 0.264 g, 33%).

Compound **15a**: mp 71–73 °C. R_f_ = 0.28 (Petroleum ether: EtOAc = 1:1). ^1^H NMR (500 MHz, CDCl_3_) δ 0.79 (s, 3 H, CH_3_), 1.92 (s, 3 H, CH_3_), 1.19–2.24 (m, 25 H), 2.76 (m, 2 H, H-6), 3.36 (t, 1 H, *J* = 8.3 Hz, H-17), 3.44 (m, 2 H, NCH_2_), 3.69 (t, 2 H, *J* = 7.4 Hz, OCH_2_), 3.85 (s, 3 H, OCH_3_), 5.50 (s, 1 H, OH), 6.63 and 6.78 (2 s, each 1 H, H-4, H-1), 6.97 (s, 1 H, H-6*^Thy^*), 8.63 (s, 1 H, NH). ^13^C NMR (125 MHz, CDCl_3_) δ 11.7, 12.3, 23.0, 26.4, 26.7, 27.3, 28.2, 29.1, 29.3, 30.1, 38.1, 38.6, 43.3, 44.2, 48.5, 50.3, 56.0, 70.1, 89.0, 108.1, 110.5, 114.6, 129.5, 131.8, 140.4, 143.4, 144.5, 150.7, 164.1. HRMS (ESI) m/z: [M − H]^−^ calcd for C_32_H_45_N_2_O_5_, 537.3328; found 537.3302.

Compound **15b**: mp 73–75 °C. R_f_ = 0.61 (Petroleum ether: EtOAc = 1:1). ^1^H NMR (500 MHz, CDCl_3_) δ 0.78 (s, 6 H_,_ 2 × CH_3_), 1.92 (s, 3 H, CH_3_), 1.17–2.24 (m, 50 H), 2.75 (m, 2 H, H-6), 3.36 (t, 2 H, *J* = 8.1 Hz, H-17, H-17′), 3.45 (m, 4 H, 2 × NCH_2_), 3.69 (t, 2 H, *J* = 7.4 Hz, OCH_2_), 3.86 (s, 6 H, 2 × OCH_3_), 3.93 (t, 2 H, *J* = 7.4 Hz, OCH_2_), 5.45 (s, 2 H, 2 × OH), 6.63 (s, 2 H, H-4 and H-4′), 6.79 (s, 2 H, H-1 and H-1′), 6.94 (s, 1 H, H-6 *^Thy^*). ^13^C NMR (125 MHz, CDCl_3_) δ 11.6, 13.1, 23.0, 26.1, 26.7, 27.0, 27.3, 28.2, 29.0, 29.1, 30.2, 38.1, 38.6, 41.5, 43.3, 44.2, 50.3, 56.0, 70.2, 77.0, 89.0, 108.0, 109.6, 114.6, 129.5, 131.9, 138.3, 143.4, 144.5, 151.3, 163.8. HRMS (ESI) m/z: [M + Cl]^−^ calcd for C_59_H_86_ClN_2_O_8_, 985.6073; found 985.6037.

### 2.6. General Procedure IV for Etherification at 3-Phenolic Hydroxyl

To a solution of 2-methoxyestradiol (1.65 mmol) and KOH (3.31 mmol) in dry DMF (6 mL), α, ω-dibromoalkane (4.96 mmol) were added under ice-water bath. After stirring at room temperature for 12 h, the mixture was diluted with H_2_O (30 mL) and extracted with EtOAc (3 × 20 mL). The combined organic layers were washed successively with 1 N HCl, saturated aqueous NaHCO_3_ and brine, dried (Na_2_SO_4_), filtered, and concentrated. The residue was purified by flash column chromatography.

#### 2.6.1. 2-Methoxy-3-(6-Bromohexyloxy)-Estra-1,3,5 (10)-Triene-17β-ol (**16**)

Prepared from 2-methoxyestradiol (0.500 g, 1.65 mmol) and 1,6-dibromohexane (0.760 mL, 4.96 mmol) according to general procedure IV. The residue was purified by flash column chromatography. Yield: 0.720 g, 94%, white solid, mp 99–100 °C. R_f_ = 0.50 (Petroleum ether: EtOAc = 3:1). ^1^H NMR (500 MHz, CDCl_3_) δ 0.76 (s, 3 H_,_ CH_3_), 1.17–2.31 (m, 21 H), 2.78 (m, 2 H, H-6), 3.42 (t, 2 H, *J* = 6.8 Hz, BrCH_2_), 3.73 (t, 1 H, *J* = 8.2 Hz, H-17), 3.84 (s, 3 H, OCH_3_), 3.98 (td, 2 H, *J* = 6.7, 2.3 Hz, OCH_2_), 6.59 and 6.83 (2 s, each 1 H, H-4, H-1). HRMS (ESI) m/z: [M + H]^+^ calcd for C_25_H_38_BrO_3_, 465.2004; found 465.1995.

#### 2.6.2. 2-Methoxy-3-(8-Bromooctyloxy)-Estra-1,3,5 (10)-Triene-17β-ol (**17**)

Prepared from 2-methoxyestradiol (0.100 g, 0.331 mmol) and 1,8-dibromooctane (0.180 mL, 0.992 mmol) according to general procedure IV. The residue was purified by flash column chromatography. Yield: 0.130 mg, 80%, white solid, mp 103–105 °C. R_f_ = 0.38 (Petroleum ether: EtOAc = 3:1). ^1^H NMR (500 MHz, CDCl_3_) δ 0.79 (s, 3 H_,_ CH_3_), 1.26–2.32 (m, 25 H), 2.90 (m, 2 H, H-6), 3.41 (t, 2 H, *J* = 6.8 Hz, BrCH_2_), 3.73 (t, 1 H, *J* = 8.5 Hz, H-17), 3.84 (s, 3 H, OCH_3_), 3.97 (td, 2 H, *J* = 6.8, 2.3 Hz, OCH_2_), 6.59 and 6.83 (2 s, each 1 H, H-4, H-1). HRMS (ESI) m/z: [M + H]^+^ calcd for C_27_H_42_BrO_3_, 493.2317; found 493.2310.

### 2.7. According to General Procedure III to Afford Compounds ***18**–**23a**/**23b***

#### 2.7.1. 3-[6-(1-β-d-Ribofuranosyluracil-3-yl)-Hexyloxy]-2-Methoxy-Estra-1,3,5(10)-Triene-17β-ol (**18**)

Prepared from compound **16** (0.086 g, 0.184 mmol) and uridine (0.136 g, 0.554 mmol) according to general procedure III. The residue was purified by flash column chromatography. Yield: 0.106 g, 91%, white solid, mp 99–100 °C. R_f_ = 0.60 (CH_2_Cl_2_:CH_3_OH = 6:1). ^1^H NMR (500 MHz, CDCl_3_) δ 0.82 (s, 3 H_,_ CH_3_), 1.21–2.22 (m, 21 H), 2.78 (m, 2 H, H-6), 3.33 (brs, 1 H, OH), 3.74 (t, 1 H, *J* = 8.6 Hz, H-17), 3.83 (s, 3 H, OCH_3_), 3.84–3.99 (m, 6 H, OCH_2,_ NCH_2_ and H-5′′), 4.20 (brs, 1 H, H-2′′), 4.33 (brs, 2 H, H-3′′and OH), 4.38 (brs, 1 H, H-4′′), 5.68 (d, 1 H, *J* = 3.2 Hz, H-1′′), 5.76 (d, 1 H, *J* = 8.0 Hz, H-5*^Uri^*), 6.62 and 6.82 (2 s, each 1 H, H-4, H-1), 7.67 (d, 1 H, *J* = 8.0 Hz, H-6*^Uri^*). ^13^C NMR (125 MHz, CDCl_3_) δ 11.3, 13.2, 14.2, 18.4, 21.1, 23.1, 26.5, 27.4, 29.2, 30.6, 33.6, 36.7, 38.8, 41.1, 44.2, 50.0, 56.3, 60.4, 61.9, 68.9, 70.6, 75.0, 81.9, 85.7, 93.4, 105.3, 109.7, 113.7, 130.0, 132.4, 138.8, 147.2, 151.7, 162.6. HRMS (ESI) m/z: [M + H]^+^ calcd for C_34_H_49_N_2_O_9_, 629.3438; found 629.3443.

#### 2.7.2. 3-[8-(1-β-d-Ribofuranosyluracil-3-yl)-Octyloxy]-2-Methoxy-Estra-1,3,5(10)-Triene-17β-ol (**19**)

Prepared from compound **17** (0.130 g, 0.279 mmol) and uridine (0.205 g, 0.838 mmol) according to general procedure III. The residue was purified by flash column chromatography. Yield: 0.150 g, 82%, white solid, mp 93–95 °C. R_f_ = 0.45 (CH_2_Cl_2_:CH_3_OH = 6:1). ^1^H NMR (500 MHz, CDCl_3_) δ 0.78 (s, 3 H_,_ CH_3_), 1.09–2.29 (m, 25 H), 2.78 (m, 2 H, H-6), 3.73 (t, 1 H, *J* = 8.4 Hz, H-17), 3.82 (s, 3 H, OCH_3_), 3.80–3.97 (m, overlapping, 6 H, OCH_2_, NCH_2_ and H-5′′), 4.17 (brs, 1 H, H-2′′), 4.31–4.38 (m, 2 H, H-3′′ and H-4′′), 5.67 (d, 1 H, *J* = 3.2 Hz, H-1′′), 5.75 (d, 1 H, *J* = 8.1 Hz, H-5*^Uri^*), 6.58 and 6.81 (2 s, each 1 H, H-4, H-1), 7.65 (d, 1 H, J = 8.1 Hz, H-6*^Uri^*). ^13^C NMR (125 MHz, CDCl_3_) δ 11.1, 23.1, 25.8, 26.5, 26.8, 27.3, 27.4, 29.0, 29.1, 29.2, 30.5, 36.7, 38.8, 41.2, 43.2, 44.2, 56.3, 61.9, 69.1, 70.6, 75.1, 81.9, 85.6, 93.3, 101.9, 109.7, 113.7, 128.9, 132.3, 138.7, 146.5, 147.2, 151.7, 162.6. HRMS (ESI) m/z: [M + H]^+^ calcd for C_36_H_53_N_2_O_9_, 657.3751; found 657.3745.

#### 2.7.3. 3-[6-(Uracil-3-yl)-Hexyloxy]-2-Methoxy-Estra-1,3,5(10)-Triene-17β-ol (**20a**) and 1,3-Bis-{6-[2-Methoxy-17β-Hydroxy-Estra-1,3,5(10)-Triene-3-yl-Oxy]-Hexyl}-Uracil (**20b**)

Prepared from compound **16** (0.120 g, 0.258 mmol) and uracil (0.087 g, 0.774 mmol) according to general procedure III. The residue was purified by flash column chromatography to afford **20a** (white solid, 0.043 g, 34%) and **20b** (white solid, 0.042 g, 19%).

Compound **20a**: mp 99–101 °C. R_f_ = 0.18 (Petroleum ether: EtOAc = 1:4). ^1^H NMR (500 MHz, CDCl_3_) δ 0.79 (s, 3 H_,_ CH_3_), 1.15–2.35 (m, 21 H), 2.78 (m, 2 H, H-6), 3.74 (m, 3 H, H-17, NCH_2_), 3.83 (s, 3 H, OCH_3_), 3.97 (td, 2 H, *J* = 6.5, 2.4 Hz, OCH_2_), 5.68 (d, 1 H, *J* = 7.9 Hz, H-5*^Ura^*), 6.59 and 6.83 (2 s, each 1 H, H-4, H-1), 7.15 (d, 1 H, *J* = 7.9 Hz, H-6*^Ura^*), 9.12 (s, 1 H, NH). ^13^C NMR (125 MHz, CDCl_3_) δ 11.1, 23.1, 25.6, 26.1, 26.4, 26.5, 27.4, 28.6, 28.9, 29.0, 30.6, 36.8, 38.8, 43.3, 44.3, 48.8, 50.0, 68.7, 69.5, 81.9, 102.1, 109.7, 113.8, 128.9, 132.5, 144.5, 147.3, 150.8, 163.8. HRMS (ESI) m/z: [M + H]^+^ calcd for C_29_H_41_N_2_O_5_, 497.3015; found 497.3021.

Compound **20b**: mp 93–94 °C. R_f_ = 0.34 (Petroleum ether: EtOAc = 1:4). ^1^H NMR (500 MHz, CDCl_3_) δ 0.78 (s, 6 H, 2 × CH_3_), 1.61–2.30 (m, 42 H), 2.78 (m, 4 H, H-6, H-6′), 3.72 (m, overlapping, 4 H, 2 × H-17 and NCH_2_), 3.94 (s, 6 H, 2 × OCH_3_), 3.95 (m, 6 H, NCH_2_ and 2 × OCH_2_), 5.68 (d, 1 H, *J* = 7.8 Hz, H-5*^Ura^*), 6.58 (s, 2 H, H-4 and H-4′), 6.81 and 6.82 (2 s, each 1 H, H-1 and H-1′), 7.08 (d, 1 H, *J* = 7.8 Hz, H-6*^Ura^*). ^13^C NMR (125 MHz, CDCl_3_) δ 11.1, 23.1, 24.8, 25.6, 25.7, 26.1, 26.5, 26.7, 27.6, 29.0, 29.1, 30.6, 35.6, 36.7, 38.8, 41.2, 43.3, 44.2, 49.9, 50.0, 56.2, 56.3, 68.6, 68.8, 81.9, 101.6, 109.5, 109.6, 113.6, 128.8, 132.2, 132.4, 142.1, 146.4, 146.5, 147.3, 151.4, 163.1. HRMS (ESI) m/z: [M + H]^+^ calcd for C_54_H_77_N_2_O_8_, 881.5680; found 881.5709.

#### 2.7.4. 3-[6-(5-Methyl-Uracil-3-yl)-Hexyloxy]-2-Methoxy-Estra-1,3,5(10)-Triene-17β-ol (**21a**) and 1,3-bis-{6-[2-Methoxy-17β-Hydroxy-Estra-1,3,5(10)-Triene-3-yl-oxy]-Hexyl}-5-Methyl-Uracil (**21b**)

Prepared from compound **16** (0.110 g, 0.236 mmol) and thymine (0.089 g, 0.708 mmol) according to general procedure III. The residue was purified by flash column chromatography to afford **21a** (white solid, 0.043 g, 40%) and **21b** (white solid, 0.058 g, 27%). 

Compound **21a**: mp 82–84 °C. R_f_ = 0.37 (Petroleum ether: EtOAc = 1:3). ^1^H NMR (500 MHz, CDCl_3_) δ 0.77 (s, 3 H_,_ CH_3_), 1.91 (s, 3 H_,_ CH_3_), 1.16–2.29 (m, 21 H), 2.77 (m, 2 H, H-6), 3.71 (m, 3 H, H-17, NCH_2_), 3.82 (s, 3 H, OCH_3_), 3.96 (td, 2 H, *J* = 6.5, 2.9 Hz, OCH_2_), 6.58 and 6.82 (2 s, each 1 H, H-4, H-1), 6.97 (s, 1 H, H-6*^Thy^*), 8.88 (s, 1 H, NH). ^13^C NMR (125 MHz, CDCl_3_) δ 11.1, 12.4, 23.1, 25.6, 26.1, 26.5, 27.4, 29.0, 29.2, 30.6, 36.8, 38.8, 43.3, 44.2, 48.5, 45.0, 56.2, 68.6, 81.9, 109.5, 110.6, 113.6, 128.8, 132.4, 140.5, 146.4, 147.3, 150.8, 164.3. HRMS (ESI) m/z: [M + H]^+^ calcd for C_30_H_43_N_2_O_5_, 511.3172; found 511.3174.

Compound **21b**: mp 79–81 °C. R_f_ = 0.56 (Petroleum ether: EtOAc = 1:3). ^1^H NMR (500 MHz, CDCl_3_) δ 0.78 (s, 6 H_,_ 2 × CH_3_), 1.91 (s, 3 H, CH_3_), 1.19–2.30 (m, 42 H), 2.78 (m, 4 H, H-6, H-6′), 3.66 (t, *J* = 8.3 Hz, 2 × H-17), 3.69 (m, 2 H, NH_2_), 3.82 (s, 6 H, 2 × OCH_3_), 3.96 (m, 6 H, NH_2_ and 2 × OCH_2_), 6.57 (s, 2 H, H-4 and H-4′), 6.82 (s, 2 H, H-1 and H-1′), 6.94 (s, 1 H, H-6*^Thy^*). ^13^C NMR (125 MHz, CDCl_3_) δ 11.1, 13.1, 23.1, 25.7, 26.5, 27.4, 29.0, 29.2, 30.6, 32.7, 36.8, 38.8, 41.4, 43.3, 44.3, 49.4, 50.0, 56.2, 62.9, 68.6, 68.8, 81.9, 113.6, 128.8, 147.3, 163.8. HRMS (ESI) m/z: [M + H]^+^ calcd for C_55_H_79_N_2_O_8_, 895.5836; found 895.5841.

#### 2.7.5. 3-[8-(Uracil-3-yl)-Octyloxy]-2-Methoxy-Estra-1,3,5(10)-Triene-17β-ol (**22a**) and 1,3-Bis-{8-[2-Methoxy-17β-Hydroxy-Estra-1,3,5(10)-Triene-3-yl-Oxy]-Octyl}-Uracil (**22b**)

Prepared from compound **17** (0.280 g, 0.567 mmol) and uracil (0.192 g, 1.70 mmol) according to general procedure III. The residue was purified by flash column chromatography to afford **22a** (white solid, 0.146 g, 49%) and **22b** (white solid, 0.110 g, 21%).

Compound **22a**: mp 68–70 °C. R_f_ = 0.47 (Petroleum ether: EtOAc = 1:4). ^1^H NMR (500 MHz, CDCl_3_) δ 0.78 (s, 3 H_,_ CH_3_), 1.15–2.35 (m, 25 H), 2.78 (m, 2 H, H-6), 3.71 (m, 3 H, H-17, NCH_2_), 3.83 (s, 3 H, OCH_3_), 3.96 (td, 2 H, *J* = 6.8, 2.3 Hz, OCH_2_), 5.69 (d, 1 H, *J* = 7.9 Hz, H-5*^Ura^*), 6.58 and 6.82 (2 s, each 1 H, H-4, H-1), 7.14 (d, 1 H, *J* = 7.9 Hz, H-6*^Ura^*), 8.96 (s, 1 H, NH). ^13^C NMR (125 MHz, CDCl_3_) δ 11.1, 23.1, 25.9, 26.4, 26.5, 27.4, 29.0, 29.1, 29.2, 29.3, 30.6, 36.8, 38.8, 43.3, 44.2, 48.9, 50.0, 55.3, 68.9, 81.9, 102.1, 109.5, 113.6, 128.8, 132.3, 144.5, 146.5, 147.3, 150.7, 163.6. HRMS (ESI) m/z: [M + H]^+^ calcd for C_31_H_45_N_2_O_5_, 525.3328; found 525.3323.

Compound **22b**: mp 74–76 °C. R_f_ = 0.59 (Petroleum ether: EtOAc = 1:4). ^1^H NMR (500 MHz, CDCl_3_) δ 0.79 (s, 6 H, 2 × CH_3_), 1.17–2.31 (m, 50 H), 2.79 (m, 4 H, H-6, H-6′), 3.73 (m, 4 H, 2 × H-17 and NCH_2_), 3.84 (s, 6 H, 2 × OCH_3_), 3.96 (m, 6 H, NCH_2_ and 2 × OCH_2_), 6.59 (s, 2 H, H-4 and H-4′), 6.82 and 6.83 (2 s, each 1 H, H-1 and H-1′), 5.71 (d, 1 H, *J* = 7.8 Hz, H-5*^Ura^*), 7.09 (d, 1 H, *J* = 7.8 Hz, H-6*^Ura^*). ^13^C NMR (125 MHz, CDCl_3_) δ 11.1, 23.1, 25.9, 26.4, 27.4, 29.2, 29.3, 30.6, 36.8, 38.8, 43.3, 44.2, 50.0, 56.3, 65.6, 68.9, 81.9, 101.6, 109.6, 113.6, 128.8, 132.3, 142.1, 146.6, 147.3, 151.4, 163.2. HRMS (ESI) m/z: [M + H]^+^ calcd for C_58_H_85_N_2_O_8_, 937.6306; found 937.6293.

#### 2.7.6. 3-[8-(5-Methyl-Uracil-3-yl)-Octyloxy]-2-Methoxy-Estra-1,3,5(10)-Triene-17β-ol (**23a**) and 1,3-bis-{8-[2-Methoxy-17β-Hydroxy-Estra-1,3,5(10)-Triene-3-yl-Oxy]-octyl}-5-Methyl-Uracil (**23b**)

Prepared from compound **17** (0.280 g, 0.567 mmol) and thymine (0.216 g, 1.70 mmol) according to general procedure III. The residue was purified by flash column chromatography to afford **23a** (white solid, 0.168 g, 55%) and **23b** (white solid, 0.120 g, 22%).

Compound **23a**: mp 79–81 °C. R_f_ =0.30 (Petroleum ether: EtOAc = 1:3). ^1^H NMR (500 MHz, CDCl_3_) δ 0.78 (s, 3 H_,_ CH_3_), 1.91 (s, 3 H_,_ CH_3_), 1.21–2.29 (m, 25 H), 2.78 (m, 2 H, H-6), 3.67 (m, 2 H, NCH_2_), 3.73 (t, 1 H, *J* = 8.5 Hz, H-17), 3.82 (s, 3 H, OCH_3_), 3.96 (td, 2 H, *J* = 6.8, 2.2 Hz, OCH_2_), 6.58 and 6.81 (2 s, each 1H, H-4, H-1), 6.97 (d, 1 H, *J* = 1.2 Hz, H-6 *^Thy^*). ^13^C NMR (125 MHz, CDCl_3_) δ 11.1, 12.3, 23.1, 25.9, 26.3, 26.5, 27.3, 29.1, 29.2, 29.3, 30.6, 36.7, 38.8, 43.3, 44.2, 48.5, 50.0, 56.2, 68.9, 77.0, 81.9, 109.6, 110.5, 113.6, 128.8, 132.3, 140.4, 146.5, 147.2, 150.7, 164.1. HRMS (ESI) m/z: [M + H]^+^ calcd for C_32_H_47_N_2_O_5_, 539.3485; found 539.3481.

Compound **23b**: mp 75–76 °C. R_f_ = 0.50 (Petroleum ether: EtOAc = 1:3). ^1^H NMR (500 MHz, CDCl_3_) δ 0.78 (s, 6 H, 2 × CH_3_), 1.92 (s, 3 H, CH_3_), 1.16–2.30 (m, 50 H), 2.77 (m, 4 H, H-6,H-6′), 3.71 (m, 4 H, 2 × H-17 and NCH_2_), 3.83 (s, 6 H, 2 × OCH_3_), 3.93–3.96 (m, 6 H, NCH_2_ and 2 × OCH_2_), 6.59 (s, 2 H, H-4 and H-4′), 6.82 (s, 2 H, H-1 and H-1′), 6.94 (s, 1 H, H-6 *^Thy^*). HRMS (ESI) m/z: [M + H]^+^ calcd for C_59_H_87_N_2_O_8_, 951.6462; found 951.6458.

### 2.8. In Vitro Cytotoxicity

Human breast adenocarcinoma MCF-7 cell lines, human breast cancer MDA-MB-231 cell lines, and the normal human liver L-O2 cell lines were provided by Nanjing KeyGen Biotech Co. Ltd. (Nanjing, China). MCF-7 cells were cultured in a base medium (90% RPMI-1640 and 10% FBS) at 37 °C, 5% CO_2_ and a saturated humidity atmosphere. MDA-MB-231 cells were cultured in a base medium (90% L-50 and 10% FBS) at 37 °C, 5% CO_2_ and a saturated humidity atmosphere. L-O2 cells were cultured in a base medium (90% DMEM and 10% FBS) at 37 °C, 5% CO_2_ and a saturated humidity atmosphere.

The cytotoxicity of the tested compounds was determined using the 3-(4,5-dimethyl-2-thiazolyl)-2,5-diphenyl-2-*H*-tetrazolium bromide (MTT) assay [40]. Cells were seeded in 96-well plates and incubated in a 5% CO_2_ incubator at 37 °C. When the cells adhered, compounds at different concentrations were added to every well. After incubation for another 72 h, MTT solution (5 mg/mL) was added into each well and cells were incubated for an additional 4 h. The viable cells were stained with MTT and scanned with an electrophotometer at 570 nm. Each concentration treatment was done in triplicate wells. The half maximal inhibitory concentration (IC_50_) values were estimated by fitting the inhibition data to a dose-dependent curve using a logistic derivative equation.

### 2.9. Hoechst 33,258 Staining

The nuclear morphological changes and apoptotic effects induced by compound **11/12a** were detected using Hoechst 33,258 staining. Cells grown on a sterile cover slip were treated with **11/12a** for 72 h in six-well tissue culture plates. Cells were collected and mounted on a slide, fixed for 30 min with 4% paraformaldehyde, and washed three times with PBS. Then, cells were stained with Hoechst 33,258 (2 mg/mL in PBS) for 10 min at room temperature in the dark. The cells were subsequently washed three times with PBS and observed with a fluorescence microscope.

### 2.10. Mitochondrial Membrane Potential Analysis

MCF-7 cells were incubated in triplicate and treated with compound **11/12a** for 72 h. The cells were washed with PBS and stained with JC-1 dye (Keygen, KGA601) under dark conditions according to the manufacturer’s instructions. After incubation, the cells were washed twice with PBS, and the percentage of cells with collapsed or healthy mitochondrial membrane potentials (MMP) was monitored by flow cytometry analysis (FACS Calibur Becton-Dickinson, San Jose, California, USA).

### 2.11. Cell Cycle Assay

MCF-7 cells at a density of 5 × 10^5^ cells/well were seeded in 6-well plates and incubated at 37 °C in a humidified 5% CO_2_ incubator for 24 h. Exponentially growing cells were treated with compound **11**/**12a** (0, 5, 10, and 20 μM) for 72 h. Then, the cells were harvested and fixed with 70% precooled ethanol at 4 °C for 12 h. Later, the cells were centrifuged and washed with cold PBS. 100 μL of RNase A was added, and then bathed in water for 30 min at 37 °C. Subsequently, cells were stained by 400 μL of propidium iodide (PI) for 30 min at 4 °C in the dark. Finally, cellular DNA was measured using a flow cytometry (FACS Calibur Becton-Dickinson, San Jose, California, USA).

### 2.12. Cell Apoptosis Analysis

MCF-7 cells at a density of 2 × 10^5^ cells/well were seeded on each well of 6-well plates and allowed to grow overnight. Then the cells were treated with compound **11**/**12a** (5, 10, and 20 μM) for 72 h, while cells without treatment were used as control group. The treated cells were trypsinized and washed with cold PBS twice, then centrifuged at 2000 rpm for 5 min and resuspended in 500 μL annexin V binding buffer. 5 μL of Annexin V- allophycocyanin (APC) was added, and the mixture was incubated in the dark at 25 °C for 15 min. Then, 5 μL of 7-AAD was added just prior to acquisition. Apoptosis was analyzed by Annexin V-APC and 7-AAD double staining by flow cytometry (FACS Calibur Becton-Dickinson, San Jose, Califormia, USA).

### 2.13. Immunohistochemical (IHC) Analysis

MCF-7 cells were seeded in culture plates and treated with compound **11/12a** (0, 5, 10, and 20 μM) for 72 h. Cells were collected and mounted on a slide, fixed for 30 min with 4% paraformaldehyde, and washed three times with PBS. 1% Triton X-100 was added to each cell sample, and it was permeabilized at room temperature for 10 min, then washed three times with PBS. Two drops of 3% H_2_O_2_-methanol solution were added to each cell section. After being blocked at room temperature for 10 min, the section was washed three times with PBS. Then, 50–100 μL of ready-to-use goat serum was added and cells were incubated for 20 min at room temperature. Before the cell section was incubated at 37 °C for 2 h in a wet box, 50–100 μL of a primary antibody (1:200 dilution) against estradiol receptor alpha (ERα) (Bs-1427R, Bioss Co. Ltd., Beijing, China) was added, and then washed three times with PBS. Subsequently, 50 μL of enhancer was added and cells were incubated for 30 min at room temperature, then washed three times with PBS. Fifty microliters of a universal IgG antibody-Fab fragment-HRP multimer was added dropwise, and the mixture was incubated at 37 °C for 30 min, then soaked and washed three times with PBS. After 2 drops of freshly prepared DAB (DAB-1031, MaXi Biotech Co. Ltd., FuZhou, China) solution were added to each cell section, the dyeing depth was observed under the microscope. The dyeing was stopped immediately if the dyeing depth is appropriate, and the cells gently rinsed with tap water for 15 min, then the color reaction was terminated with distilled water. Next, the cell sections were stained by hematoxylin dye solution for 10 min, and rinsed with distilled water. The cell sections were soaked in 70% ethanol, 85% ethanol, 95% ethanol, and absolute ethanol for 5 min respectively, then soaked twice in fresh xylene for 10 min each time. After drying, neutral gum was added to the cell sections and the coverslip was covered. Lastly, the expression situation of protein in tissue cells was observed under a light microscope, and three high-expression areas were selected, photographed, and stored.

### 2.14. Real-Time Polymerase Chain Reaction

RNA was isolated using Trizol Reagent (Invitrogen, Carlsbad, California, USA). cDNA was made via the manufacturer’s instructions with the first chain of a cDNA synthesizing kit (Fermentas, Lithuania). Real-time polymerase chain reaction (RT-PCR) was performed on a Da An Gene DA7600 Real Time machine using Da An Gene incorporation (ZhongShan, China). The 2^−ΔΔ*CT*^ method was used to calculate the relative levels of gene expression using Da An Gene DA7600 QPCR software v3.00 (Da An Gene). The GAPDH (human) expression was used as the internal control. A standard melting-curve cycle was used to confirm the quality of amplification. The reactions were performed in triplicate for each sample. The gene-specific primers were designed with the Primer5 BLAST software by GenScript Co., Ltd. (Nanjing, China):

1 GAPDH (human): primer (122 bp),
  - Sense primer: 5′ ATCATCCCTGCCTCTACTGG 3’
  - Antisense primer: 5′ GTCAGGTCCACCACTGACAC 3′
2 Apaf-1 (human): primer (134 bp),
  - Sense primer: 5′ TTCAGCAGAAGCTCTCCAAA 3′
  - Antisense primer: 5′ CCCTGGGAAACAACCTTCTA 3′
3 Bax (human): primer (112 bp),
  - Sense primer: 5 ′TTGCTTCAGGGTTTCATCC 3′
  - Antisense primer: 5′ GACACTCGCTCAGCTTCTTG 3′
4 Bcl-2 (human): primer (116 bp),
  - Sense primer: 5′ GGCCTCTGTTTGATTTCTCC 3′
  - Antisense primer: 5′ GCAGGCATGTTGACTTCACT 3′
5 Bcl-xL (human): primer (124 bp),
  - Sense primer: 5′ CTATGGGAACAATGCAGCAG 3′
  - Antisense primer: 5′ TGGTCATTTCCGACTGAAGA 3′
6 Caspase-3 (human): primer (131 bp),
  - Sense primer: 5′ AGCACTGGAATGACATCTCG 3′
  - Antisense primer: 5′ CGCATCAATTCCACAATTTC 3′
7 Caspase-8 (human): primer (122 bp),
  - Sense primer: 5′ CTCCAAATGCAAACTGGATG 3′
  - Antisense primer: 5′ TGTTGATTTGGGCACAGACT 3′
8 Caspase-9 (human): primer (136 bp),
  - Sense primer: 5′ AGACCCAGGTCCAGATGAAG 3′
  - Antisense primer: 5′ TTTCTGGGAAGGGACAGAAG 3′
9 Cytochrome c (human): primer (127 bp),
  - Sense primer: 5′ GACTCCTGACCTCGTGATCC 3′
  - Antisense primer: 5′ TCTGTGCCAACACAGACCTT 3′

### 2.15. Western Blotting

After the treatment of MCF-7 cells with compound **11**/**12a** (0, 5, 10, and 20 μM) for 72 h, the total proteins of MCF-7 cells were extracted and their concentrations were balanced to the same level by BCA protein assay reagent (KGP902, KeyGen Biotech Co. Ltd., Nanjing, China), followed by 8 min of protein denaturation with SDS loading buffer at 100 °C. Proteins were separated by sodium dodecyl sulfate-polyacrylamide electrophoresis (SDS-PAGE) and transferred onto polyvinylidene fluoride (PVDF) membranes, which were blocked with 5 mL of 5% fat-free dry milk in 1× Tris-buffered saline (TBS) containing 0.05% Tween 20 for 2 h. Then the membranes were incubated with Apaf-1 (Abcam, no: ab234436), Bax (Abcam, no: ab32503), Bcl-2 (Abcam, no: ab692), Bcl-xL (Abcam, no: ab32370), caspase-3 (Abcam, no: ab184787), caspase-8 (Abcam, no: ab25901), caspase-9 (Abcam, no: ab219590), cytochrome c (Abcam, no: ab133504), and β-actin (Abcam, no: ab8226) antibodies at 4 °C overnight, followed by treatment with secondary horseradish-peroxidase-conjugated anti-rabbitIgG (KeyGen Biotech Co. Ltd., no: KGAA35) for 2 h. Membranes were finally scanned in a ChemiDoc MP Imaging System (Bio-Rad) after 2 min incubation in Clarity Western ECL Substrate (Bio-Rad).

### 2.16. Modeling

The 3D QSAR study was generated by using 20 molecules from synthesis, 2-methoxyestradiol (**1**) and their negative logarithm of the IC_50_ value (pIC_50_) of MCF-7, performed with Surflex (Version 4.4) using leave-one-out cross validation method as previously described [41]. Twenty randomly selected molecules were calculated by Surflex-Quansa to build a 3D QSAR model (number of molecules to select for core multiple-alignment = 10) by leaving a single molecule out. The pIC_50_ of the left out one was predicted by the built model, and the pIC_50_ was calculated for each molecule by running this method 21 times.

## 3. Results and Discussion

### 3.1. Synthesis

The synthesis of hybrids **10**–**15b** and **18**–**23b** was commenced with 2-methoxyestradiol as outlined in Scheme 1 and Scheme 2. 2-Methoxyestradiol was reacted with benzyl chloride under the presence of sodium hydroxide to afford 3-phenolic hydroxyl protected compound **5** (82%). Then 17-hydroxyl was etherified with corresponding α,ω-dibromoalkanes under the alkali of sodium hydride to prepare compounds **6** (92%) and **7** (88%). This was followed by deprotection with 10% Pd/C under a hydrogen atmosphere to generate compounds **8** (88%) and **9** (90%). Finally, compounds **8** and **9** were, respectively, treated with uridine/uracil/thymine under potassium carbonate by means of nucleophilic substitution reaction to afford targeted hybrids **10**–**15b** at yields of 16–77% (Scheme 1). 2-Methoxyestradiol was also firstly etherified with the corresponding α,ω-dibromoalkanes under potassium hydroxide to generate compounds **16** (94%) and **17** (80%). Subsequently, a nucleophilic substitution reaction was carried out with uridine/uracil/thymine, respectively, under the presence of potassium carbonate to target hybrids **18**–**23b** at yields of 19–91% (Scheme 2). Targeted compounds **10**–**15b** and **18**–**23b** have been fully characterized by ^1^H- and ^13^C-NMR spectroscopy and high resolution mass spectrometry.

### 3.2. Biological Evaluation

#### 3.2.1. In Vitro Cytotoxicity

Human breast cancer cell lines including the estrogen receptor-positive cell lines (MCF-7) and the estrogen receptor-negative cell lines (MDA-MB-231), were found to be sensitive to the growth-inhibitory actions of 2-methoxyestradiol [21,42]. Thus, the target compounds **10**–**15b** and **18**–**23b** were investigated for their anti-tumor activity in vitro with MCF-7 and MDA-MB-231 cell lines using MTT assay [40]. 5-FU, which is used clinically as a medication in the cancer field, was used as a positive control in this study. In order to understand their selectivity towards cancerous cell lines vs. a healthy cell line, these compounds were also evaluated for their inherent toxicity in healthy human liver cell line (L-O2). The results are presented in Table 1.

In the sub-group of uridine derivatives (**10**, **11**, **18**, and **19**), these compounds exhibited significant anti-proliferative activities at IC_50_ values from 3.89 µM to 16.68 µM. Compared with 5-FU, these uridine derivatives have similar activity against MCF-7 cell line, but much better activity against the MDA-MB-231 cell line. Meanwhile compared with 2-methoxyestradiol, these uridine derivatives possess slightly less activity against both MCF-7 and MDA-MB-231 cell lines. It is interesting that all these uridine derivatives exhibited much lower activities than 2-methoxyestradiol against healthy cell line L-O2. Particularly, compound **11** (eight carbon chain linked to the 17-*O* position of 2-methoxyestradiol) exhibited almost 7-fold selectivity towards cancerous cell lines (MCF-7 and MDA-MB-231) *vs* the healthy cell line (L-O2).

In the sub-groups of uracil/thymine derivatives (**12a**–**15b** and **20a**–**23b**), only dual-access compound **21b** exhibited good anti-proliferative activities and about 4-fold selectivity towards cancerous cell lines (MCF-7 and MDA-MB-231) *vs* healthy cell line (L-O2). All the other dual-access products (**12b**–**15b**, **20b**, **22b**, and **23b**) showed complete loss of anti-proliferative activity. Meanwhile all the single-access products (**12a**–**15a** and **20a**–**23a**) showed significant anti-proliferative activities at IC_50_ values from 7.34 µM to 19.32 µM and good toleration of selectivity towards cancerous cell lines. In these uracil/thymine derivatives, compound **12a** possesses good anti-proliferative activity and the highest selectivity (about 6-fold).

With these biological and selectivity results, hybrids **11** and **12a** were chosen for further study of their mechanism on MCF-7 cell line.

#### 3.2.2. Hoechst 33258 Staining

**Figure 2:**
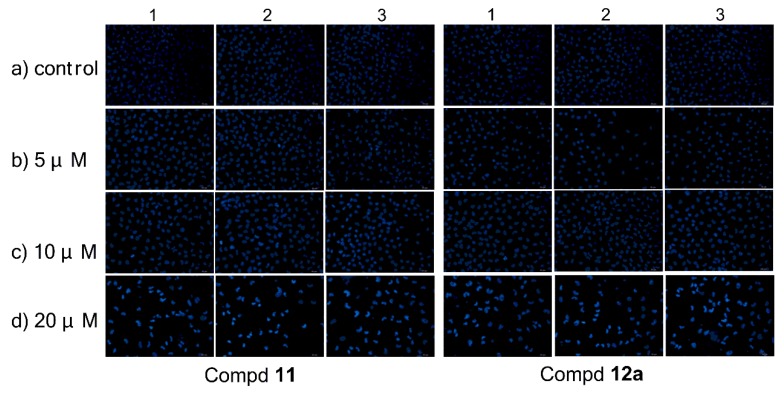
Morphological observation of MCF-7 cells stained by Hoechst 33258, treated by compound **11/12a** for 72 h in three separated experiments, respectively.

MCF-7 cells were treated with compound **11**/**12a** at 0, 5, 10, and 20 μM respectively for 72 h, followed by membrane-permeable Hoechst 33258 staining (Figure 2). The changes on the cell morphology could be clearly observed after exposure to each compound under the concentration gradient. It was found that the MCF-7 cells treated with higher concentrations of compound **11**/**12a** displayed stronger blue fluorescence (Figure 2) indicating the appearance of apoptosis.

#### 3.2.3. Mitochondrial Membrane Potential Analysis

**Figure 3:**
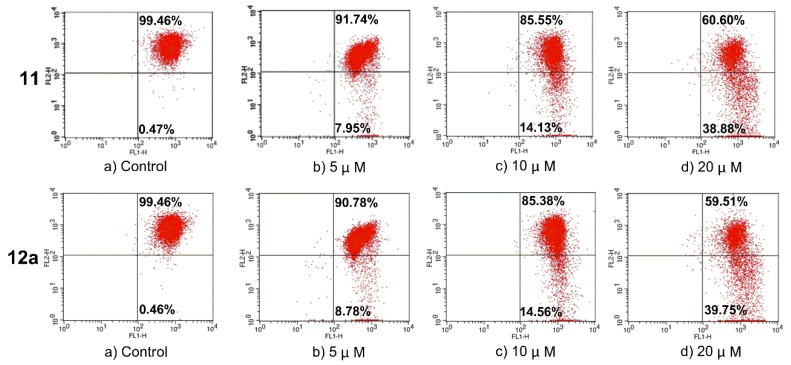
Changes in mitochondrial membrane potential caused by compound **11**/**12a** on MCF-7 cells. Incubation with different concentrations of **11**/**12a** in MCF-7 cells for 72 h prior to staining with JC-1 dye; the number of cells with collapsed mitochondrial membrane potentials was determined by flow cytometry.

As the mitochondrial membrane potential (MMP) is regarded to be characteristic for cell apoptosis, the membrane potential assay was carried out by flow cytometry to discuss whether compound **11**/**12a** might induce apoptosis on MCF-7 cells via mitochondrial pathway. The fluorescent probe JC-1 was used to detect the changes in mitochondrial membrane potentials. As shown in Figure 3, when MCF-7 cells were treated with **11** at concentrations of 0, 5, 10, and 20 μM for 72 h, the number of MCF-7 cells with collapsed MMP increased from 0.47% to 7.95%, 14.13%, and 38.88%, respectively. When MCF-7 cells were treated with **12a** at concentrations of 0, 5, 10, and 20 μM for 72 h, the number of MCF-7 cells with collapsed MMP increased from 0.46% to 8.78%, 14.56% and 39.75%, respectively. The results suggested that **11** and **12a** could cause mitochondrial depolarization of MCF-7 cells in the process of apoptosis.

#### 3.2.4. Cell Cycle Analysis

Compounds **11** and **12a** were examined for the effect on cell cycle distribution using a propidium iodide (PI) staining kit (KeyGen, KGA511). MCF-7 cells were treated respectively with compound **11/12a** (0, 5, 10, and 20 μM) for 72 h, then stained with PI and analyzed on a flow cytometry.

**Figure 4:**
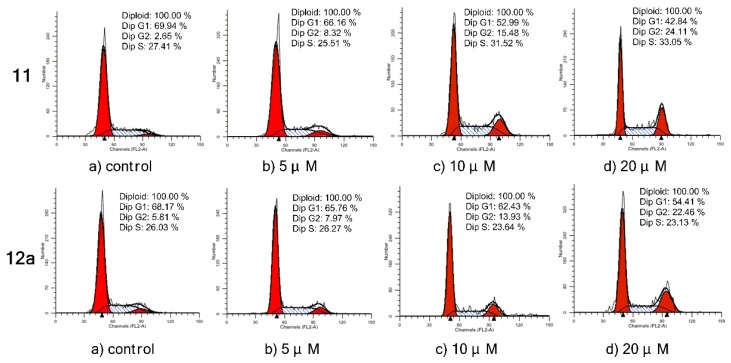
Inhibition of cell cycle progress on MCF-7 cells treated with compound **11/12a** for 72 h. Cells were fixed with ethanol and stained with propidium iodide. Cell cycle distribution was analyzed by a flow cytometry.

As shown in Figure 4, compound **11** led to a significant accumulation of cells at the G2 phase, depending on concentration, from 2.65% (control) to 8.32% (5 μM), 15.48% (10 μM), and 24.11% (20 μM), accordingly. At the same time, it reduced the cells at the G1 phase from 69.94 to 42.84%. The same phenomenon was observed on compound **12a**. Its ability to arrest the cell cycle at the G2 phase increased from 5.81% (control) to 7.97% (5 μM), 13.93% (10 μM), and 22.46% (20 μM) in a dose-dependent manner, while the G1 phase was decreased from 68.17 to 54.41%. These results demonstrated that both **11** and **12a** could induce cell cycle arrest at the G2 phase in a dose-dependent manner.

#### 3.2.5. Cell Apoptosis Analysis

**Figure 5:**
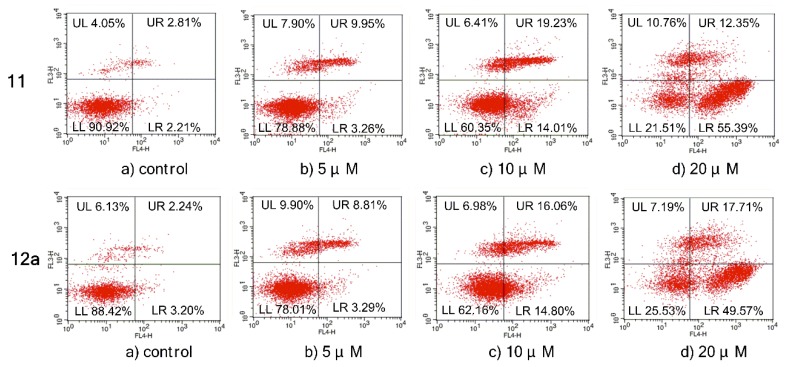
Apoptosis ratio detection by Annexin V-APC/7-AAD double staining assay, analyzed by a flow cytometry on MCF-7 cells treated with compound **11/12a** for 72 h, respectively. The UL area represents damaged cells appearing in the process of cell collection, the UR area represents necrotic cells and later period apoptotic cells, the LR area represents early apoptotic cells, and normal cells are located in the LL area.

The effects of compound **11**/**12a** on MCF-7 cell apoptosis were further evaluated in an Annexin V-APC/7-AAD double staining assay and analyzed by a flow cytometry. As shown in Figure 5, the percentage of apoptotic cells after 72 h treatment was only 5.02% in the control group without any treatments, while the total numbers of early and late apoptotic cells increased to 13.21%, 33.24%, and 67.74% after treatment with **11** at 5, 10, and 20 μM, respectively. A similar situation occurred in **12a**. Compared with the control (5.44%, early and late apoptosis), the apoptosis rates increased when treated with **12a**, which gradually increased from 12.1% to 30.86% and 67.28% at concentrations of 5, 10, and 20 μM, respectively. These results confirmed that compound **11**/**12a** effectively induced cell apoptosis in MCF-7 cells in a dose-dependent manner.

#### 3.2.6. Immunohistochemical Analysis

**Figure 6:**
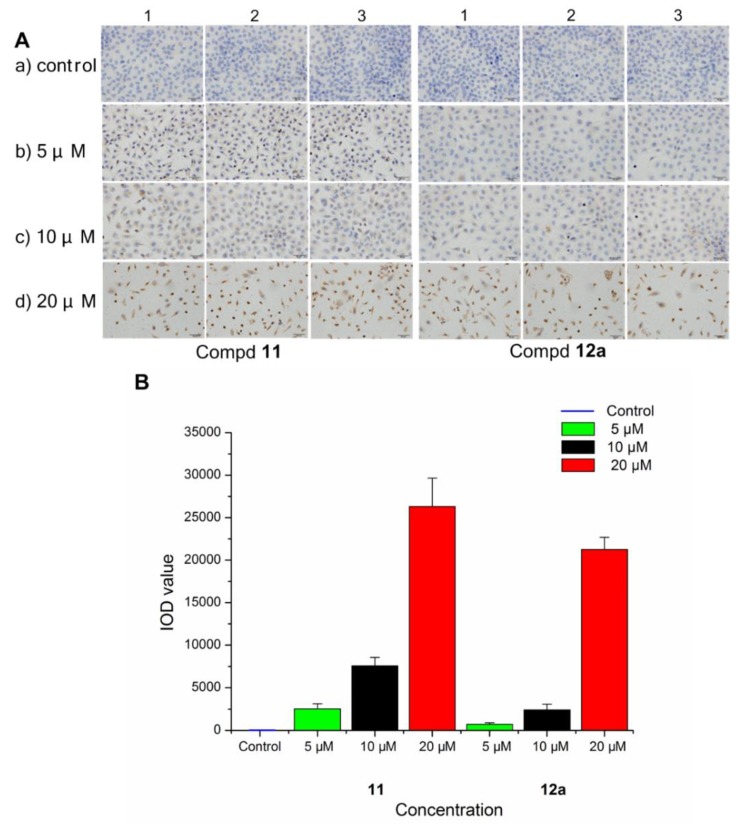
Immunohistochemistry of ERα in MCF-7 breast cancer cells treated with compound **11**/**12a** at different concentrations (5, 10, and 20 μM), respectively, and a group without treatment as the control. (**A**) The observed phenomenon with the treatment of compound **11** and **12a** in MCF-7 cells. (**B**) The IOD value detected in the treated MCF-7 cells.

To evaluate the interaction between the selected compounds and estradiol receptor alpha (ERα), immunohistochemical (IHC) analysis was carried out on MCF-7 breast cancer cells by an EliVision plus kit (kit-9902, MaXi Biotech Co. Ltd., FuZhou, China). With the increase in the concentration of compound **11**/**12a**, the color of the nuclear became deeper (Figure 6A), and the IOD value became much higher and far exceeded the control (Figure 6B). These results indicated that there was some interaction (specific binding) between the selected compounds (**11** and **12a**) and the ERα, which increased in a dose-dependent manner.

#### 3.2.7. Real-Time Polymerase Chain Reaction Analysis

**Figure 7:**
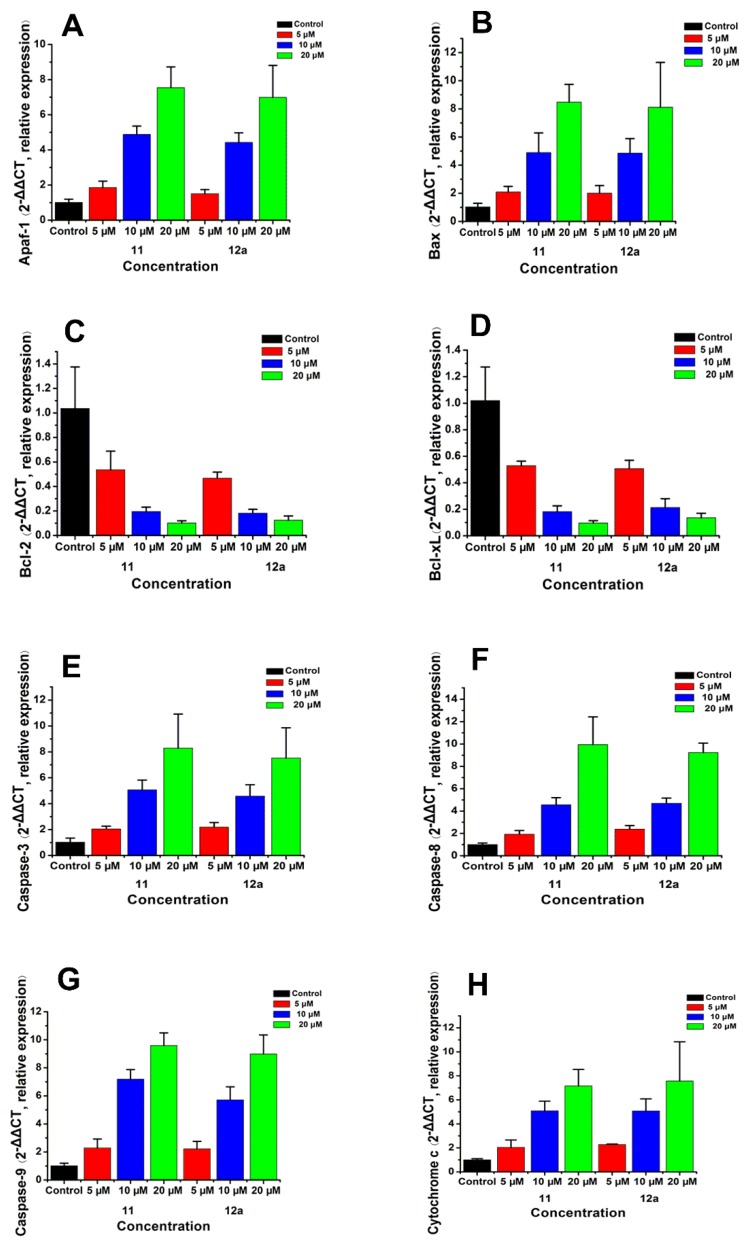
mRNA expressions of Apaf-1 (**A**), Bax (**B**), Bcl-2 (**C**), Bcl-Xl (**D**) caspase-3 (**E**), caspase-8 (**F**), caspase-9 (**G**), and cytochrome c (**H**) by RT-PCR in MCF-7 breast cancer cells treated with compound **11**/**12a** at concentrations of 5, 10, and 20 μM, respectively. GAPDH was used as a loading control.

In order to investigate the intrinsic mechanism of the apoptosis effects produced by the selected compounds at gene level, real-time PCR (RT-PCR) analysis was conducted on MCF-7 breast cancer cells treated with compound **11** and **12a** at concentrations of 5, 10, and 20 μM, respectively. GAPDH was used as a loading control. As shown in Figure 7, the expression levels of Apaf-1, Bax, caspase-3, caspase-8, caspase-9, and cytochrome c were upregulated after treatment with **11**/**12a**, respectively; meanwhile, the expression levels of Bcl-2 and Bcl-xL were downregulated. In addition, they were significantly enhanced/decreased with the increase in concentration.

#### 3.2.8. Western Blotting Assay

**Figure 8:**
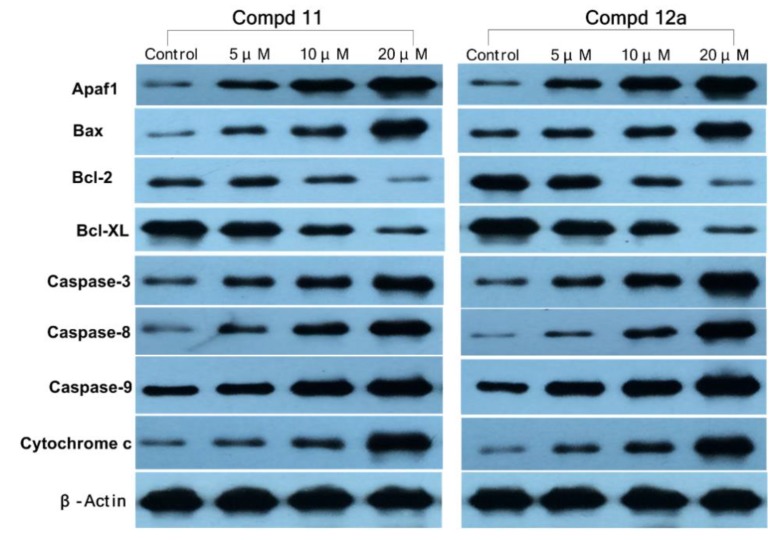
The protein expressions of Apaf-1, Bax, Bcl-2, Bcl-xL, caspase-3, caspase-8, caspase-9, and cytochrome c in MCF-7 cells treated with compounds **11**/**12a** in western blotting assay. Cells were treated with various concentrations (0, 5, 10, and 20 µM) of **11**/**12a** for 72 h.

To further explore the apoptosis mechanism of MCF-7 cells caused by compounds **11** and **12a**, we examined the expressions of Apaf-1, Bax, Bcl-2, Bcl-xL, caspase-3, caspase-8, caspase-9, and cytochrome c proteins after treatment with **11**/**12a** (Figure 8) by western blotting assay. *β*-Actin was used as a loading control. As shown in Figure 8, the expressions of Apaf-1, Bax, caspase-3, caspase-8, caspase-9, and cytochrome c proteins increased in a dose-dependent manner after treatment with **11**/**12a**, whereas the expressions of Bcl-2 and Bcl-xL were obviously decreased with the increase in the compound’s concentration. It was indicated that both of compounds **11** and **12a** could up regulate the expression of Apaf-1, Bax, and cytochrome c downregulate the expression of Bcl-2 and Bcl-xL and increase the expression of caspases (caspase-3, caspase-8, and caspase-9) in MCF-7 cells. Therefore, compounds **11** and **12a** could induce intrinsic apoptosis in MCF-7 cells and this result is consistent with previous results.

### 3.3. Modeling

A 3D QSAR study was generated by using these 21 molecules and their pIC_50_ value of MCF-7, performed with Surflex-Quansa using the leave-one-out cross validation method as previously described [41]. The molecules with low activity (pIC_50_ of MCF-7 < 4) were in correlation with predicted values. For the most 13 active molecules, the predicted pIC_50_ values are very similar compared with the experimental pIC_50_ values indicating a good QSAR model (leave-one-out cross-validation correlation coefficient q^2^ = 0.54, and standard error of estimate SEE = 0.2; Table 2).

**Figure 9:**
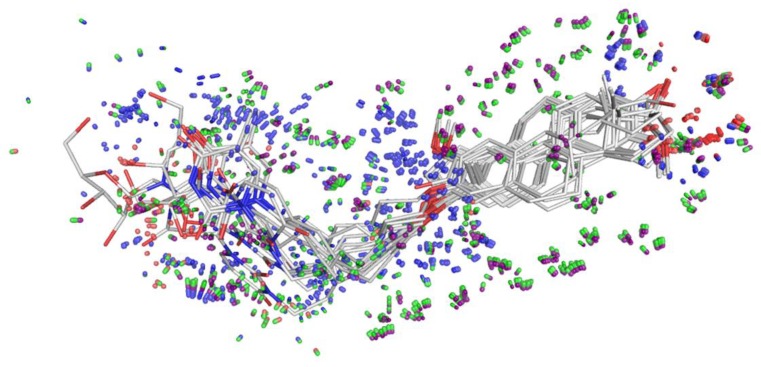
Alignment of molecules (line) and pocket-field (50% transparent stick). The pocket-field: sticks colored purple—coulombic response; green—steric; red—acceptor; blue—donor constructed by using ligand structure and MCF-7 activity information. Image generated by pymol [43].

Figure 9 demonstrates the learned response function values of the active set of 13 molecules (pIC_50_ of MCF-7 value > 4) in their final best optimal poses. The pocket-field (sticks colored purple—coulombic response; green—steric; red—acceptor; blue—donor) was generated automatically by using ligand structure and MCF-7 activity information. These molecules were well aligned, especially for the common part of 2-methoxyestradiol.

This model helps us to predict the changes in biological activity caused by the ligand structural modifications, demonstrates ligand-receptor interactions that may be associated with our experimentally observed SAR, and finally provides us with guidance for further compound design and optimization.

## 4. Conclusions

In the present study, a total of 20 targeted 2-methoxyestradiol-uridine/uracil/thymine hybrids were synthesized and characterized inspired by the biological activities of 2-methoxyestradiol and uridine/uracil/thymine in anti-tumor field. The results indicated that compounds **11** and **12a** could inhibit proliferation and possessed good selectivity between breast tumor cell lines (MCF-7 and MDA-MB-231) and a human normal cell line (L-O2) compared with the other derivatives. Moreover, hybrids **11** and **12a** could cause depolarized mitochondria and induce cell cycle arrest at the G2 phase in a dose-dependent manner. Furthermore, **11** and **12a** could induce apoptosis through upregulation of Apaf-1, Bax, caspase-3, caspase-8, caspase-9, and cytochrome c, and downregulation of Bcl-2 and Bcl-xL either at gene or protein level. Additionally, **11** and **12a** could specific bind to the estradiol receptor alpha in a dose-dependent manner. All in all, the 2-methoxyestradiol derivatives—**11** and **12a** exhibit good potential antitumor properties as apoptosis inducers and are expected to be further developed.

## Figures and Tables

**Table 1:** Evaluation of target compounds against different cell lines.

Compounds	IC_50_ (μM) ^a^		
	MCF-7	MDA-MB-231	L-O2
**10**	16.68 ± 0.30	13.17 ± 1.09	35.19 ± 0.42
**11**	13.89 ± 0.16	13.97 ± 1.08	>100
**12a**	11.25 ± 0.46	13.03 ± 0.38	69.34 ± 4.37
**13a**	16.64 ± 1.26	13.29 ± 0.16	51.36 ± 1.47
**14a**	9.22 ± 0.21	8.00 ± 0.41	17.52 ± 0.69
**15a**	12.12 ± 0.45	19.32 ± 1.54	52.62 ± 1.82
**12b**	>100	>100	No ^b^
**13b**	>100	>100	No
**14b**	>100	>100	No
**15b**	>100	>100	No
**18**	6.21 ± 0.37	3.89 ± 0.36	21.26 ± 0.66
**19**	7.87 ± 0.42	10.44 ± 0.07	32.83 ± 0.65
**20a**	17.92 ± 0.22	13.96 ± 1.69	38.51 ± 1.87
**21a**	9.97 ± 0.39	7.34 ± 0.56	28.16 ± 0.57
**22a**	10.26 ± 0.37	9.58 ± 0.98	25.86 ± 1.82
**23a**	8.75 ± 0.22	12.73 ± 0.22	25.48 ± 1.86
**20b**	>100	>100	No
**21b**	28.38 ± 1.76	23.76 ± 1.45	>100
**22b**	>100	>100	No
**23b**	>100	>100	No
**1**	1.51 ± 0.04	1.86 ± 0.02	4.70 ± 0.45
**5-FU**	8.87 ± 0.59	61.87 ± 2.23	>100

^a^ IC_50_ values are presented as the mean ± SD (standard deviation) from three separated experiments. ^b^ No inhibition detected.

**Table 2:** Comparison of predicted pIC_50_ by 3D QSAR with experimental values.

Compounds	IC_50_ (μM) ^a^ for MCF-7	pIC_50_^b^ for MCF-7	Predicted pIC_50_^c^
**10**	16.68 ± 0.30	4.78	4.84
**11**	13.89 ± 0.16	4.86	4.72
**12a**	11.25 ± 0.46	4.95	5.43
**13a**	16.64 ± 1.26	4.78	5.98
**14a**	9.22 ± 0.21	5.04	5.96
**15a**	12.12 ± 0.45	4.92	4.51
**12b**	>100	<4	1.94
**13b**	>100	<4	1.58
**14b**	>100	<4	−1.71
**15b**	>100	<4	1.64
**18**	6.21 ± 0.37	5.21	6.16
**19**	7.87 ± 0.42	5.10	5.79
**20a**	17.92 ± 0.22	4.75	4.78
**21a**	9.97 ± 0.39	5.00	6.04
**22a**	10.26 ± 0.37	4.99	5.65
**23a**	8.75 ± 0.22	5.06	4.74
**20b**	>100	<4	3.91
**21b**	28.38 ± 1.76	4.55	−0.80
**22b**	>100	<4	−1.05
**23b**	>100	<4	−0.07
**1**	1.51 ± 0.04	5.82	7.10

^a^ IC_50_ values are presented as the mean ± SD (standard deviation) from three separated experiments. ^b^ pIC_50_ = −log(IC_50_). ^c^ Predicted pIC_50_ is calculated by the leave-one-out cross validation method (Surflex-quansa).

## Supplementary Materials

The following are available online at https://www.mdpi.com/2218-273X/10/1/123/s1, NMR and HRMS spectra of compounds **5**–**23b**.

## References

[1] Pribluda V.S., Gubish E.R., LaVallee T.M., Treston A., Swartz G.M., Green S.J. (2000). 2-Methoxyestradiol: An Endogenous Antiangiogenic and Antiproliferative Drug Candidate. Cancer Metastasis Rev..

[2] Siebert A.E., Sanchez A.L., Dinda S., Moudgil V.K. (2011). Effects of estrogen metabolite 2-methoxyestradiol on tumor suppressor protein p53 and proliferation of breast cancer cells. Syst. Biol. Reprod. Med..

[3] Chua Y.S., Chua Y.L., Hagen T. (2010). Structure activity analysis of 2-methoxyestradiol analogues reveals targeting of microtubules as the major mechanism of antiproliferative and proapoptotic activity. Mol. Cancer Ther..

[4] Nakagawa-Yagi Y., Ogane N., Inoki Y., Kitoh N. (1996). The endogenous estrogen metabolite 2-methoxyestradiol induces apoptotic neuronal cell death in vitro. Life Sci..

[5] Lakhani N.J., Sarkar M.A., Venitz J., Figg W.D. (2003). 2-Methoxyestradiol, a promising anticancer agent. Pharmacother. J. Hum. Pharmacol. Drug Ther..

[6] Dahut W.L., Lakhani N.J., Gulley J.L., Arlen P.M., Kohn E.C., Kotz H., McNally D., Parr A., Nguyen D., Yang S.X. (2006). Phase I clinical trial of oral 2-methoxyestradiol, an antiangiogenic and apoptotic agent, in patients with solid tumors. Cancer Biol. Ther..

[7] Harrison M.R., Hahn N.M., Pili R., Oh W.K., Hammers H., Sweeney C., Kim K.M., Perlman S., Arnott J., Sidor C. (2011). A phase II study of 2-methoxyestradiol (2ME2) NanoCrystal dispersion (NCD) in patients with taxane-refractory, metastatic castrate-resistant prostate cancer (CRPC). Investig. New Drugs.

[8] Verenich S., Gerk P.M. (2010). Therapeutic promises of 2-methoxyestradiol and its drug disposition challenges. Mol. Pharm..

[9] Fotsis T., Zhang Y., Pepper M.S., Adlercreutz H., Montesano R., Nawroth P.P., Schweigerer L. (1994). The endogenous oestrogen metabolite 2-methoxyoestradiol inhibits angiogenesis and suppresses tumour growth. Nature.

[10] Sattler M., Quinnan L.R., Pride Y.B., Gramlich J.L., Chu S.C., Even G.C., Kraeft S.K., Chen L.B., Salgia R. (2003). 2-Methoxyestradiol alters cell motility, migration, and adhesion. Blood.

[11] Mueck A.O., Seeger H. (2010). 2-Methoxyestradiol—biology and mechanism of action. Steroids.

[12] Lee Y.M., Ting C.M., Cheng Y.K., Fan T.P., Wong R.N.S., Lung M.L., Mak N.K. (2008). Mechanisms of 2-methoxyestradiol-induced apoptosis and G2/M cell-cycle arrest of nasopharyngeal carcinoma cells. Cancer Lett..

[13] Kumar A.P., Garcia G.E., Slaga T.J. (2001). 2-methoxyestradiol blocks cell-cycle progression at G2/M phase and inhibits growth of human prostate cancer cells. Mol. Carcinog..

[14] Li Y., Xia Z.L., Chen L.B. (2011). HIF-1-alpha and survivin involved in the anti-apoptotic effect of 2ME2 after global ischemia in rats. Neurol. Res..

[15] Chen C.H., Hu Q., Yan J.H., Lei J.L., Qin L.H., Shi X.Z., Luan L.J., Yang L., Wang K., Han J.Y. (2007). Multiple effects of 2ME2 and D609 on the cortical expression of HIF-1alpha and apoptotic genes in a middle cerebral artery occlusion-induced focal ischemia rat model. J. Neurochem..

[16] Lee J.Y., Jee S.B., Park W.Y., Choi Y.J., Kim B., Kim Y.H., Jun D.Y., Kim Y.H. (2014). Tumor suppressor protein p53 promotes 2-methoxyestradiol-induced activation of Bak and Bax, leading to mitochondria-dependent apoptosis in human colon cancer HCT116 cells. J. Microbiol. Biotechnol..

[17] Zhou X., Liu C.F., Lu J.H., Zhu L.B., Li M. (2018). 2-Methoxyestradiol inhibits hypoxia-induced scleroderma fibroblast collagen synthesis by phosphatidylinositol 3-kinase/Akt/mTOR signalling. Rheumatology.

[18] Tao H., Mei J.J., Tang X.Y. (2019). The anticancer effects of 2-methoxyestradiol on human huh7 cells in vitro and in vivo. Biochem. Biophys. Res. Commun..

[19] Lee J.S., Ahn C., Kang H.Y., Jeung E.B. (2017). Effect of 2-methoxyestradiol on SK-LMS-1 uterine leiomyosarcoma cells. Oncol. Lett..

[20] Nair S.K., Verma A., Thomas T.J., Chou T.C., Gallo M.A., Shirahata A., Thomas T. (2007). Synergistic apoptosis of MCF-7 breast cancer cells by 2-methoxyestradiol and bis(ethyl)norspermine. Cancer Lett..

[21] Han G.Z., Liu Z.J., Shimoi K., Zhu B.T. (2005). Synergism between the anticancer actions of 2-methoxyestradiol and microtubule-disrupting agents in human breast cancer. Cancer Res..

[22] Stander B.A., Marais S., Vorster C.J.J., Joubert A.M. (2010). In vitro effects of 2-methoxyestradiol on morphology, cell cycle progression, cell death and gene expression changes in the tumorigenic MCF-7 breast epithelial cell line. J. Steroid Biochem..

[23] Mueck A.O., Seeger H., Huober J. (2004). Chemotherapy of breast cancer-additive anticancerogenic effects by 2-methoxyestradiol?. Life Sci..

[24] Botes M., Jurgens T., Riahi Z., Visagie M., van Vuuren R.J., Joubert A.M., van den Bout I. (2018). A novel non-sulphamoylated 2-methoxyestradiol derivative causes detachment of breast cancer cells by rapid disassembly of focal adhesions. Cancer Cell Int..

[25] Wang C., Li L.L., Fu D.Y., Qin T.T., Ran Y.G., Xu F., Du X.R., Gao H.Y., Sun S.J., Yang T.J. (2019). Discovery of chalcone-modified estradiol analogs as antitumour agents that Inhibit tumour angiogenesis and epithelial to mesenchymal transition. Eur. J. Med. Chem..

[26] Shah J.H., Agoston G.E., Suwandi L., Hunsucker K., Pribluda V., Zhan X.H., Swartz G.M., La Vallee T.M., Treston A.M. (2009). Synthesis of 2- and 17-substituted estrone analogs and their antiproliferative structure–activity relationships compared to 2-methoxyestradiol. Bioorg. Med. Chem..

[27] Jourdan F., Leese M.P., Dohle W., Hamel E., Ferrandis E., Newman S.P., Purohit A., Reed M.J., Potter B.V.L. (2010). Synthesis, antitubulin, and antiproliferative SAR of analogues of 2-methoxyestradiol-3,17-O,O-bis-sulfamate. J. Med. Chem..

[28] Peyrat J.F., Brion J.D., Alami M. (2012). Synthetic 2-methoxyestradiol derivatives: Structure-activity relationships. Curr. Med. Chem..

[29] Yamaguchi M., Matsuda A., Ichikawa S. (2015). Synthesis of isoxazolidine-containing uridine derivatives as caprazamycin analogues. Org. Biomol. Chem..

[30] Paszkowska J., Kral K., Bieg T., Żaba K., Węgrzyk K., Jaśkowiak N., Molinaro A., Silipo A., Wandzik I. (2015). Synthesis and biological evaluation of 5′-glycyl derivatives of uridine as inhibitors of 1,4-β-galactosyltransferase. Bioorg. Chem..

[31] Krol E., Wandzik I., Gromadzka B., Nidzworski D., Rychlowska M., Matlacz M., Tyborowska J., Szewczyk B. (2013). Anti-influenza A virus activity of uridine derivatives of 2-deoxy sugars. Antivir. Res..

[32] Komor R., Pastuch-Gawolek G., Krol E., Szeja W. (2018). Synthesis and preliminary evaluation of biological activity of glycoconjugates analogues of acyclic uridine derivatives. Molecules.

[33] Pałasz A., Cież D. (2015). In search of uracil derivatives as bioactive agents. Uracils and fused uracils: Synthesis, biological activity and applications. Eur. J. Med. Chem..

[34] Afifi H., Ebead A., Pignatelli J., Lee-Ruff E. (2015). Synthesis of cyclobutane nucleosides 2-preparation of thymine and uracil analogues. Nucleosides Nucleotides Nucleic Acids.

[35] Lönnberg T., Hutchinson M., Rokita S. (2015). Selective Alkylation of C-Rich Bulge Motifs in Nucleic Acids by Quinone Methide Derivatives. Chem. Eur. J..

[36] Usha S., Selvaraj S. (2014). Structure-wise discrimination of cytosine, thymine, and uracil by proteins in terms of their nonbonded interactions. J. Biomol. Struct. Dyn..

[37] Cheng K.G., Su C.H., Huang J.Y., Liu J., Zheng Y.T., Chen Z.F. (2016). Conjugation of uridine with oleanolic acid derivatives as potential antitumor agents. Chem. Biol. Drug Des..

[38] Cheng K.G., Su C.H., Huang J.Y., Wang H.S., Liu J., Zheng Y.T., Chen Z.F. (2016). Synthesis and cytotoxic evaluation of several oleanolic acid–uracil/thymine conjugates. MedChemComm.

[39] Lao K.J., Wang Y.J., Chen M.Q., Zhang J.J., You Q.D., Xiang H. (2017). Design, synthesis and biological evaluation of novel 2-methoxyestradiol analogs as dual selective estrogen receptor modulators (SERMs) and antiangiogenic agents. Eur. J. Med. Chem..

[40] Ciapetti G., Cenni E., Pratelli L., Pizzoferrato A. (1993). In vitro evaluation of cell/biomaterial interaction by MTT assay. Biomaterials.

[41] Cleves A.E., Jain A.N. (2018). Quantitative surface field analysis: Learning causal models to predict ligand binding affinity and pose. J. Comput. Aided Mol. Des..

[42] Liu Z.J., Zhu B.T. (2004). Concentration-dependent mitogenic and antiproliferative actions of 2-methoxyestradiol in estrogen receptor-positive human breast cancer cells. J. Steroid Biochem..

[43] DeLano W.L. (2002). Pymol: An open-source molecular graphics tool. CCP4 Newsl. Protein Crystallogr..

