# Supplementary material for: Design, Synthesis, and Evaluation of Novel 2-Methoxyestradiol Derivatives as Apoptotic Inducers through an Intrinsic Apoptosis Pathway
Source: Biomolecules. 2020 Jan 10;10(1):123. doi: 10.3390/biom10010123 (PMC7023064; doi:10.3390/biom10010123)

Supplementary Materials

Design, Synthesis and Evaluation of Novel 2-Methoxyestradiol Derivatives as Apoptotic Inducers Through Intrinsic Apoptosis Pathway

Li-Xin Sheng [1,†], Jiang-Yu Zhang [1,†], Li Li [1], Xiao Xie [2,*], Xiao-An Wen [3] and Ke-Guang Cheng [1,*]

1. State Key Laboratory for the Chemistry and Molecular Engineering of Medicinal Resources, School of Chemistry and Pharmacy of Guangxi Normal University, 541004, Guilin, P. R. China
2. Univ. Lille, Inserm, Institut Pasteur de Lille, U1177 - Drugs and Molecules for living Systems, F-59000 Lille, France
3. Jiangsu Key Laboratory of Drug Discovery for Metabolic Diseases and State Key Laboratory of Natural Medicines, Center of Drug Discovery, China Pharmaceutical University, 24 Tongjia Xiang, Nanjing 210009, China

* Correspondence: xiao.xie0417@gmail.com (X.X); kgcheng2008@gmail.com (K.-G.C.) Tel.: +86-0773-2120958(K.-G.C.)

† These authors contributed equally to this work.

Table of Contents

^{1}H NMR spectrum of **5** 4

HRMS spectrum of **5** 5

^{1}H NMR spectrum of **6** 6

HRMS spectrum of **6** 7

^{1}H NMR spectrum of **7** 8

^{13}C NMR spectrum of **7** 9

HRMS spectrum of **7** 10

^{1}H NMR spectrum of **8** 11

^{13}C NMR spectrum of **8** 12

HRMS spectrum of **8** 13

^{1}H NMR spectrum of **9** 14

^{13}C NMR spectrum of **9** 15

HRMS spectrum of **9** 16

^{1}H NMR spectrum of **10** 17

^{13}C NMR spectrum of **10** 18

HRMS spectrum of **10** 19

^{1}H NMR spectrum of **11** 20

^{13}C NMR spectrum of **11** 21

HRMS spectrum of **11** 22

^{1}H NMR spectrum of **12a** 23

^{13}C NMR spectrum of **12a** 24

HRMS spectrum of **12a** 25

^{1}H NMR spectrum of **12b** 26

^{13}C NMR spectrum of **12b** 27

HRMS spectrum of **12b** 28

^{1}H NMR spectrum of **13a** 29

^{13}C NMR spectrum of **13a** 30

HRMS spectrum of **13a** 31

^{1}H NMR spectrum of **13b** 32

^{13}C NMR spectrum of **13b** 33

HRMS spectrum of **13b** 34

^{1}H NMR spectrum of **14a** 35

^{13}C NMR spectrum of **14a** 36

HRMS spectrum of **14a** 37

^{1}H NMR spectrum of **14b** 38

^{13}C NMR spectrum of **14b** 39

HRMS spectrum of **14b** 40

^{1}H NMR spectrum of **15a** 41

^{13}C NMR spectrum of **15a** 42

HRMS spectrum of **15a** 43

^{1}H NMR spectrum of **15b** 44

^{13}C NMR spectrum of **15b** 45

HRMS spectrum of **15b** 46

^{1}H NMR spectrum of **16** 47

HRMS spectrum of **16** 48

^{1}H NMR spectrum of **17** 49
HRMS spectrum of **17** 50
^{1}H NMR spectrum of **18** 51
^{13}C NMR spectrum of **18** 52
HRMS spectrum of **18** 53
^{1}H NMR spectrum of **19** 54
^{13}C NMR spectrum of **19** 55
HRMS spectrum of **19** 56
^{1}H NMR spectrum of **20a** 57
^{13}C NMR spectrum of **20a** 58
HRMS spectrum of **20a** 59
^{1}H NMR spectrum of **20b** 60
^{13}C NMR spectrum of **20b** 61
HRMS spectrum of **20b** 62
^{1}H NMR spectrum of **21a** 63
^{13}C NMR spectrum of **21a** 64
HRMS spectrum of **21a** 65
^{1}H NMR spectrum of **21b** 66
^{13}C NMR spectrum of **21b** 67
HRMS spectrum of **21b** 68
^{1}H NMR spectrum of **22a** 69
^{13}C NMR spectrum of **22a** 70
HRMS spectrum of **22a** 71
^{1}H NMR spectrum of **22b** 72
^{13}C NMR spectrum of **22b** 73
HRMS spectrum of **22b** 74
^{1}H NMR spectrum of **23a** 75
^{13}C NMR spectrum of **23a** 76
HRMS spectrum of **23a** 77
^{1}H NMR spectrum of **23b** 78

HRMS spectrum of **23b** 79

^{1}H NMR spectrum of **5**

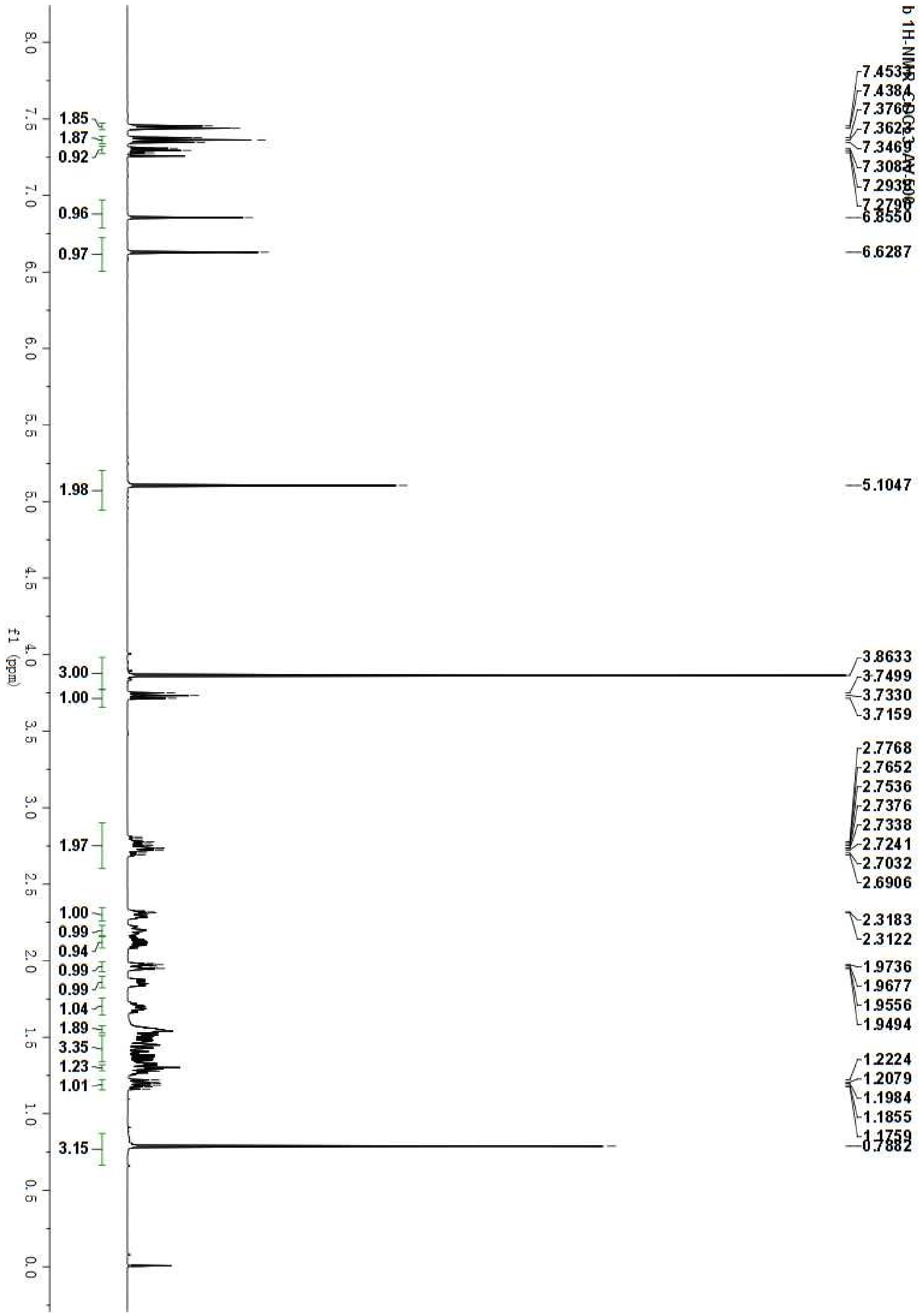

HRMS spectrum of **5**

**Sample Name**		**Position**	P2-A6	**Instrument Name**	Instrument 1	**User Name**	
**Inj Vol**	0.1	**InjPosition**		**SampleType**	Sample	**IRM Calibration Status**	Success
**Data Filename**	b-p.d	**ACQ Method**		**Comment**		**Acquired Time**	

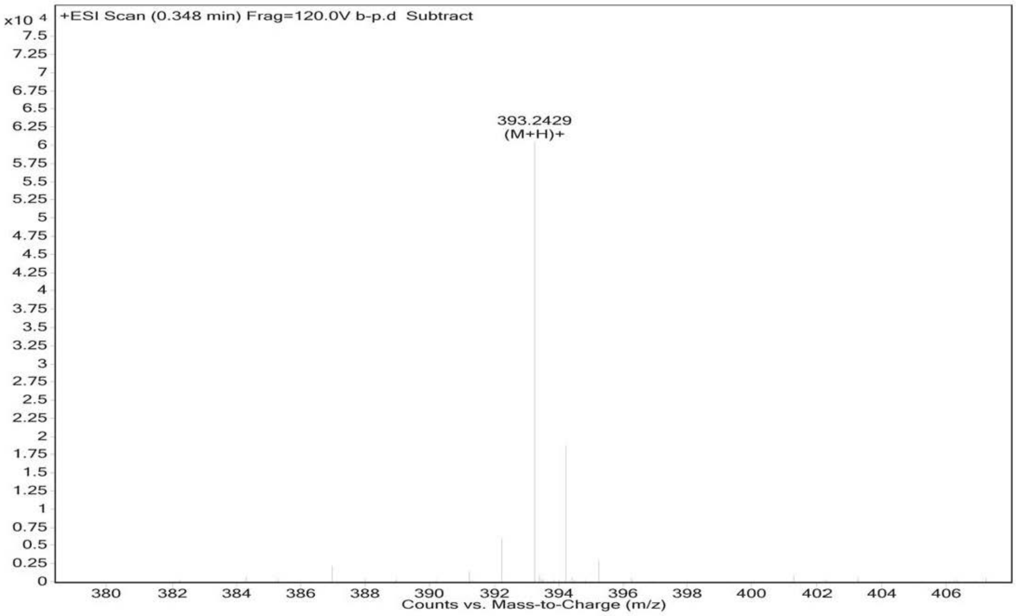

^{1}H NMR spectrum of **6**

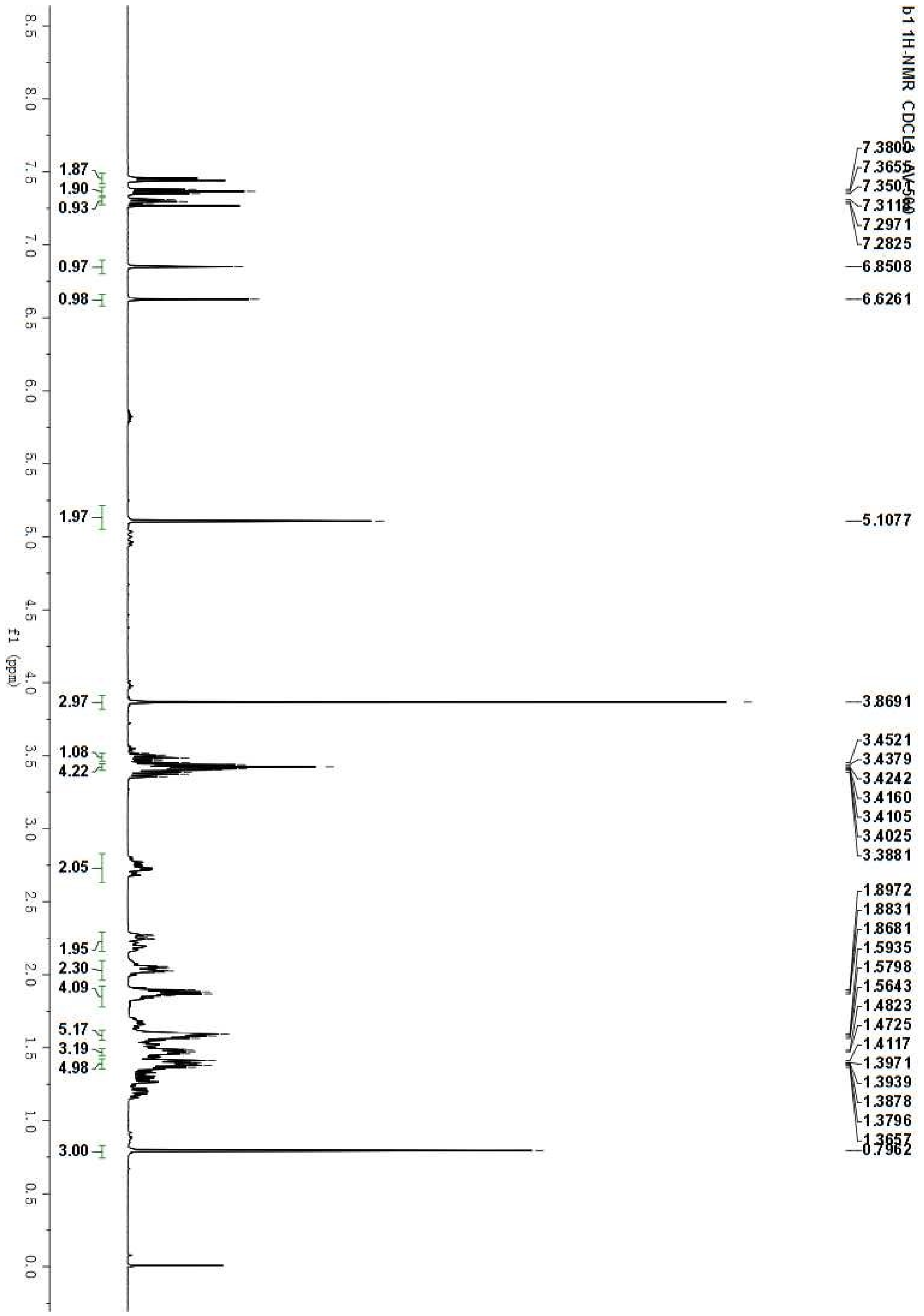

HRMS spectrum of **6**

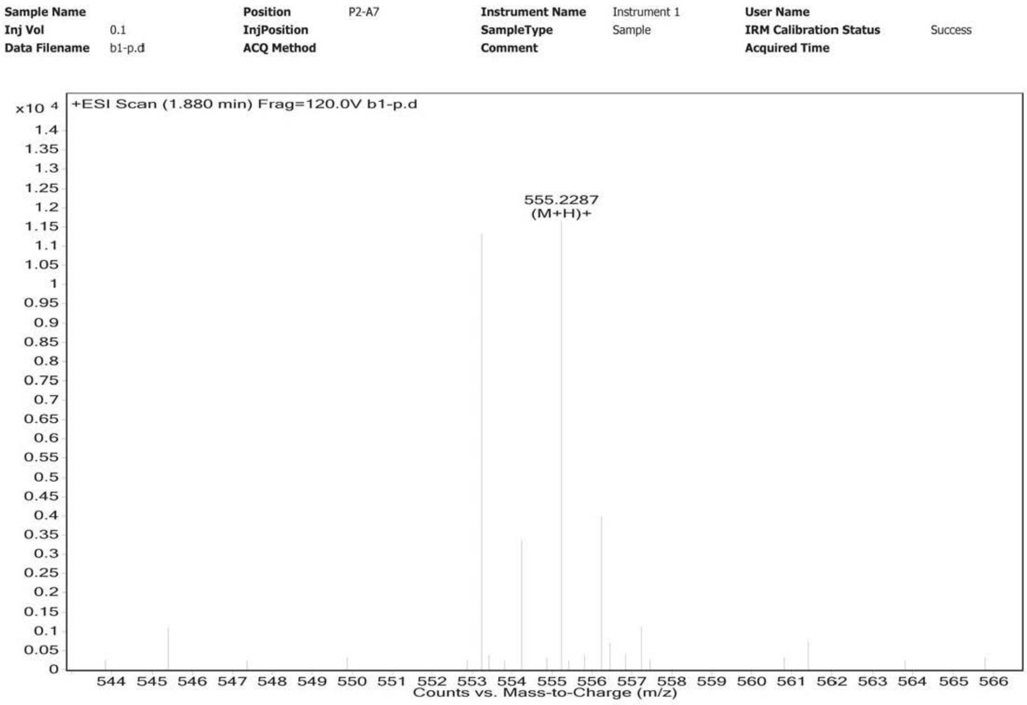

^{1}H NMR spectrum of **7**

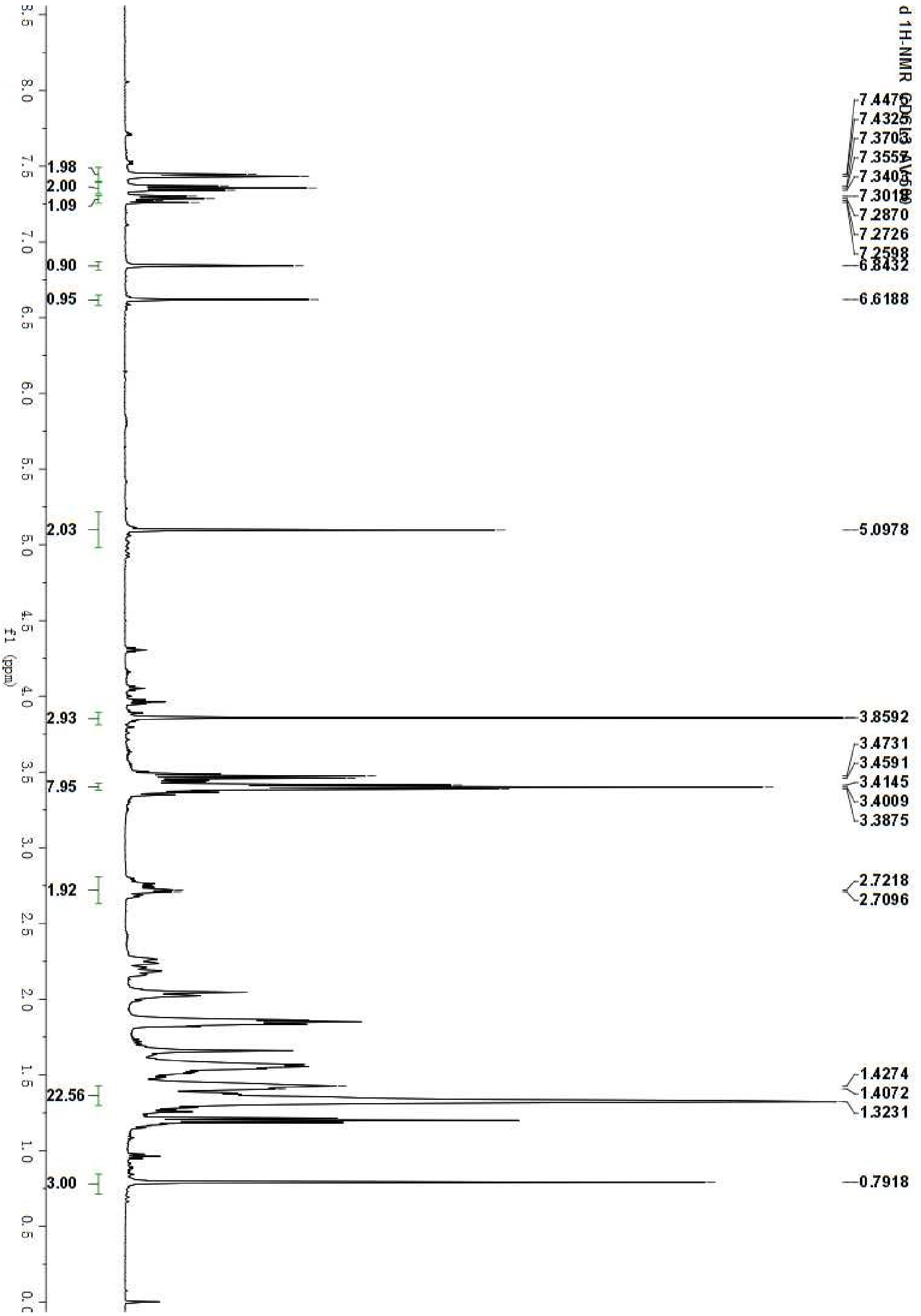

^{13}C NMR spectrum of **7**

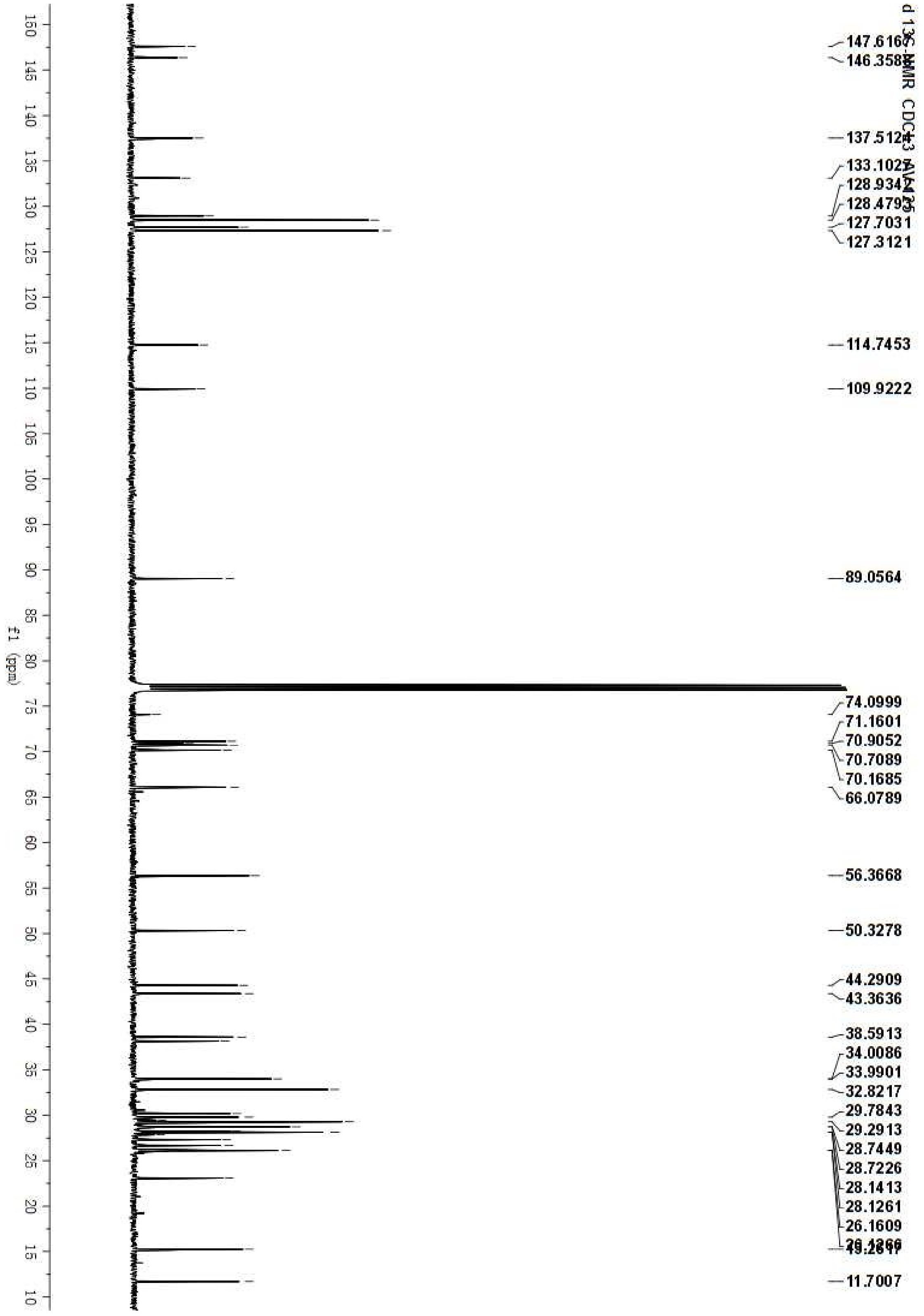

HRMS spectrum of **7**

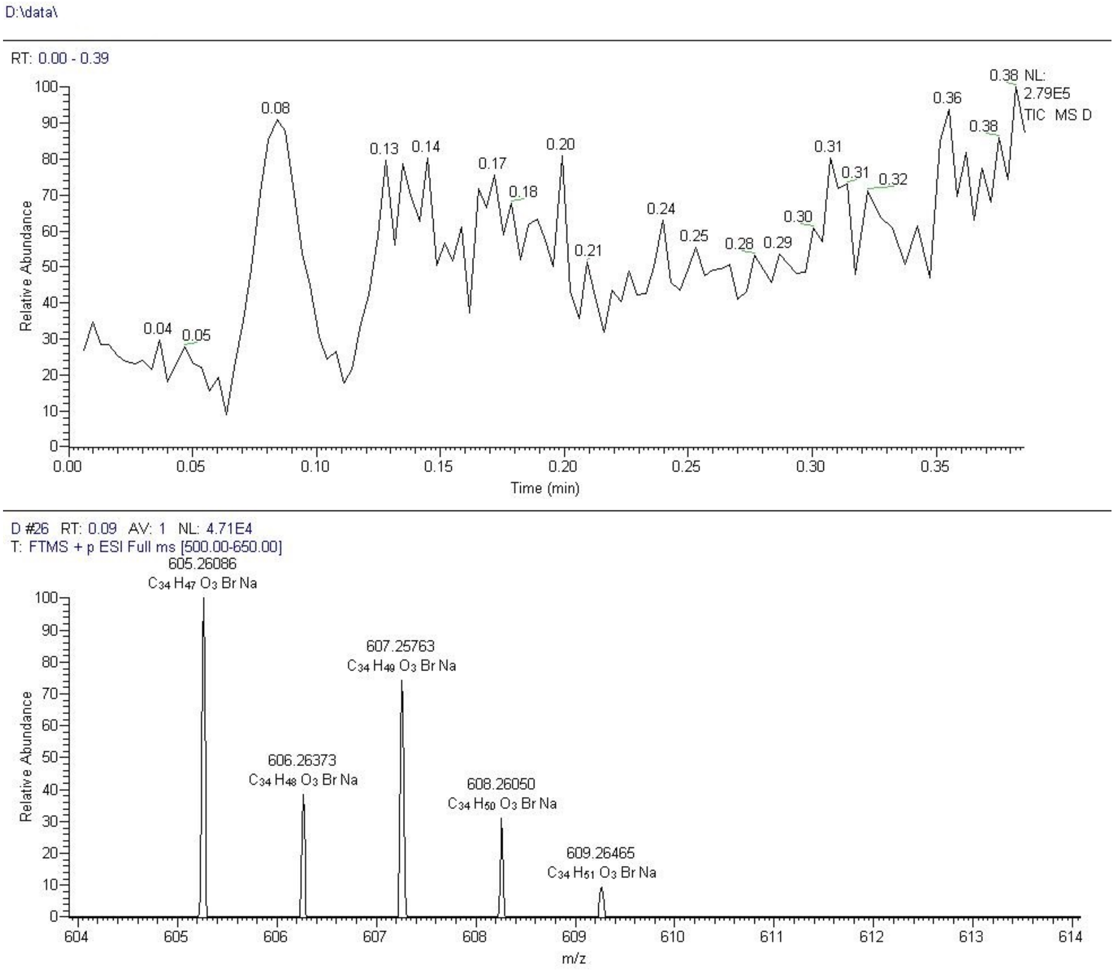

^{1}H NMR spectrum of **8**

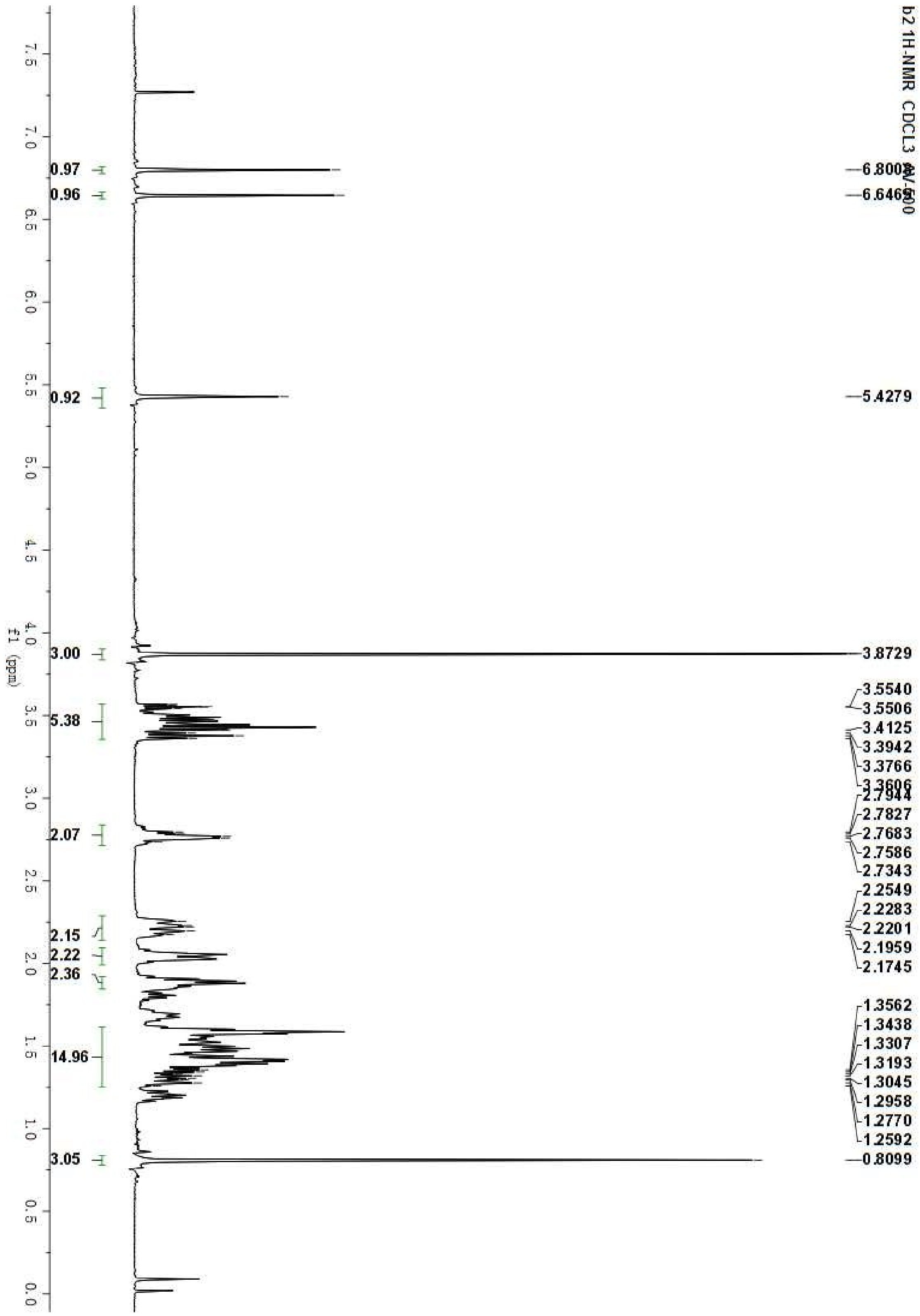

^{13}C NMR spectrum of **8**

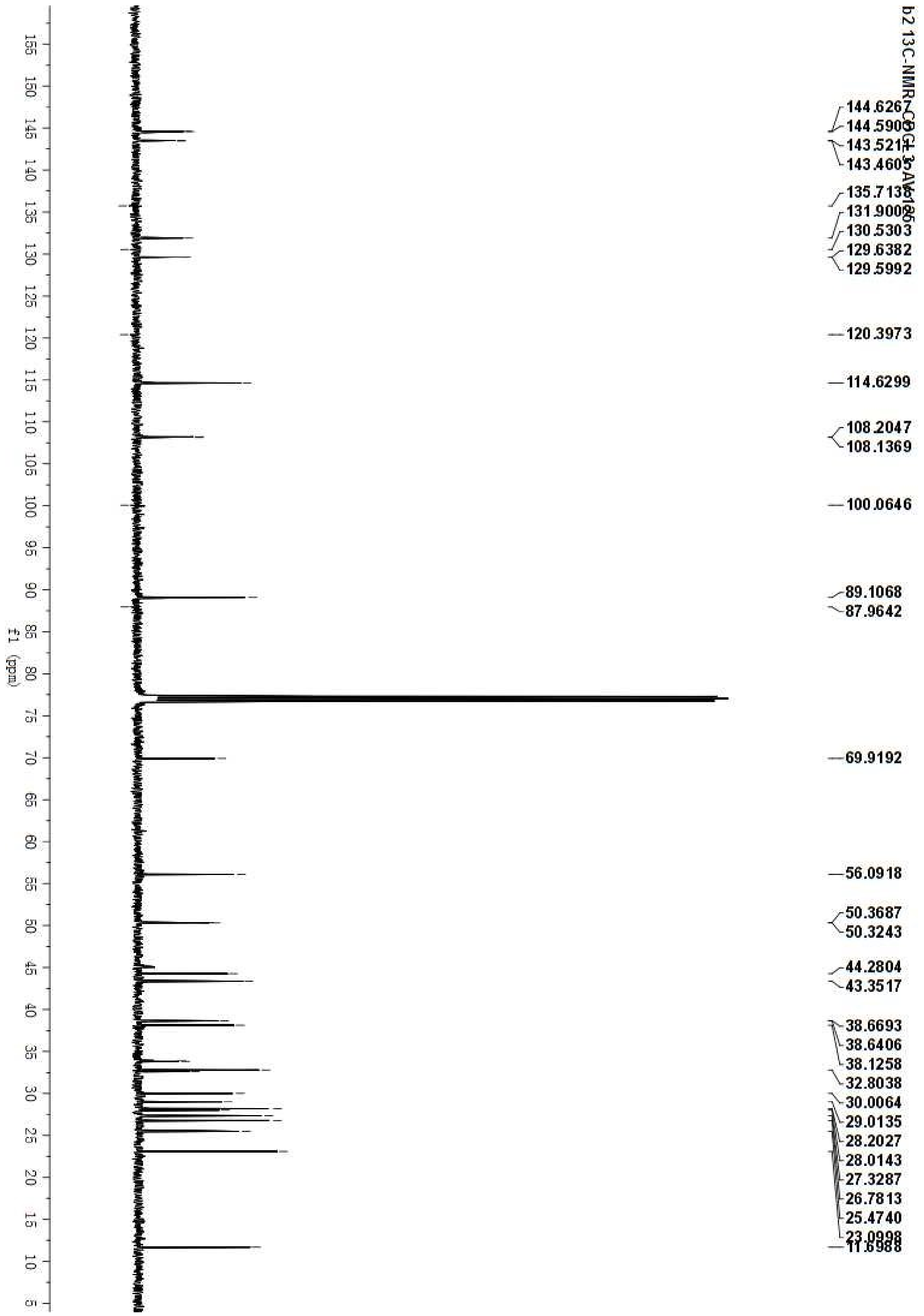

HRMS spectrum of **8**

**Sample Name**		**Position**	P2-A8	**Instrument Name**	Instrument 1	**User Name**	
**Inj Vol**	0.1	**InjPosition**		**SampleType**	Sample	**IRM Calibration Status**	Success
**Data Filename**	b2-p.d	**ACQ Method**		**Comment**		**Acquired Time**	

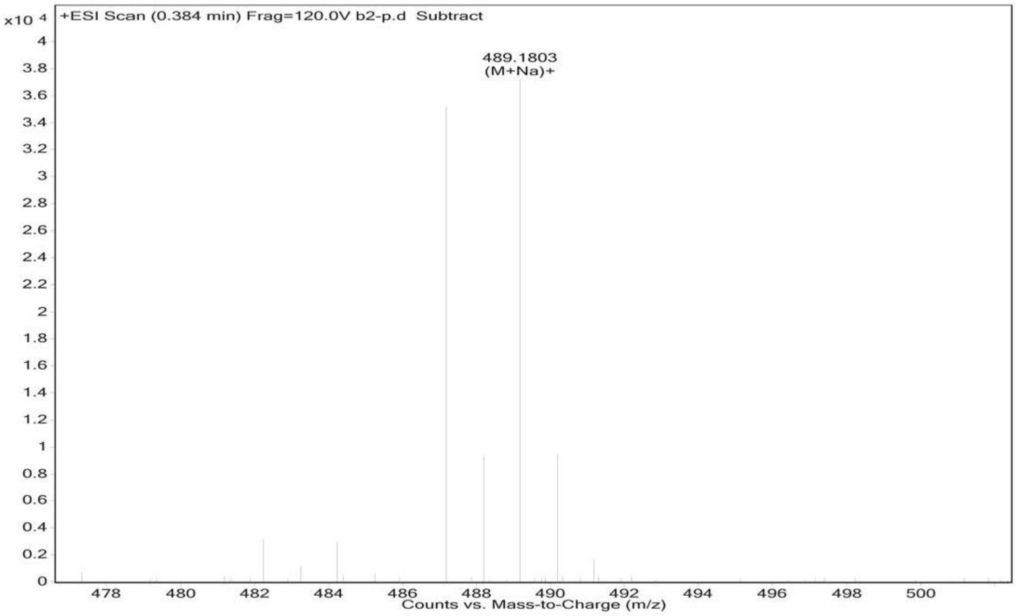

^{1}H NMR spectrum of **9**

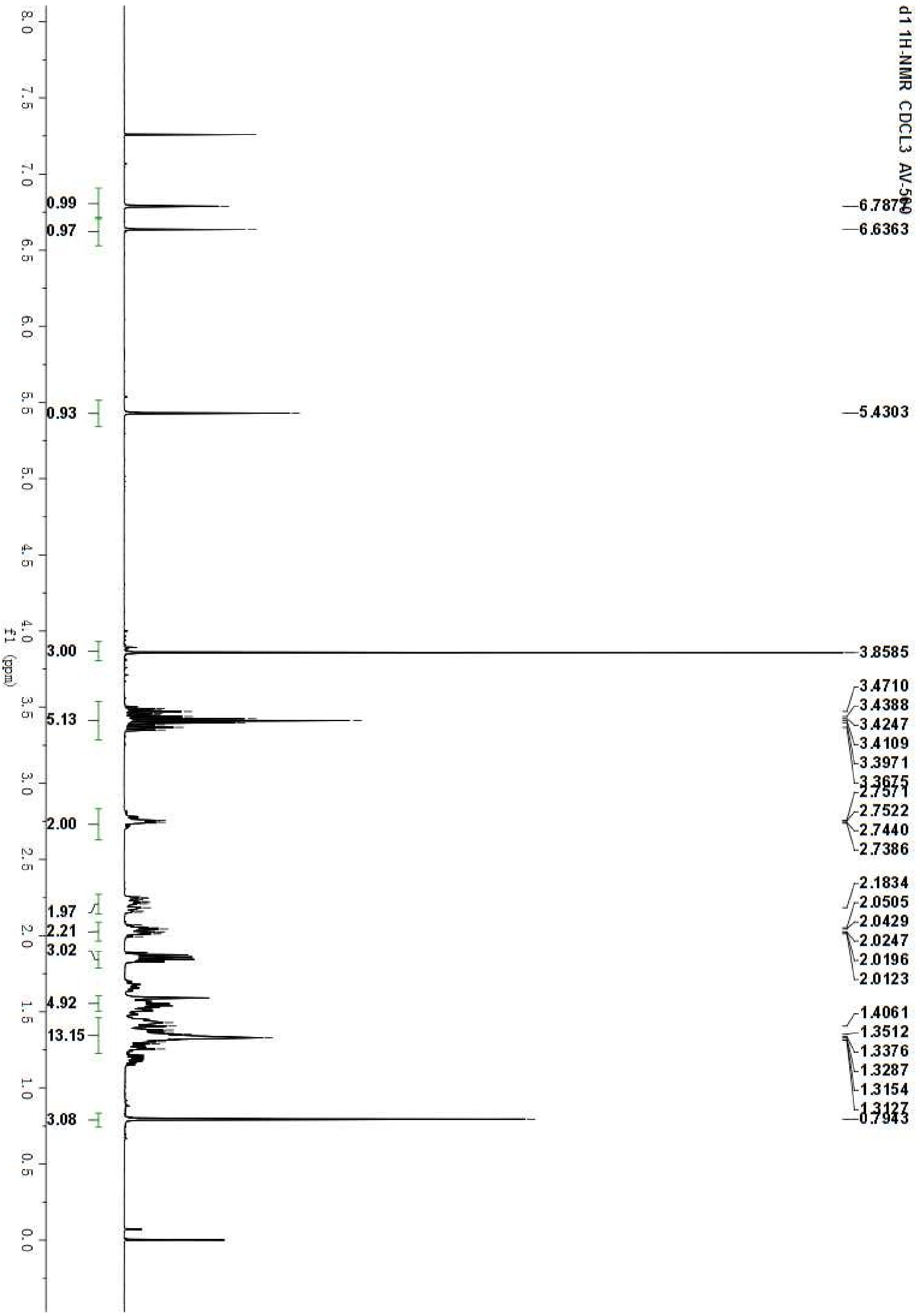

^{13}C NMR spectrum of **9**

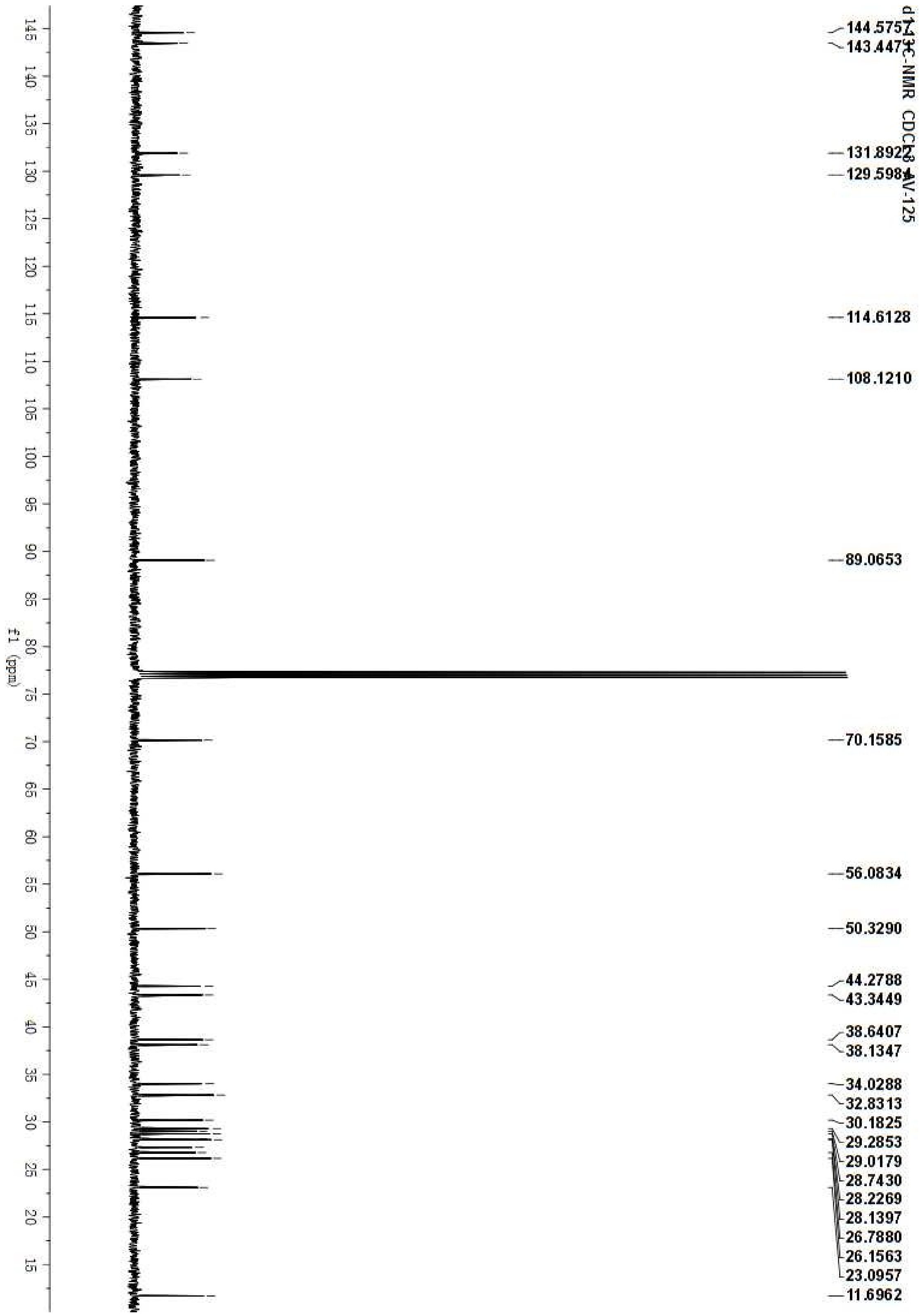

HRMS spectrum of **9**

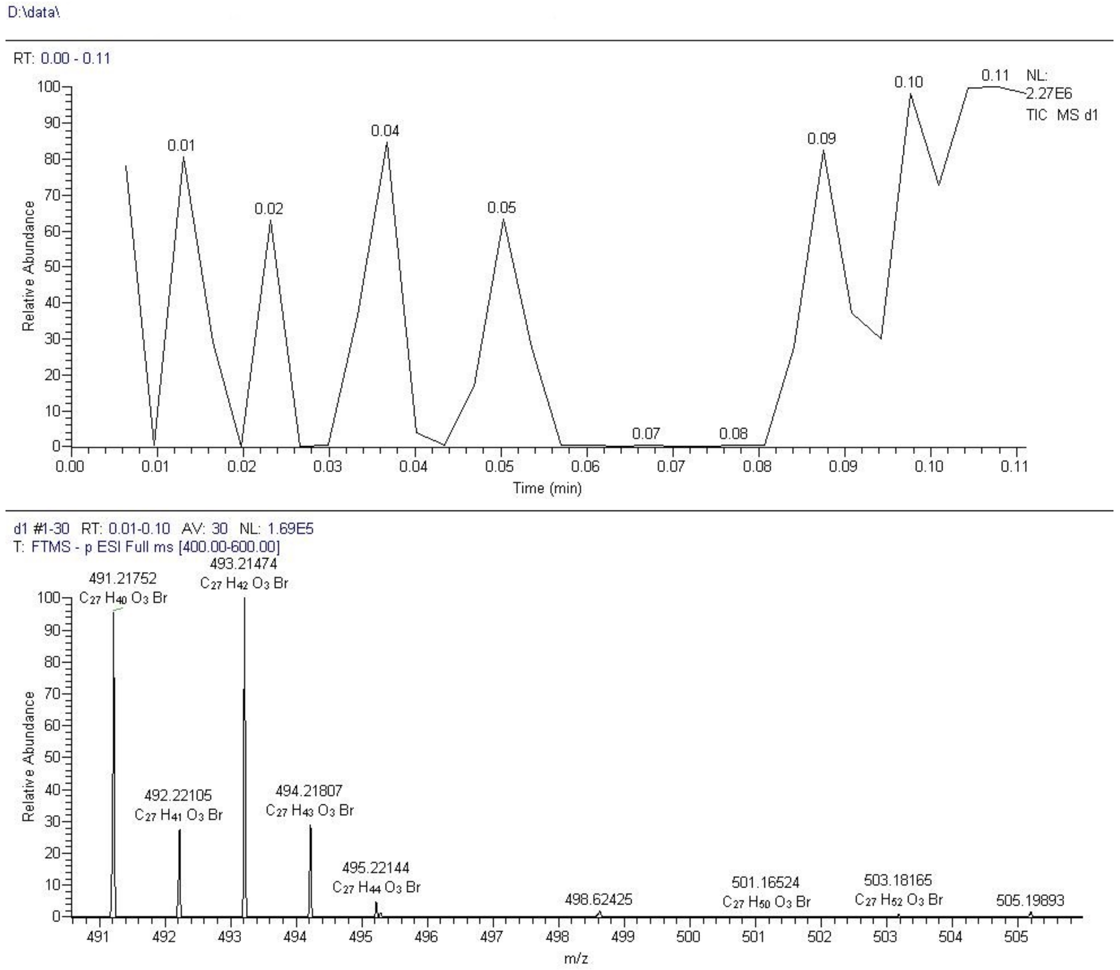

^{1}H NMR spectrum of **10**

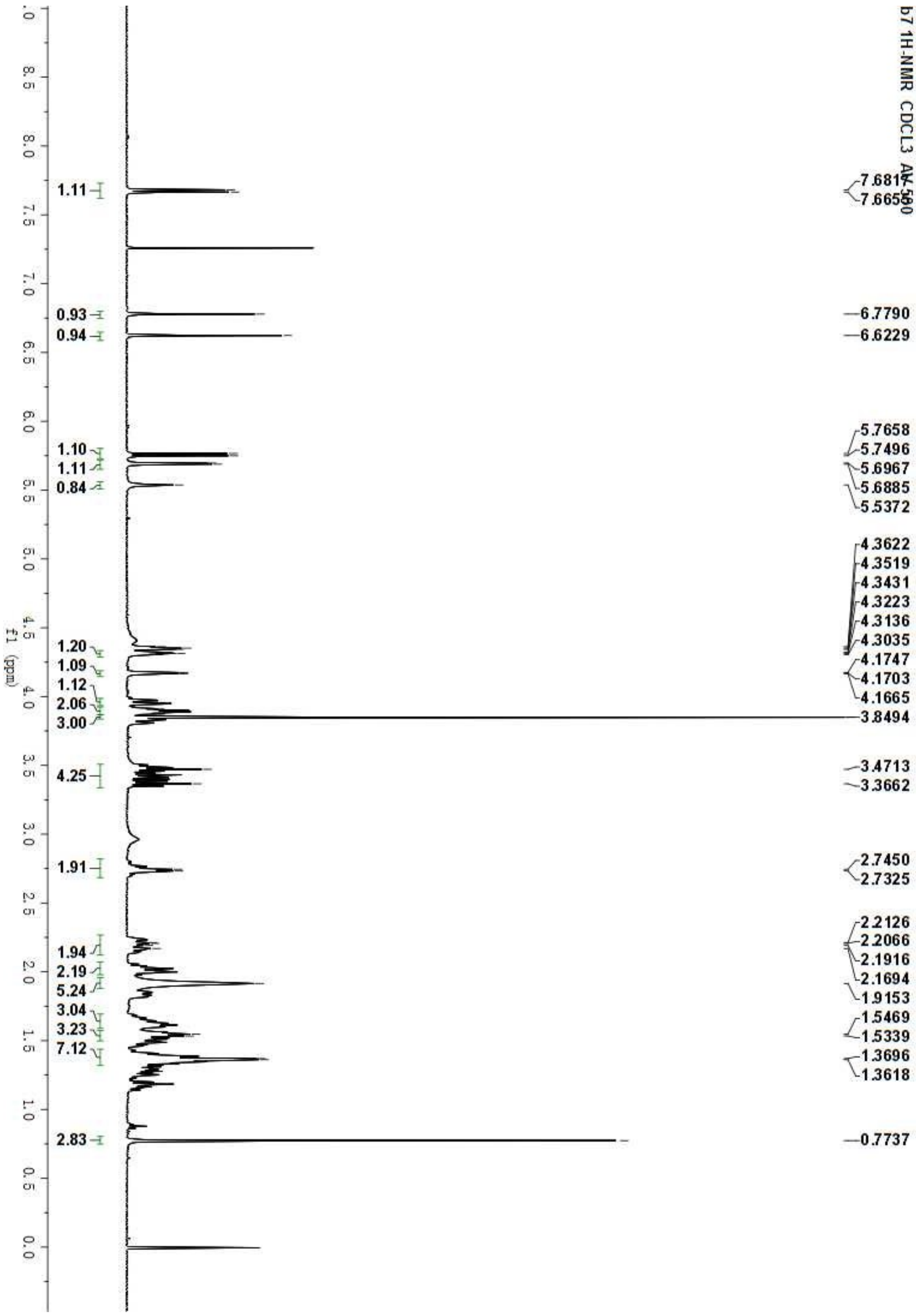

^{13}C NMR spectrum of **10**

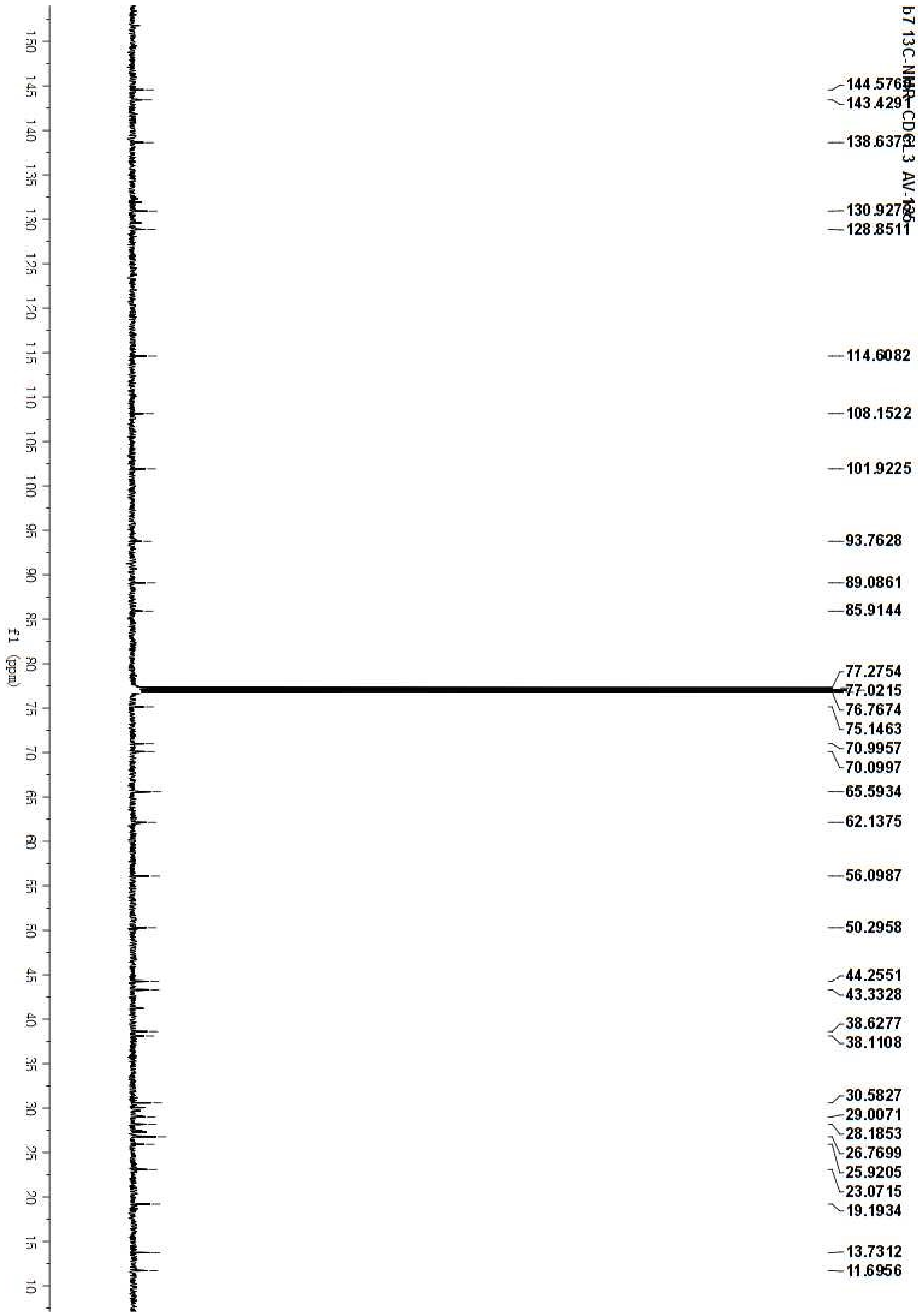

HRMS spectrum of **10**

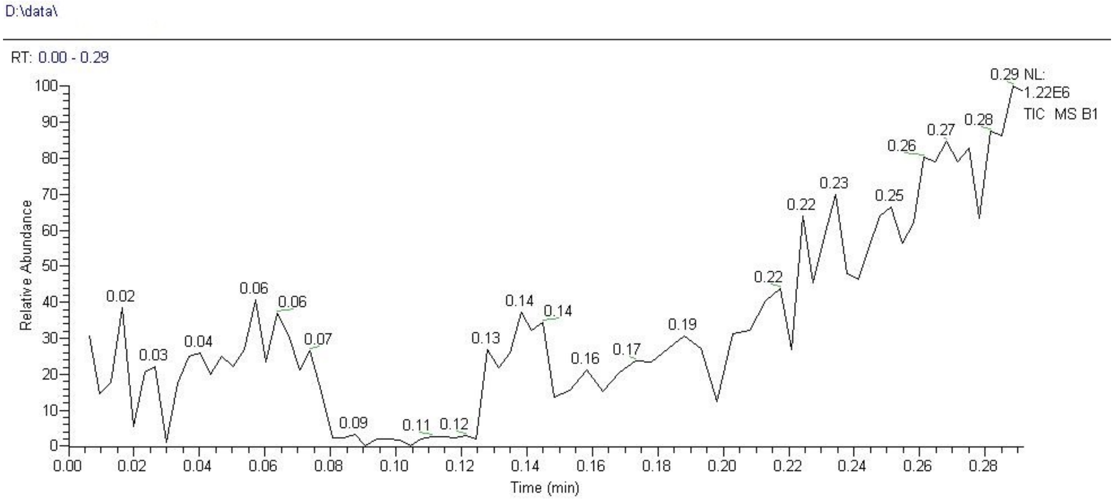

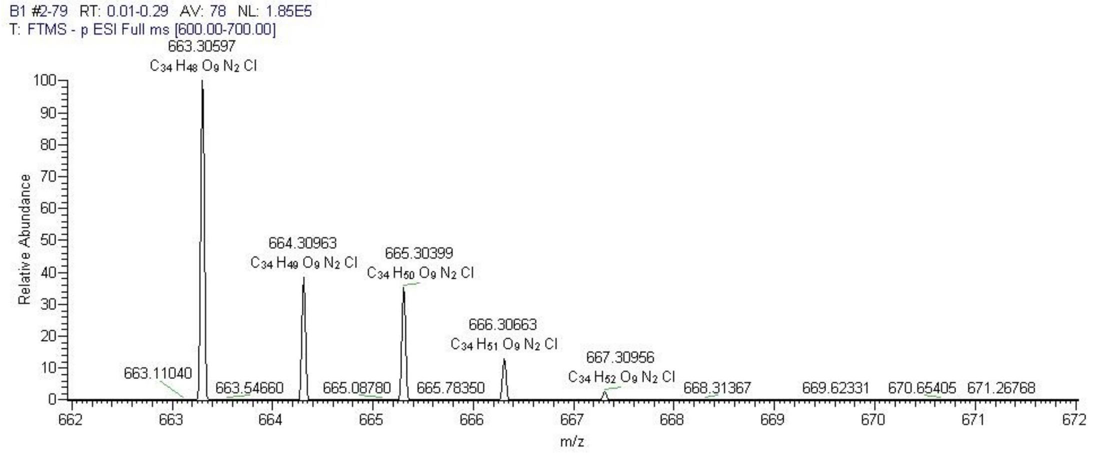

^{1}H NMR spectrum of **11**

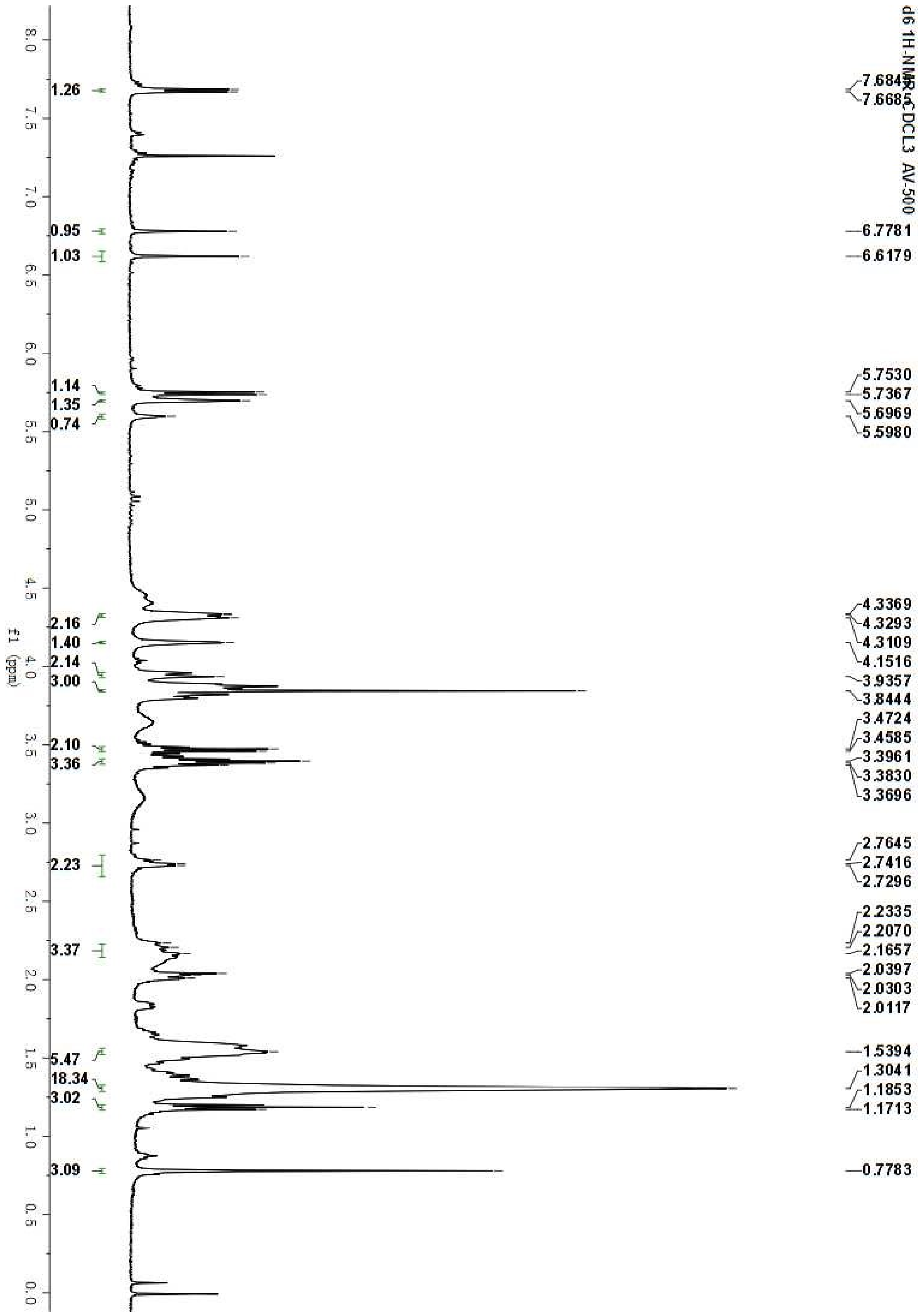

^{13}C NMR spectrum of **11**

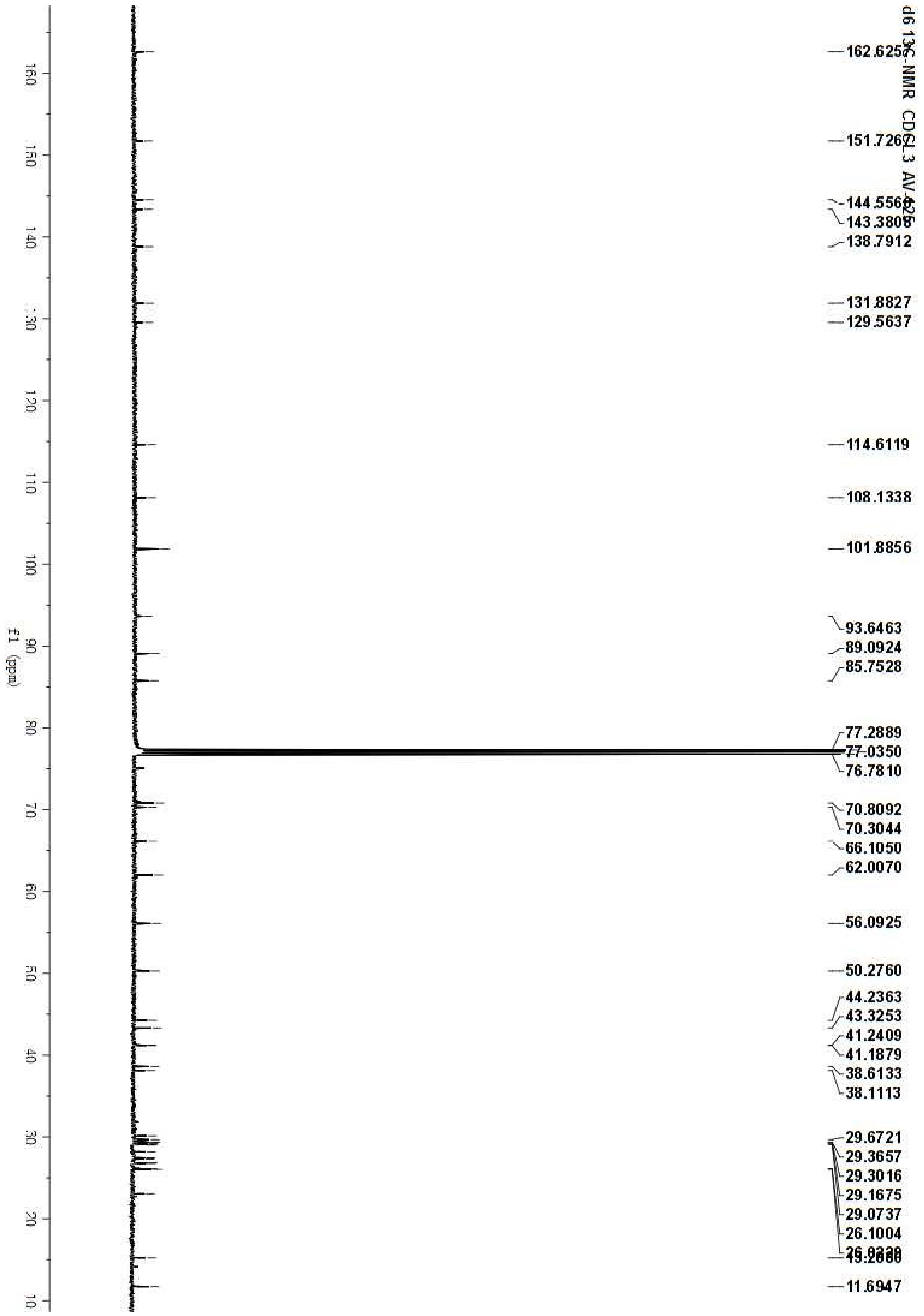

HRMS spectrum of **11**

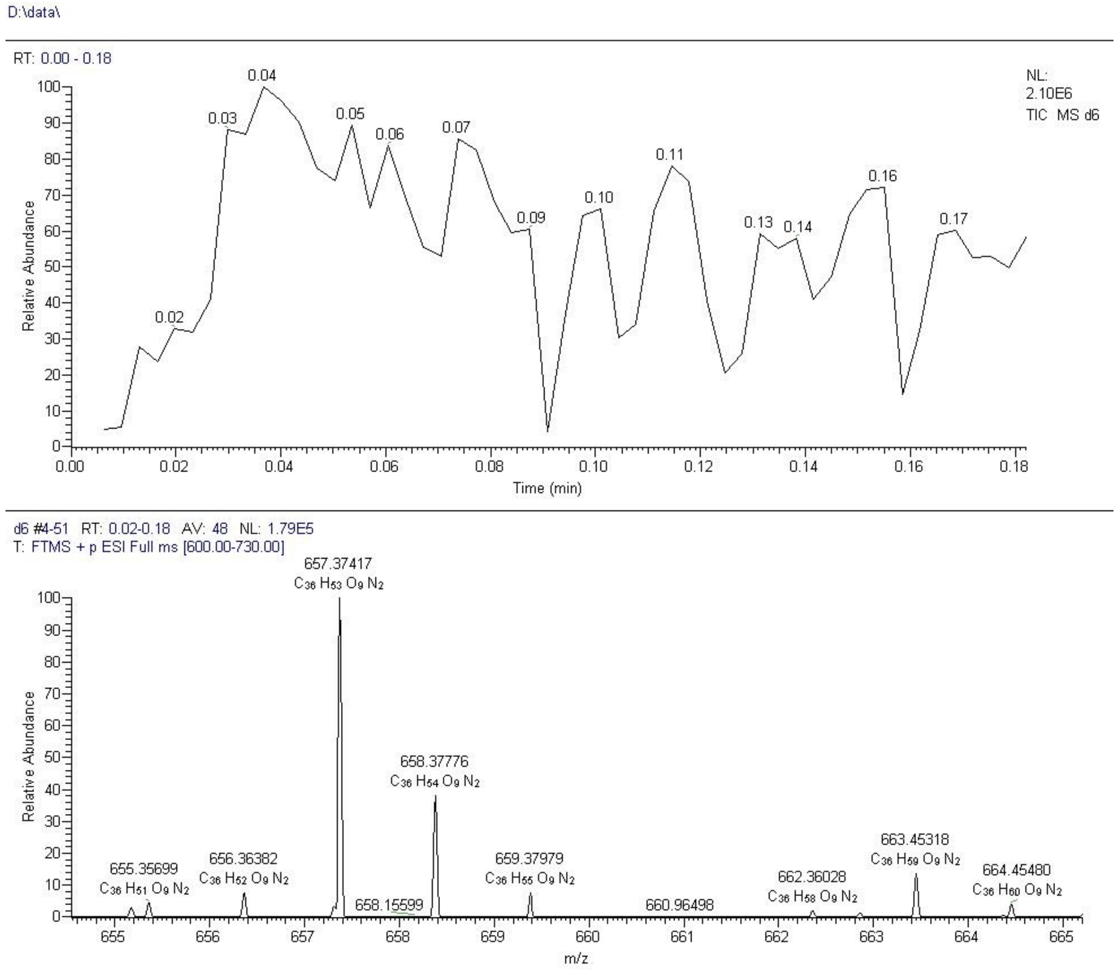

^{1}H NMR spectrum of **12a**

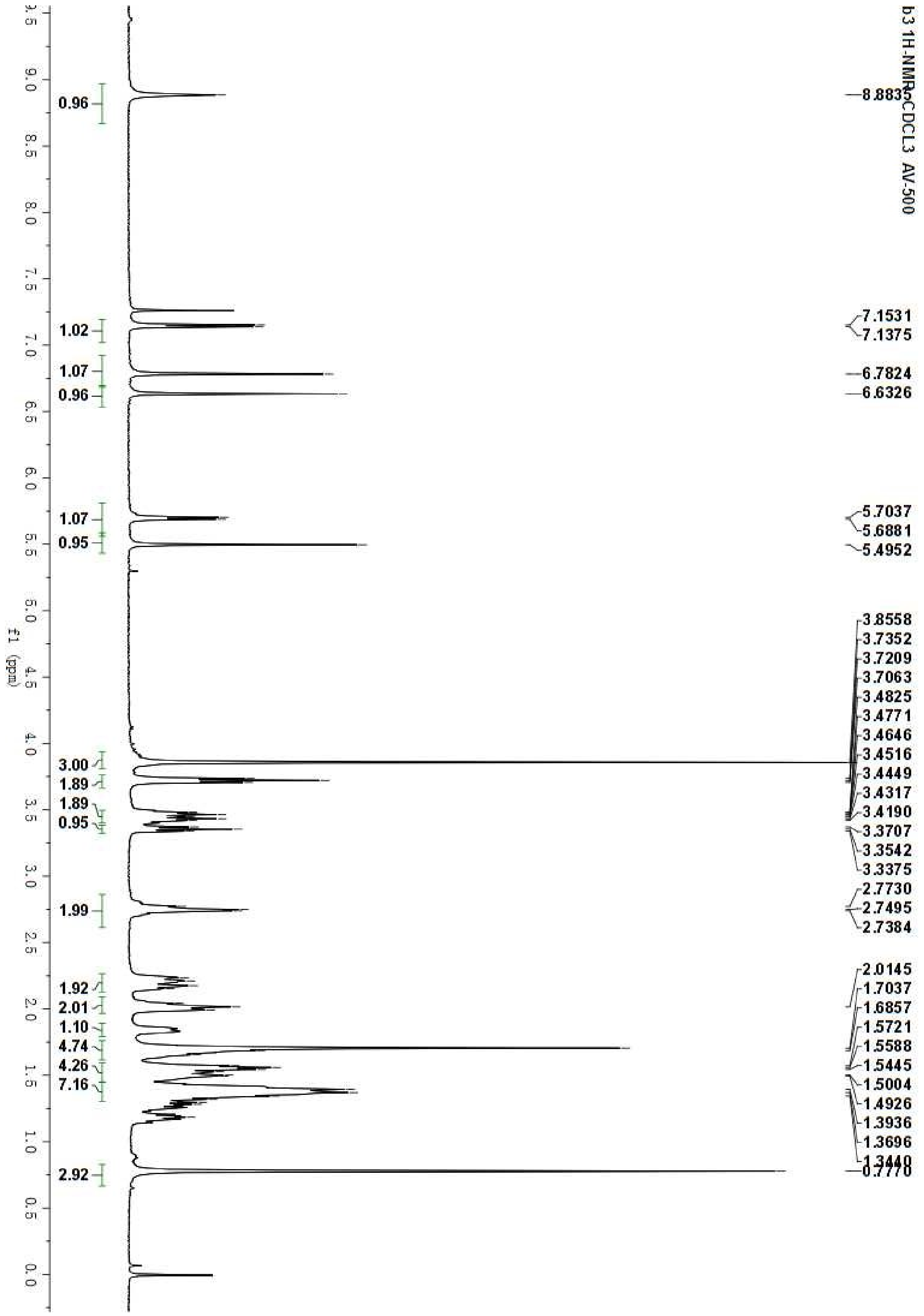

^{13}C NMR spectrum of **12a**

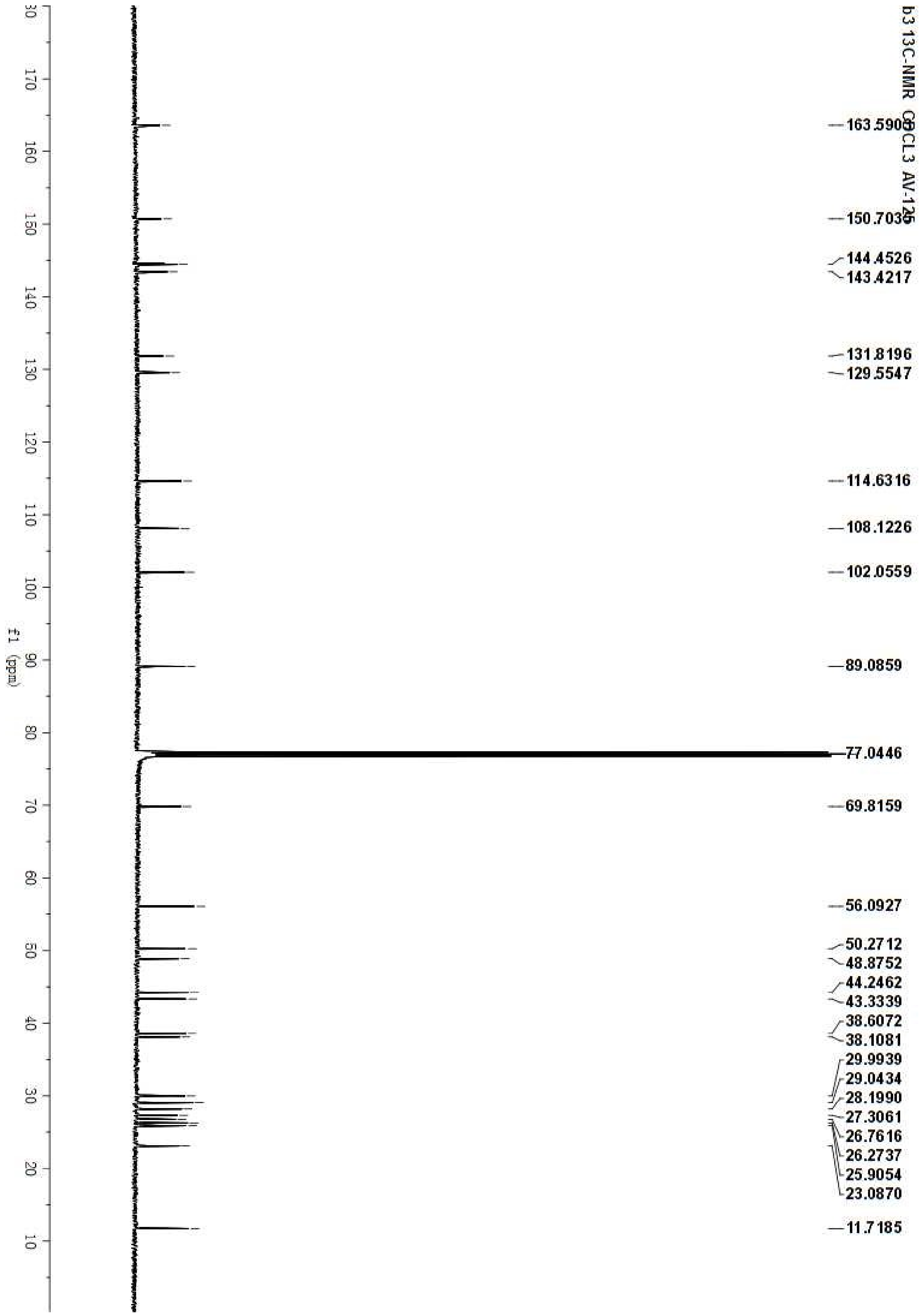

HRMS spectrum of **12a**

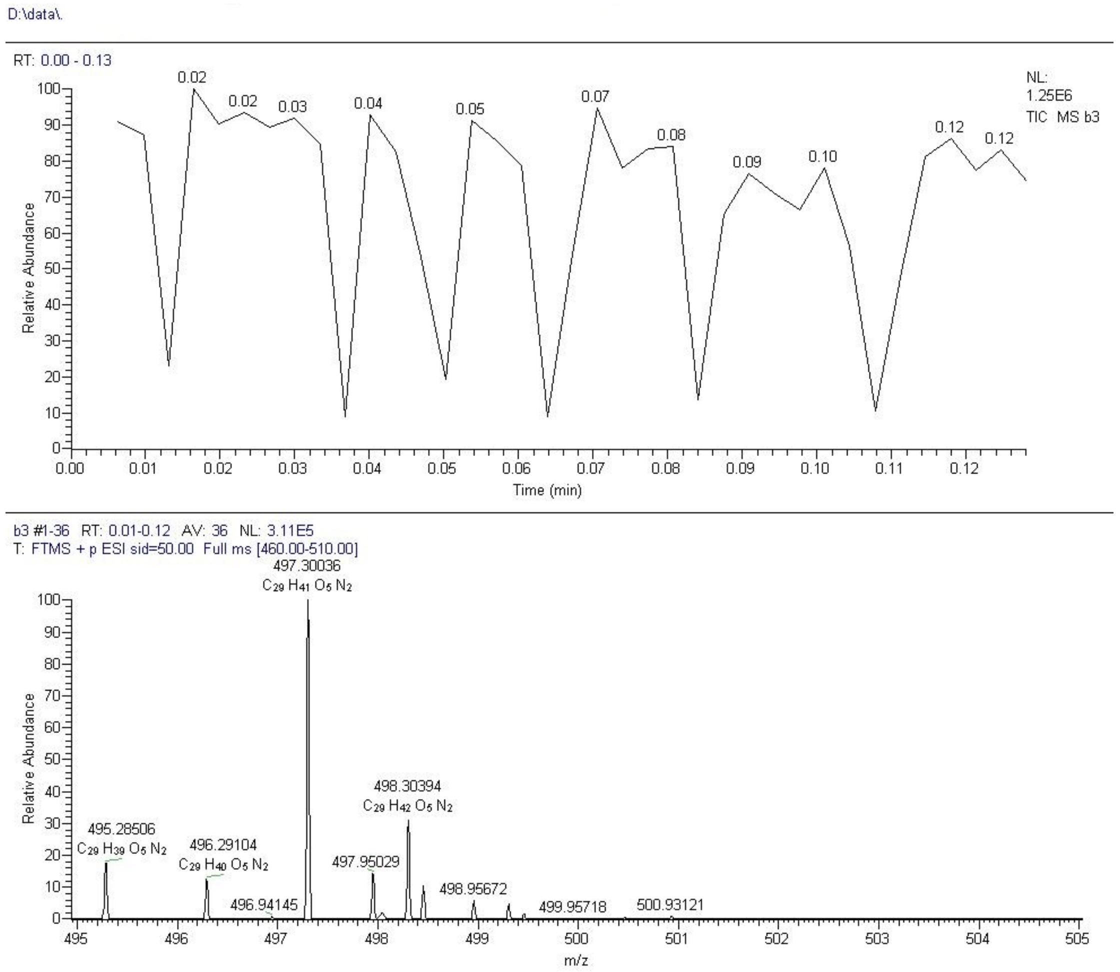

^{1}H NMR spectrum of **12b**

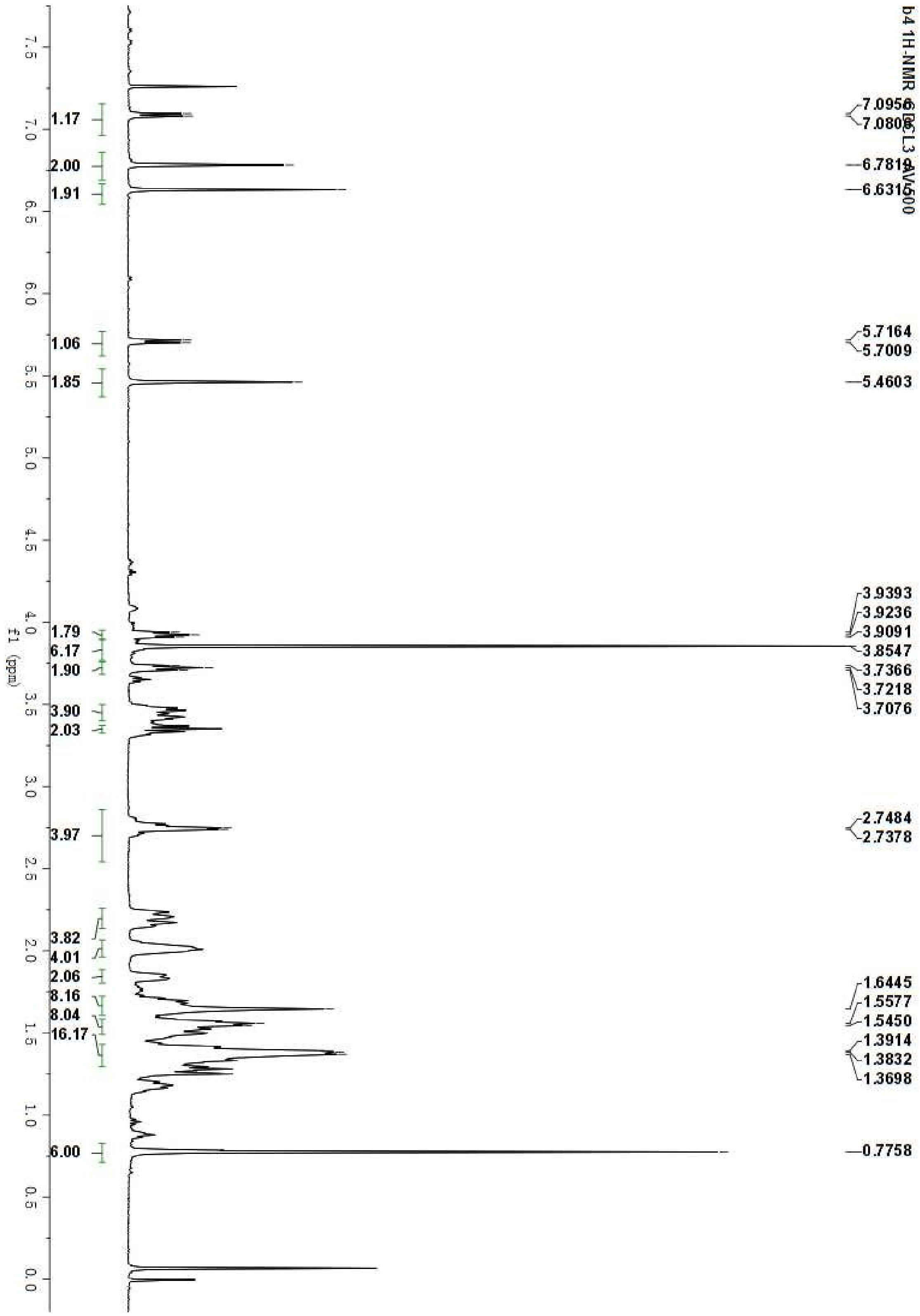

^{13}C NMR spectrum of **12b**

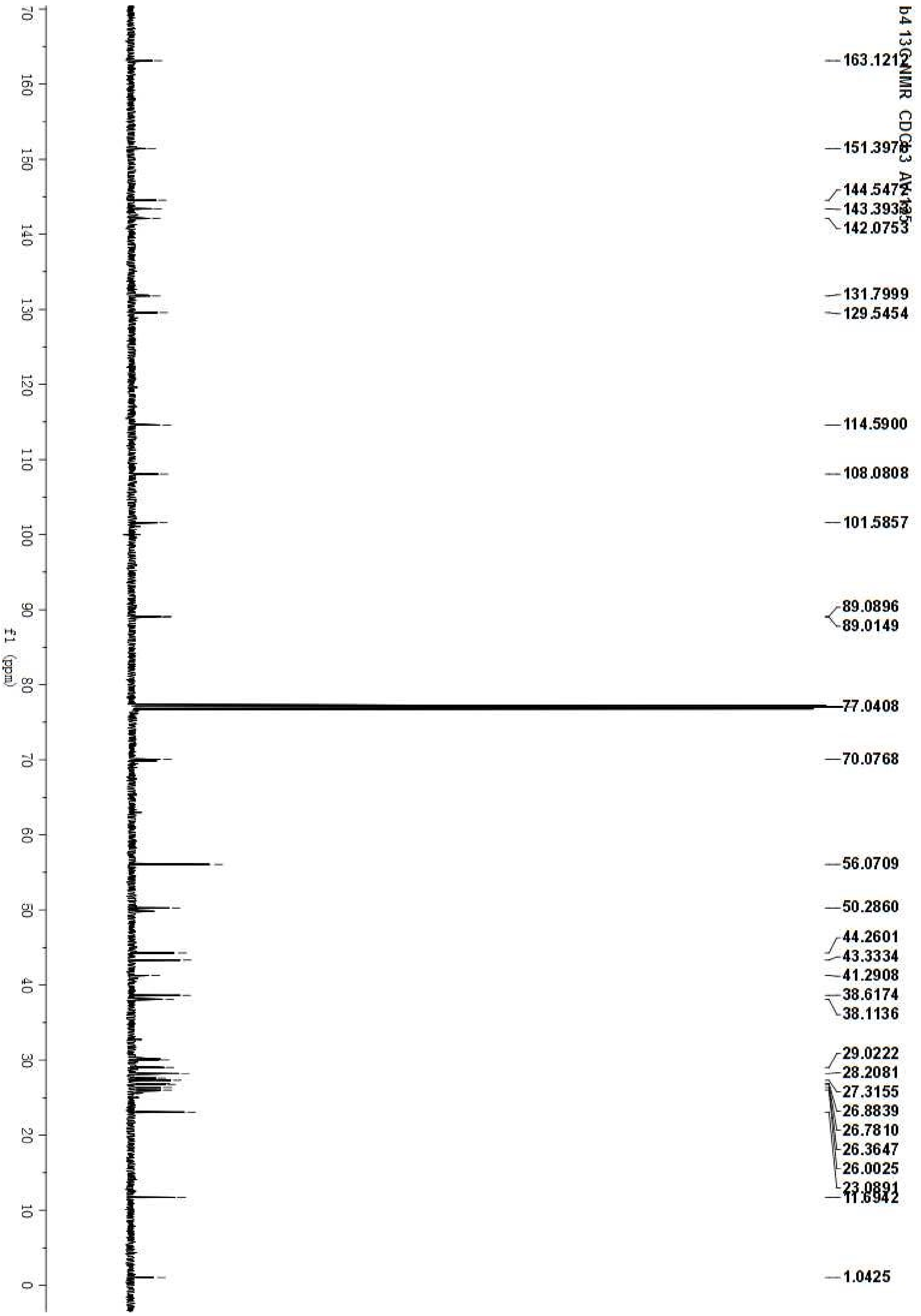

HRMS spectrum of **12b**

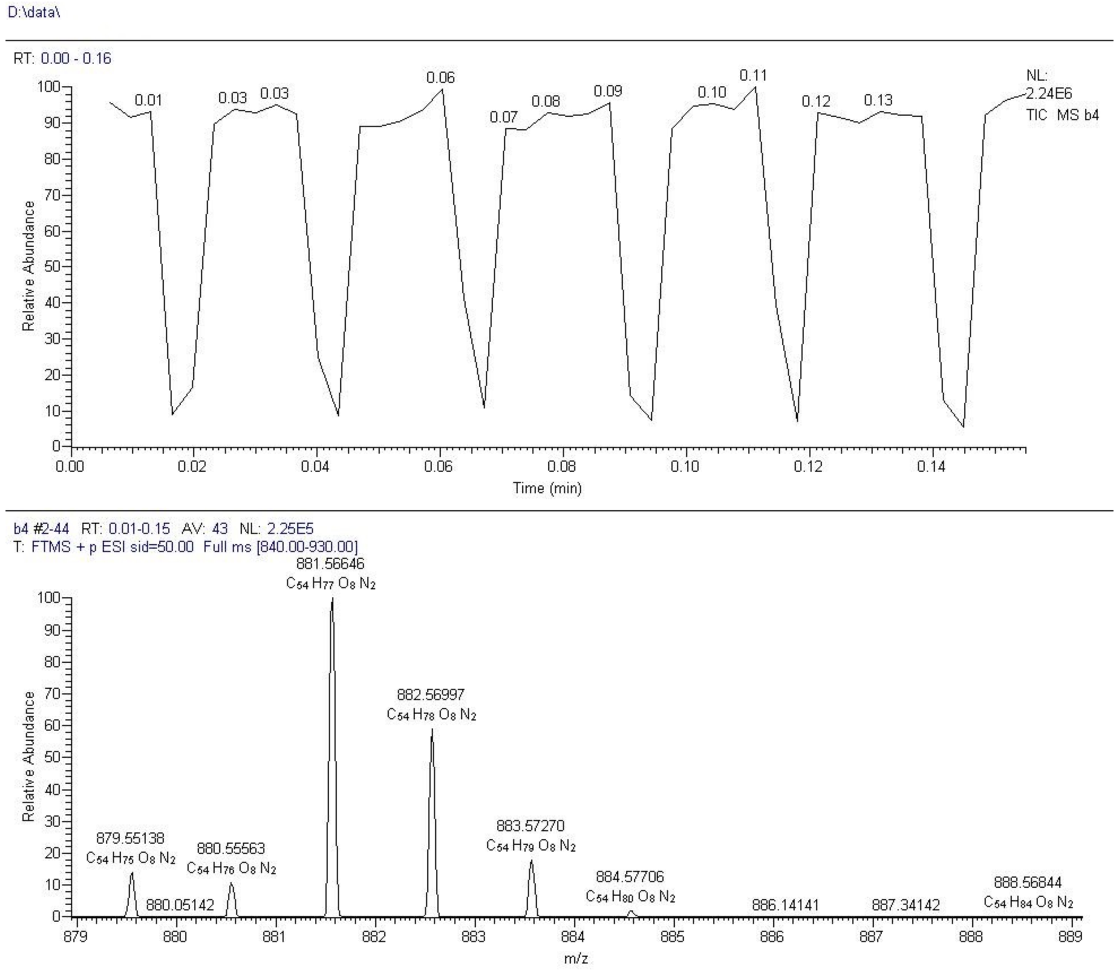

^{1}H NMR spectrum of **13a**

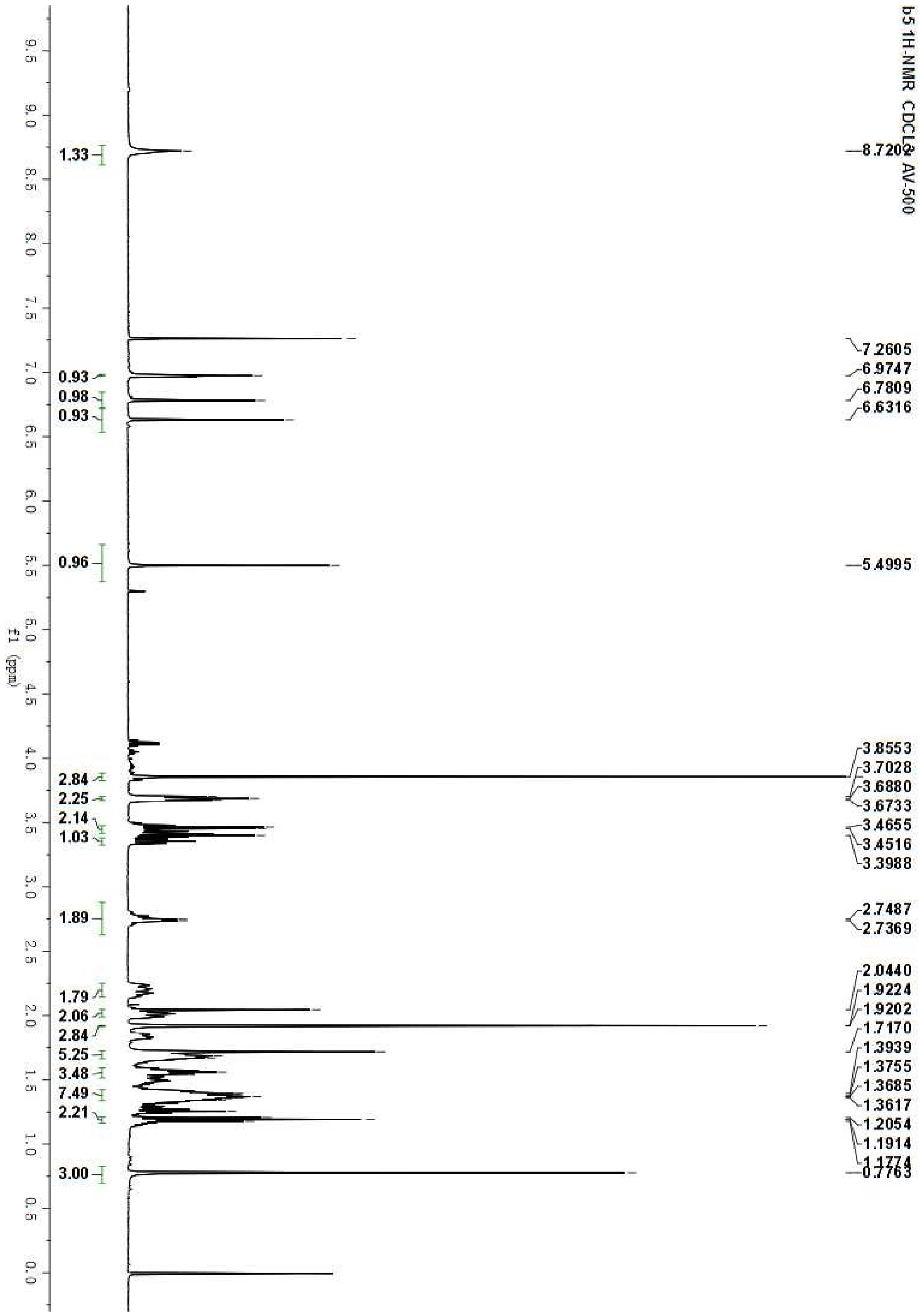

^{13}C NMR spectrum of **13a**

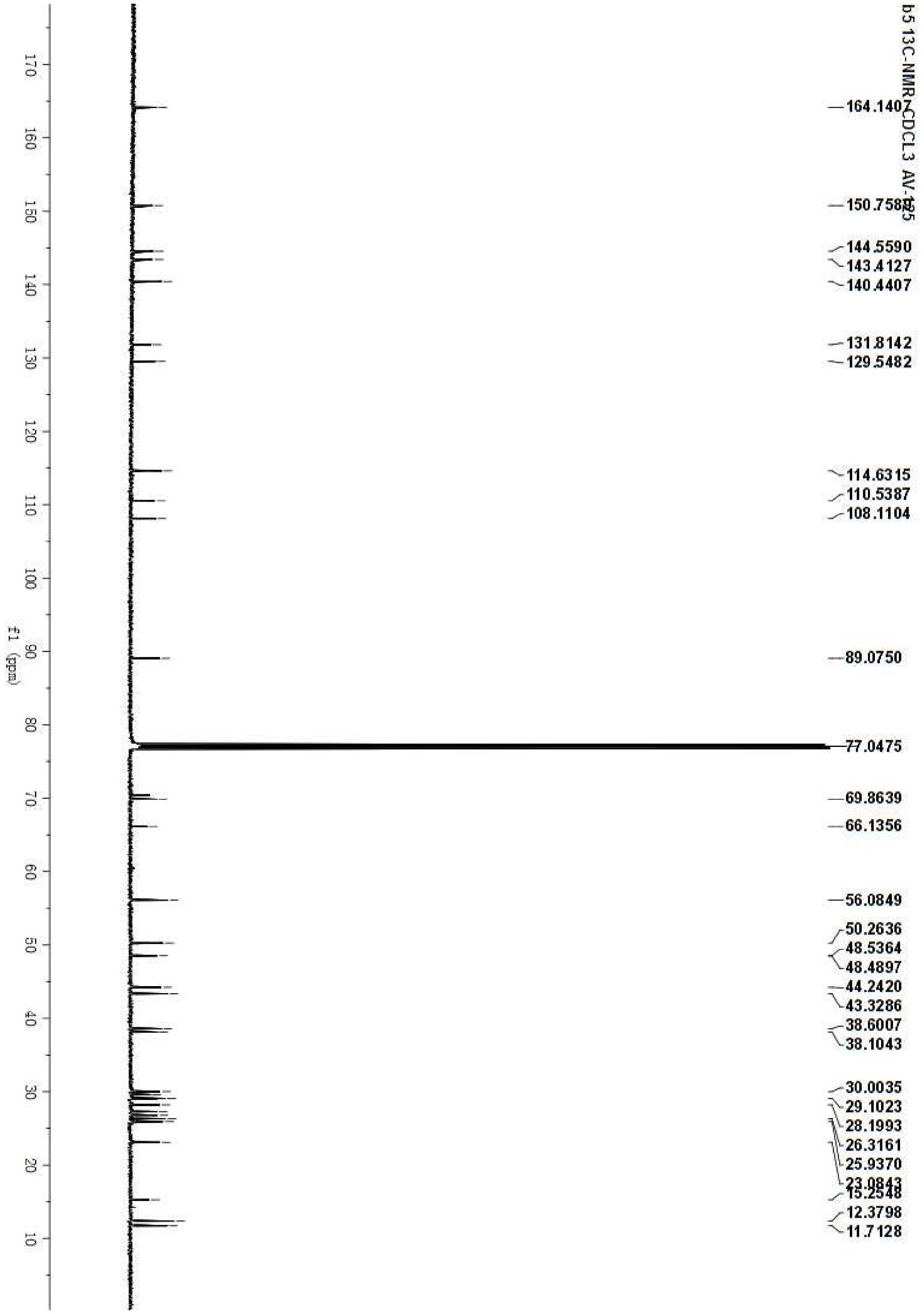

HRMS spectrum of **13a**

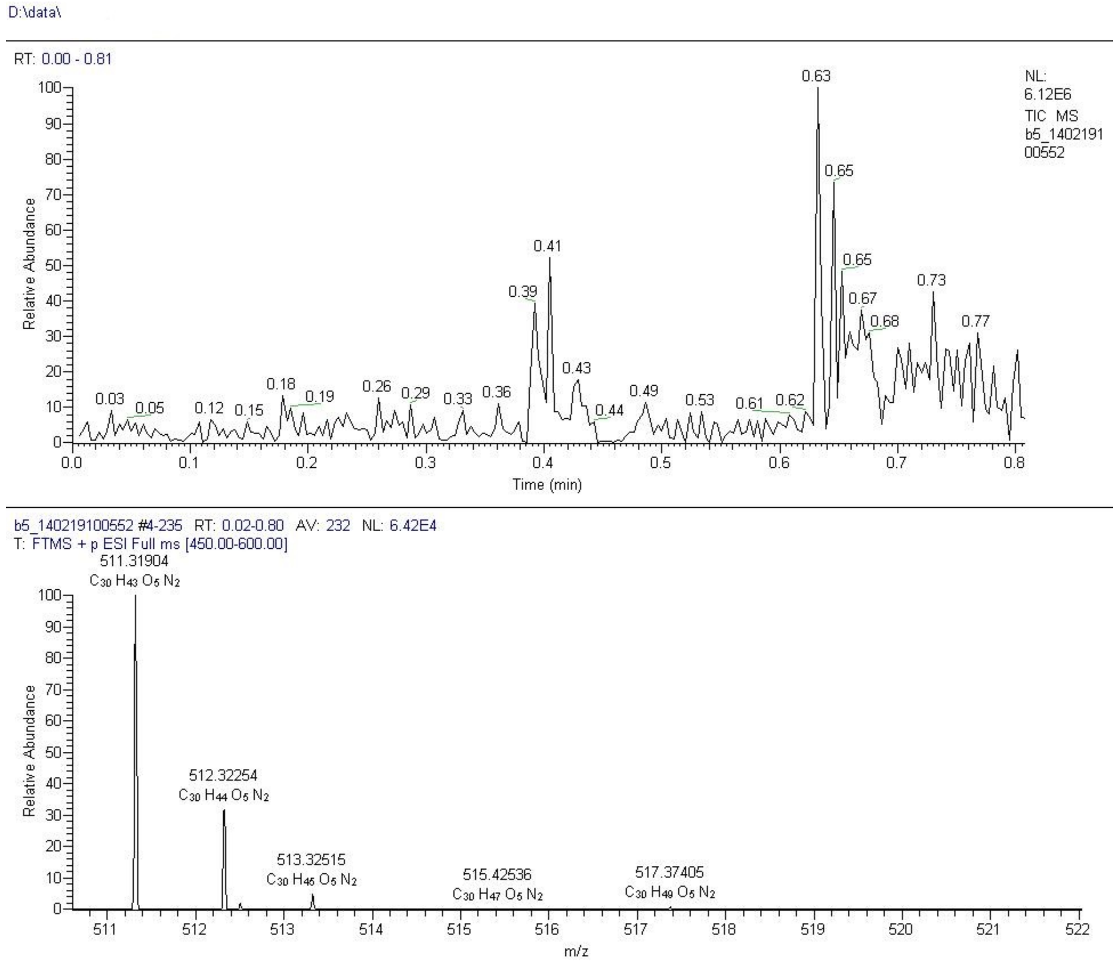

^{1}H NMR spectrum of **13b**

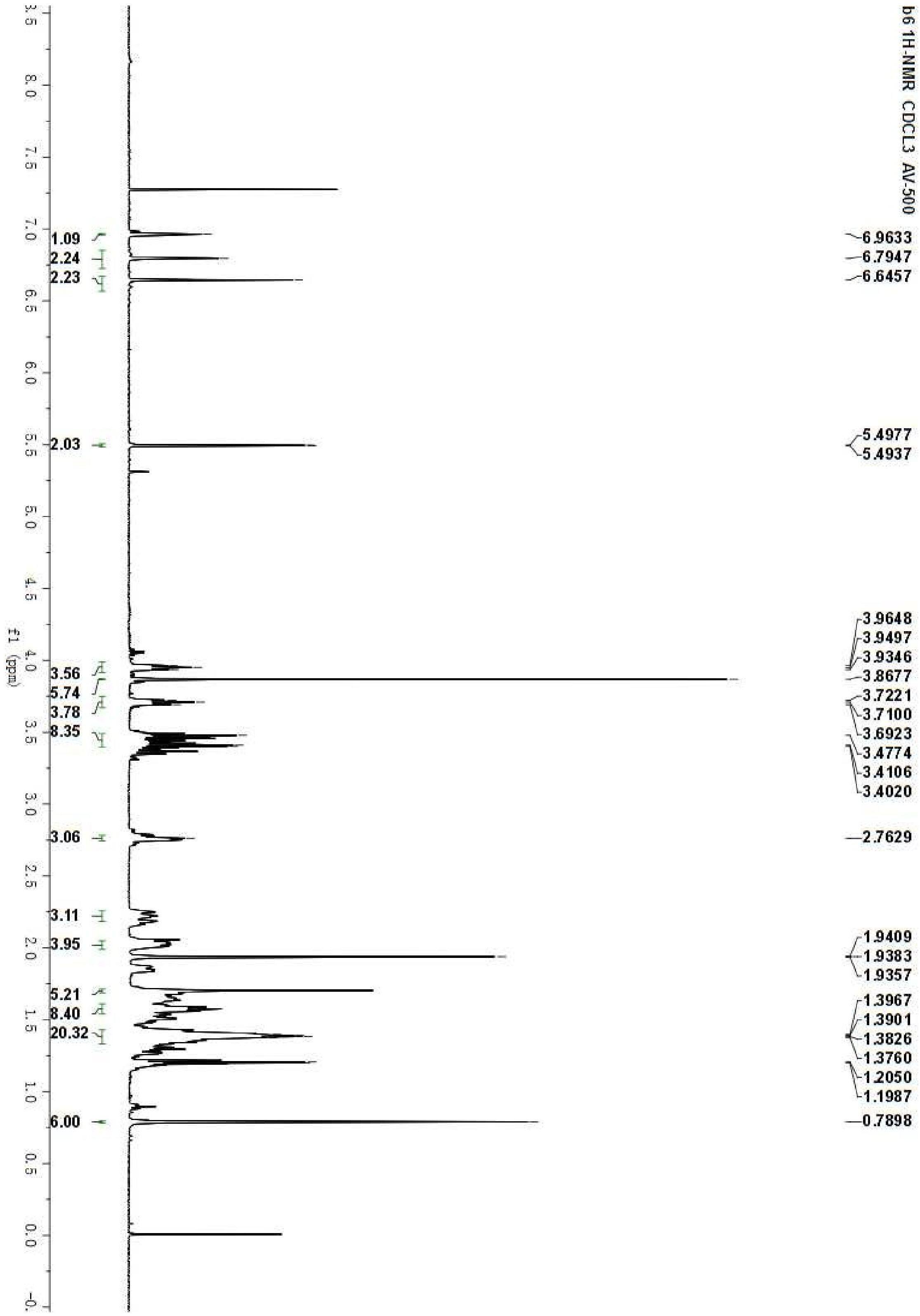

^{13}C NMR spectrum of **13b**

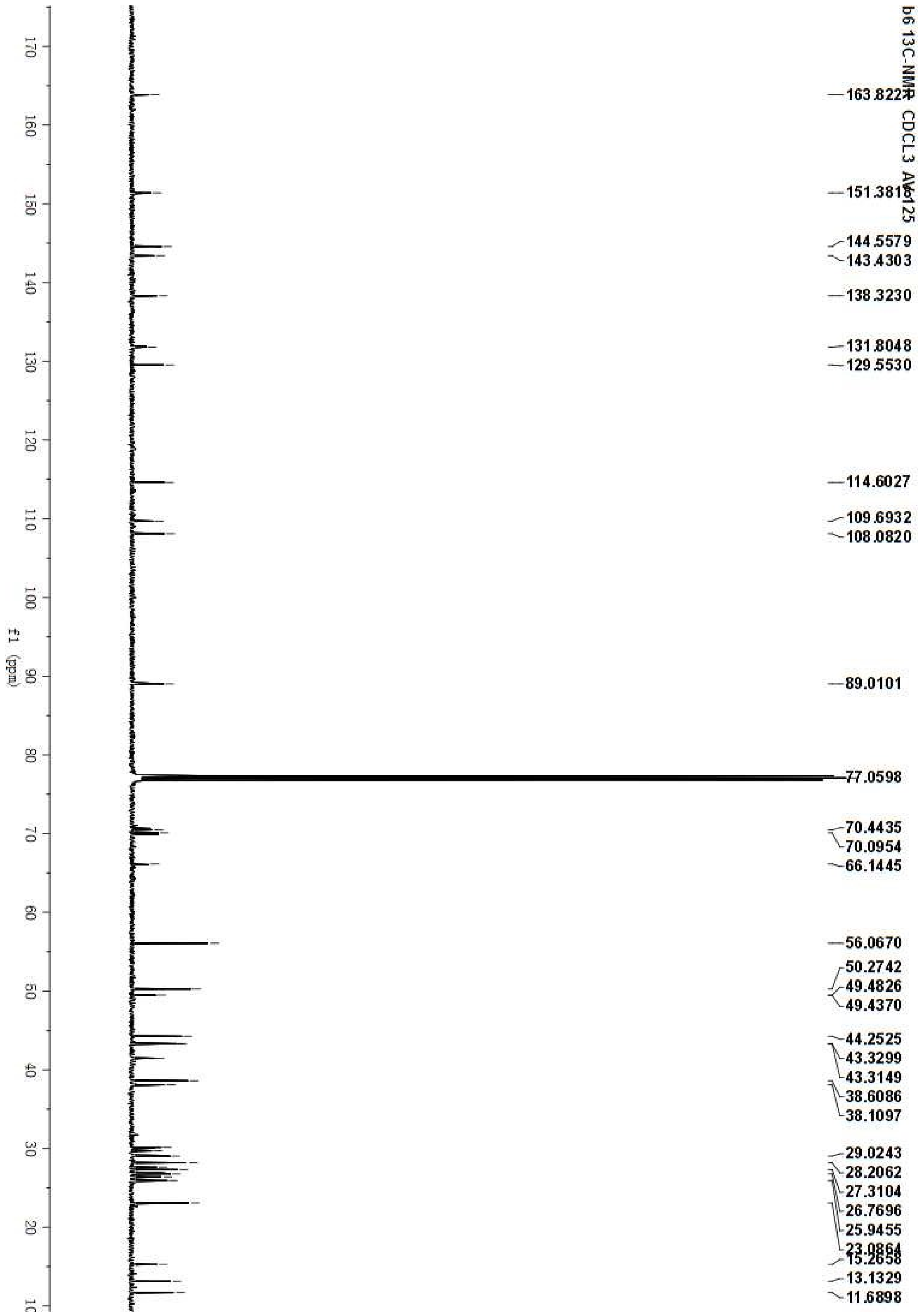

HRMS spectrum of **13b**

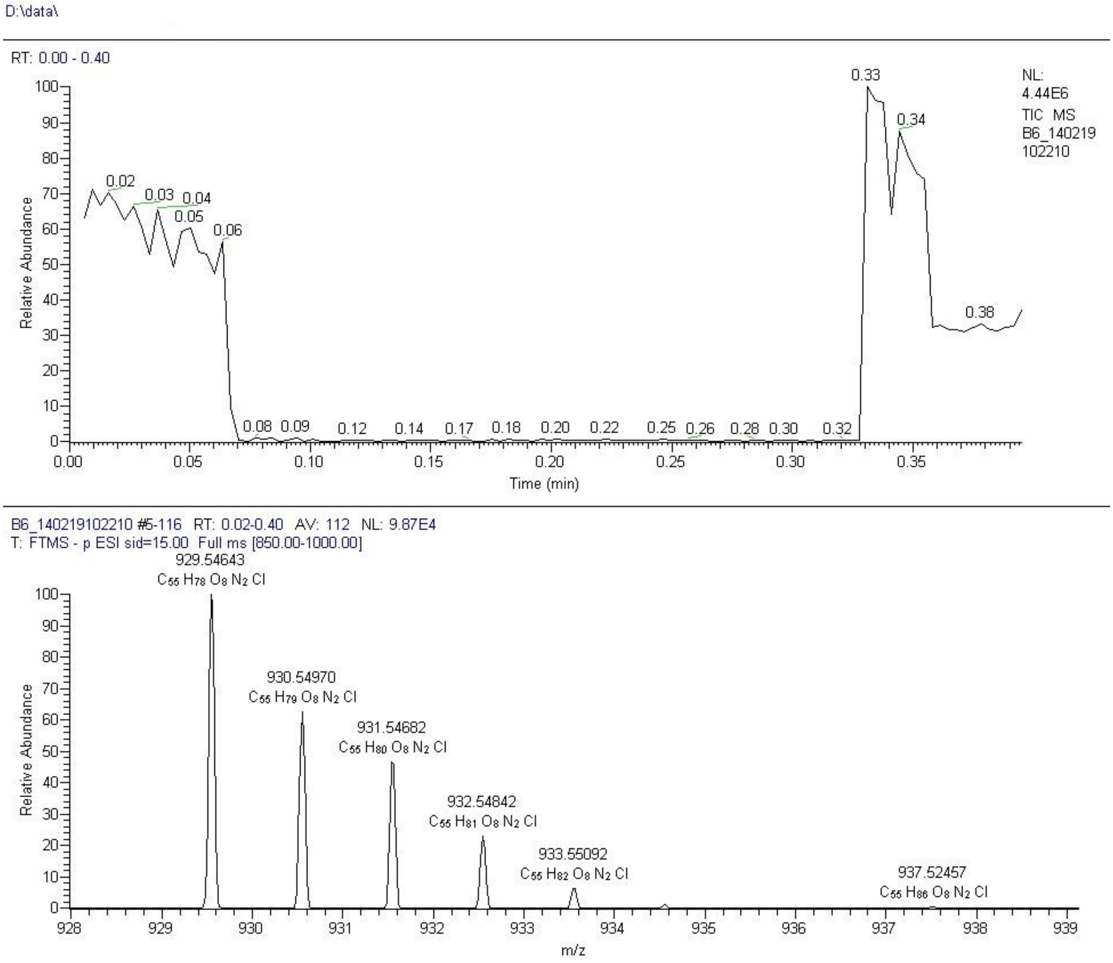

^{1}H NMR spectrum of **14a**

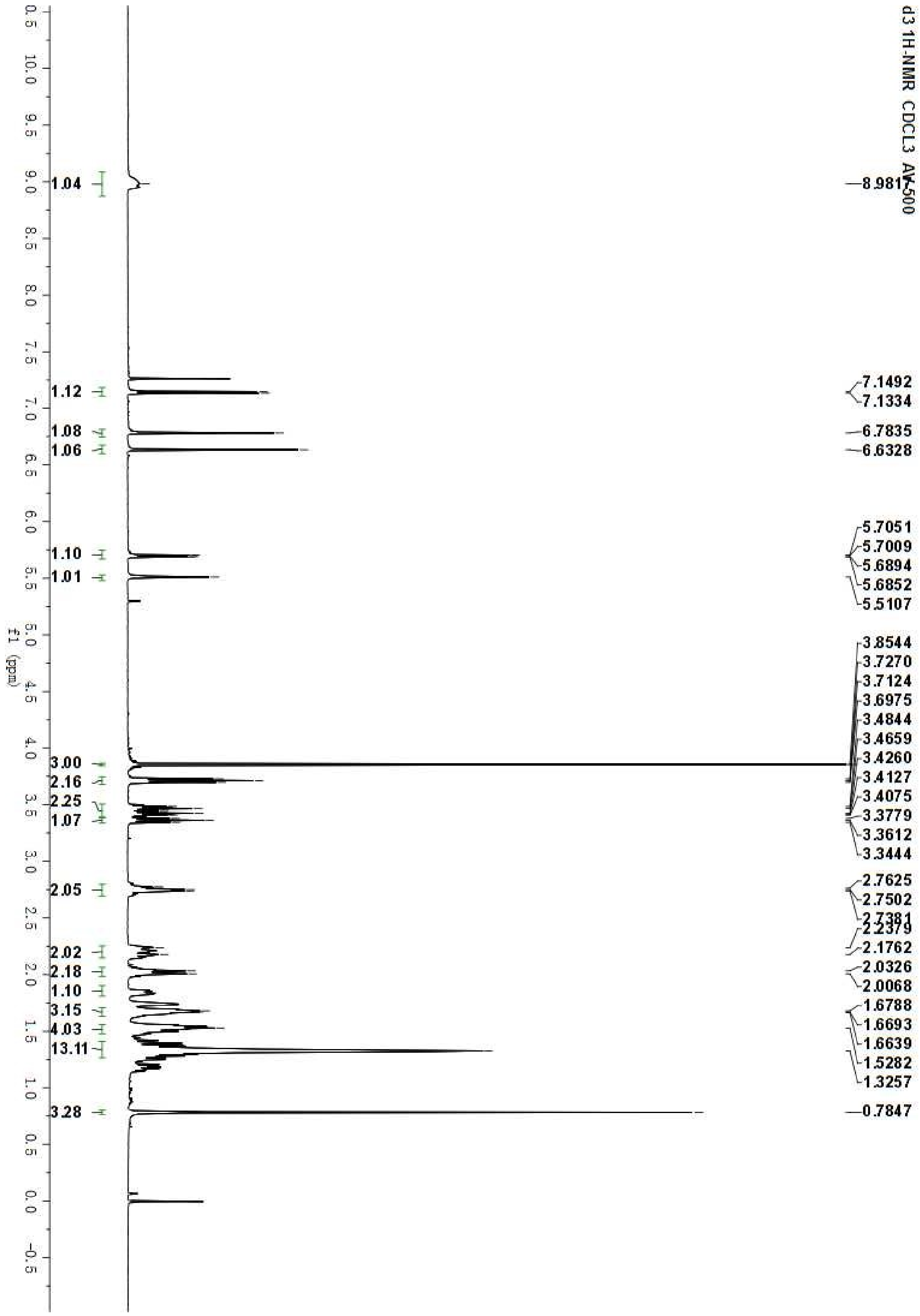

^{13}C NMR spectrum of **14a**

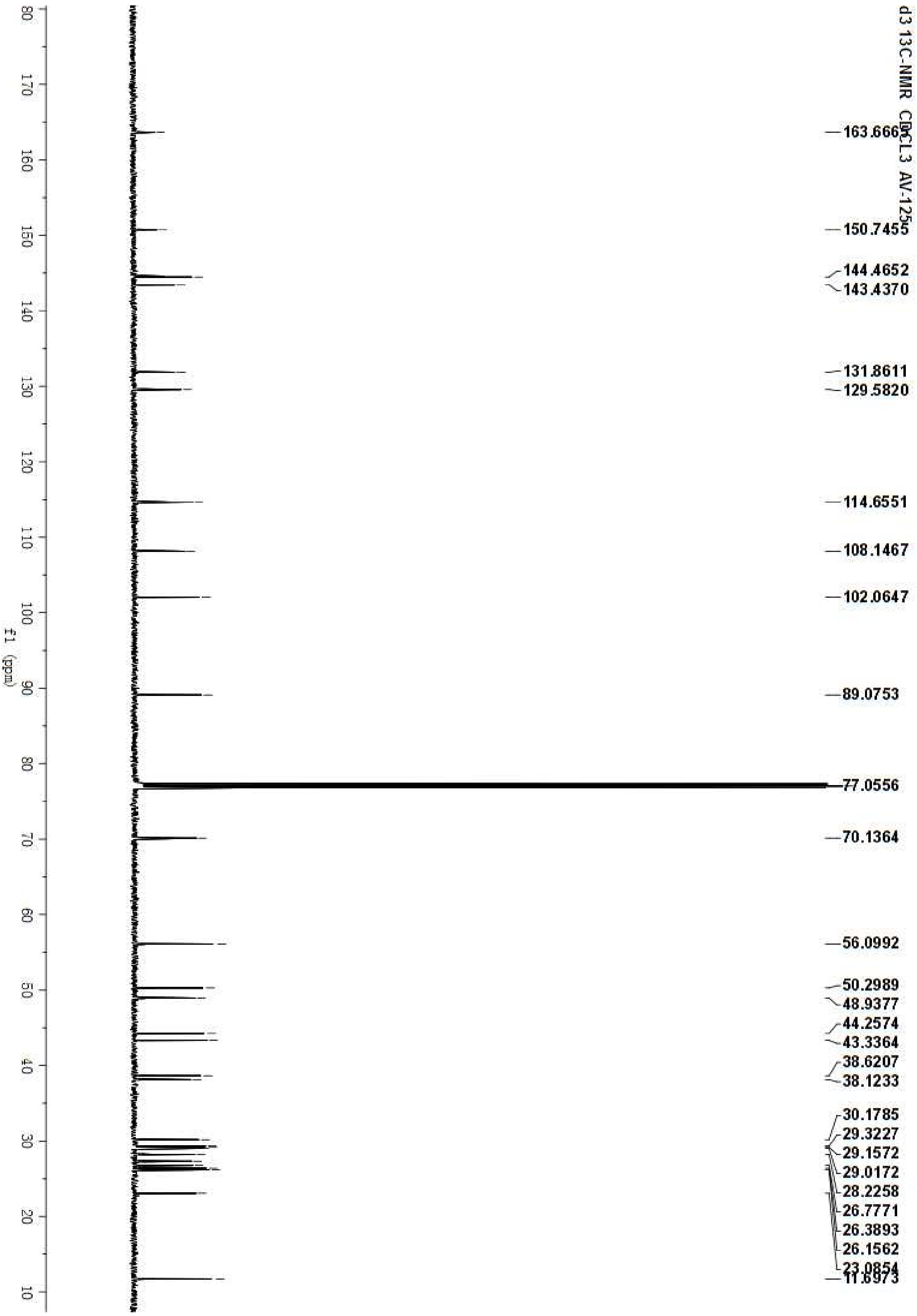

HRMS spectrum of **14a**

D:\data\.

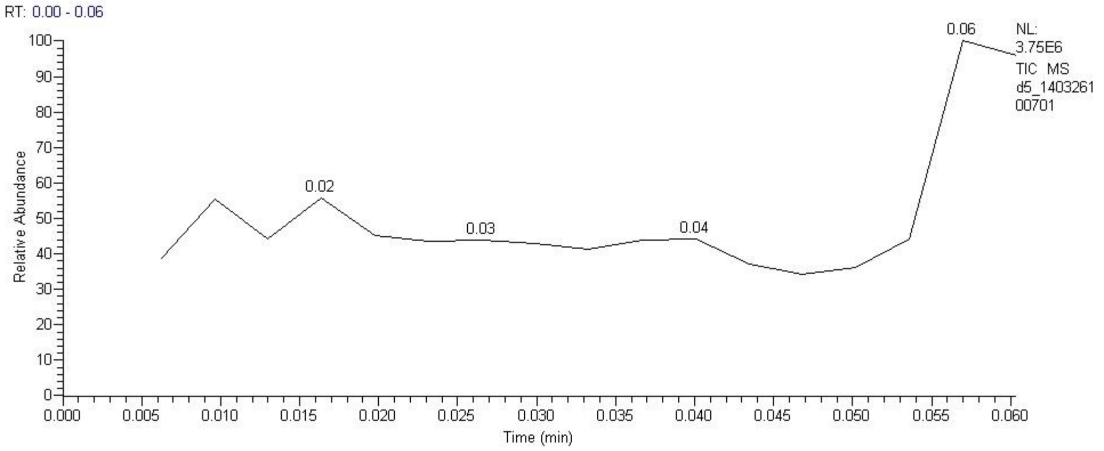

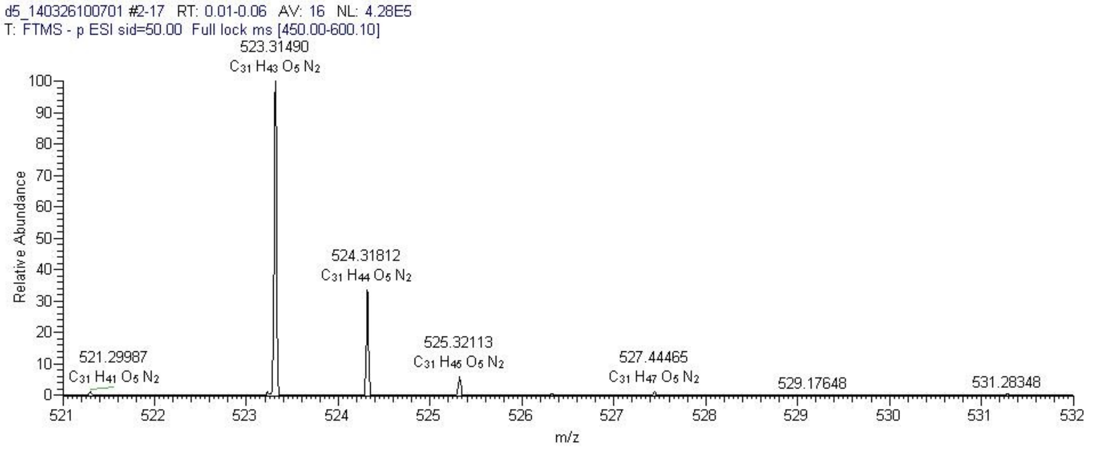

^{1}H NMR spectrum of **14b**

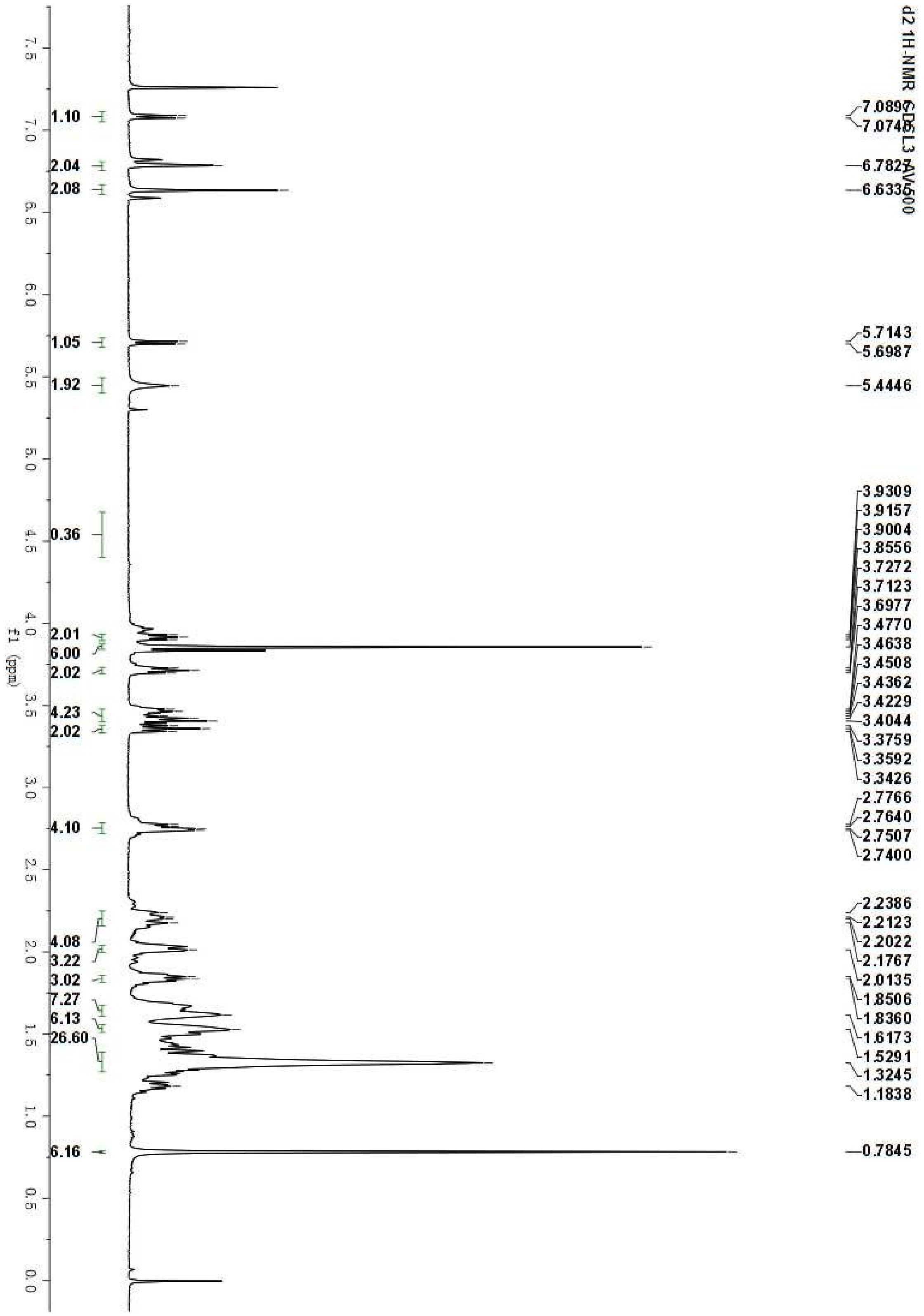

^{13}C NMR spectrum of **14b**

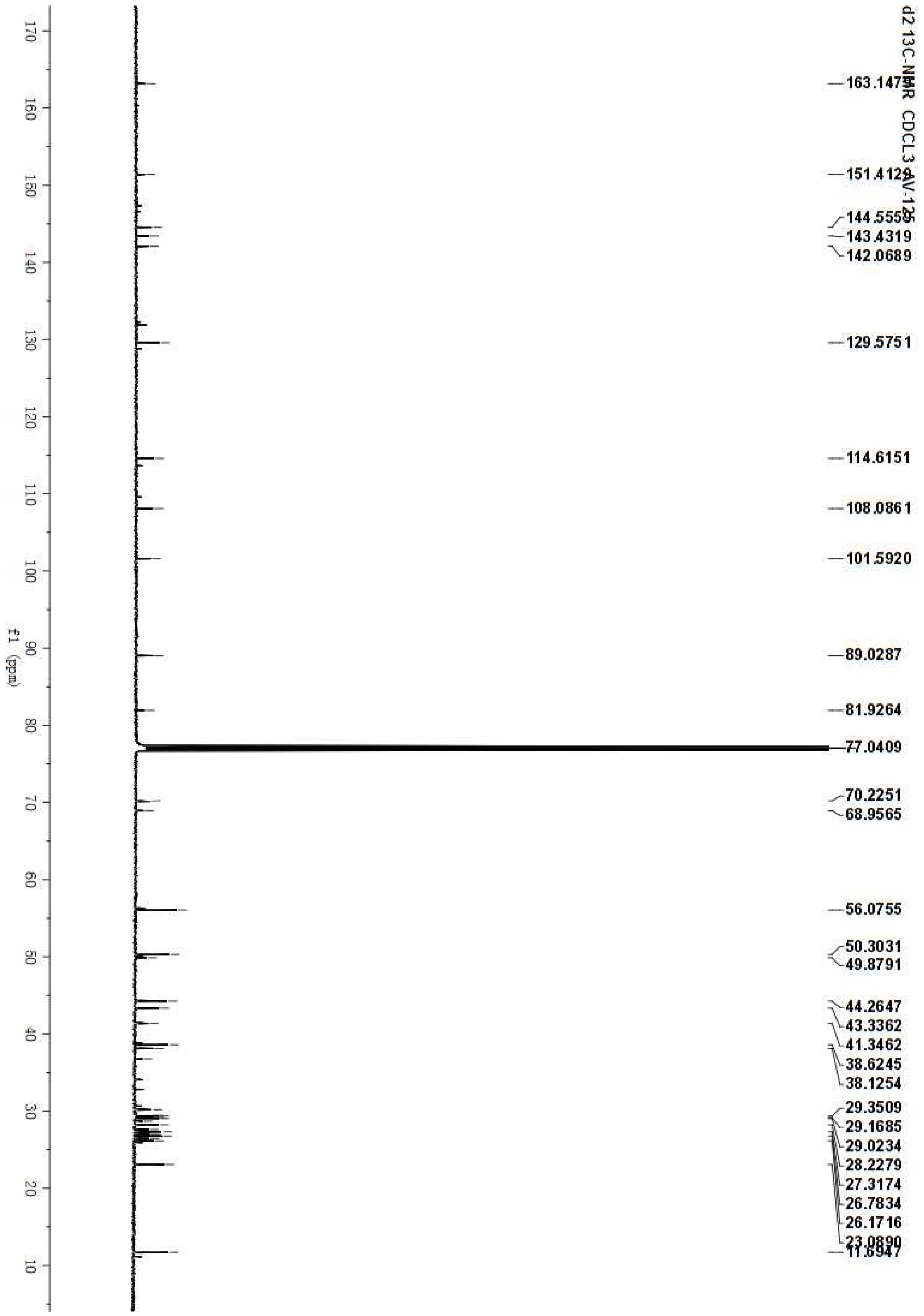

HRMS spectrum of **14b**

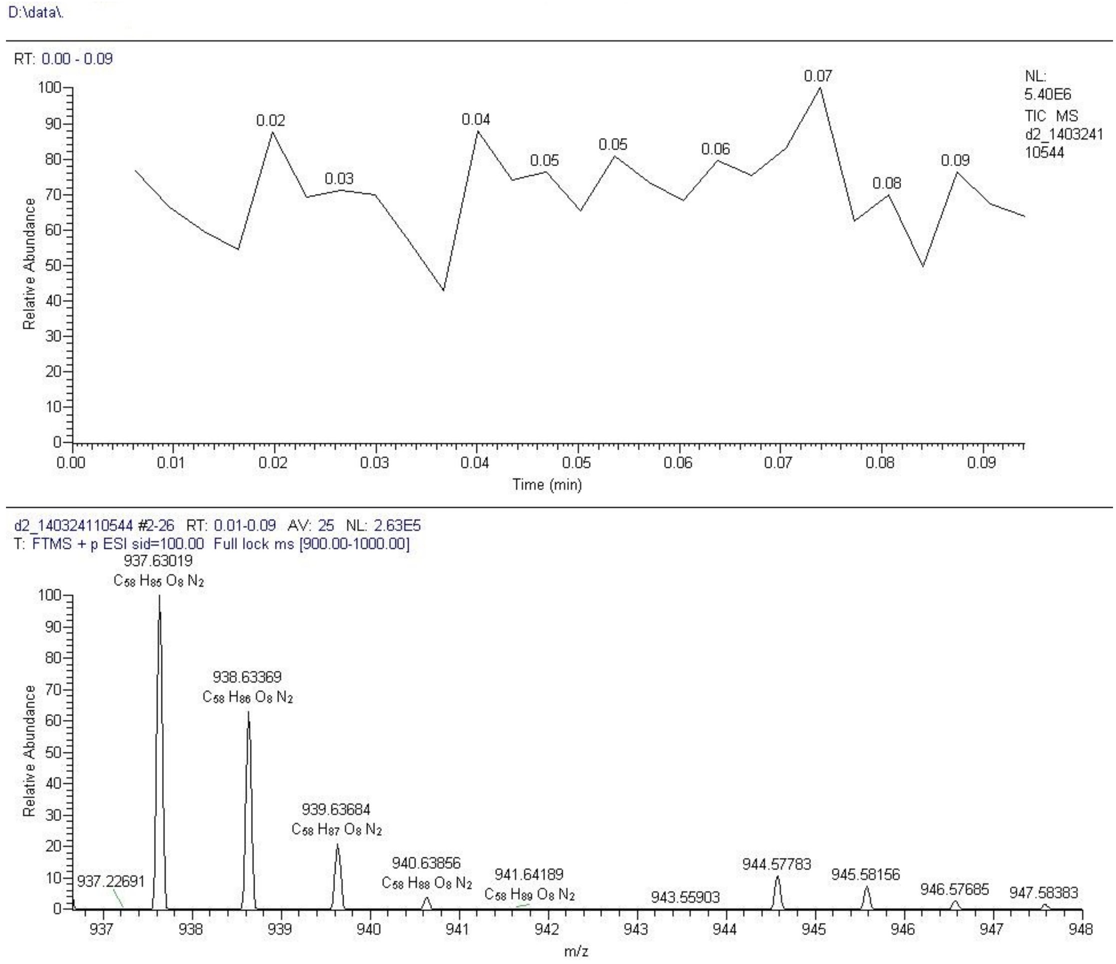

^{1}H NMR spectrum of **15a**

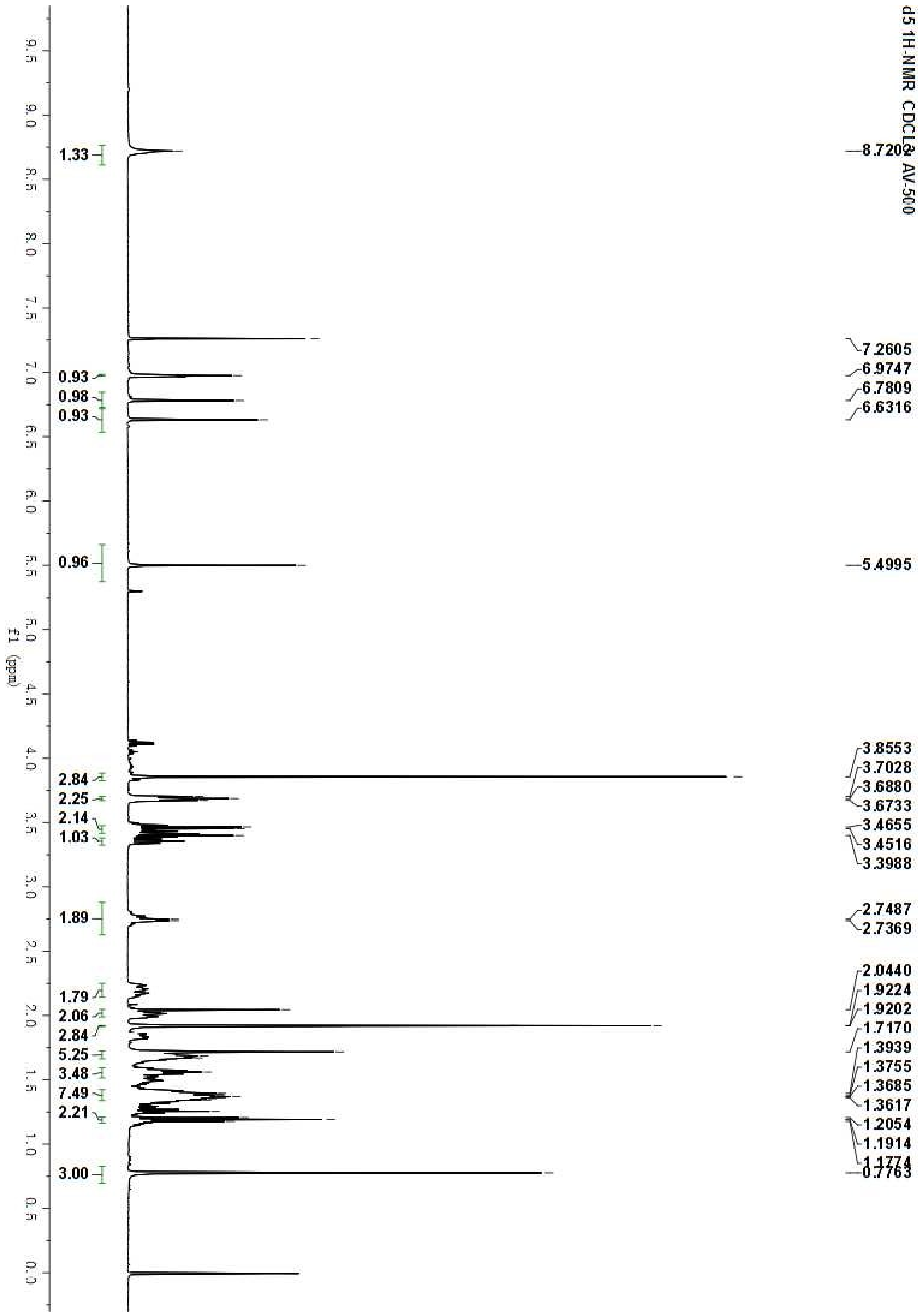

^{13}C NMR spectrum of **15a**

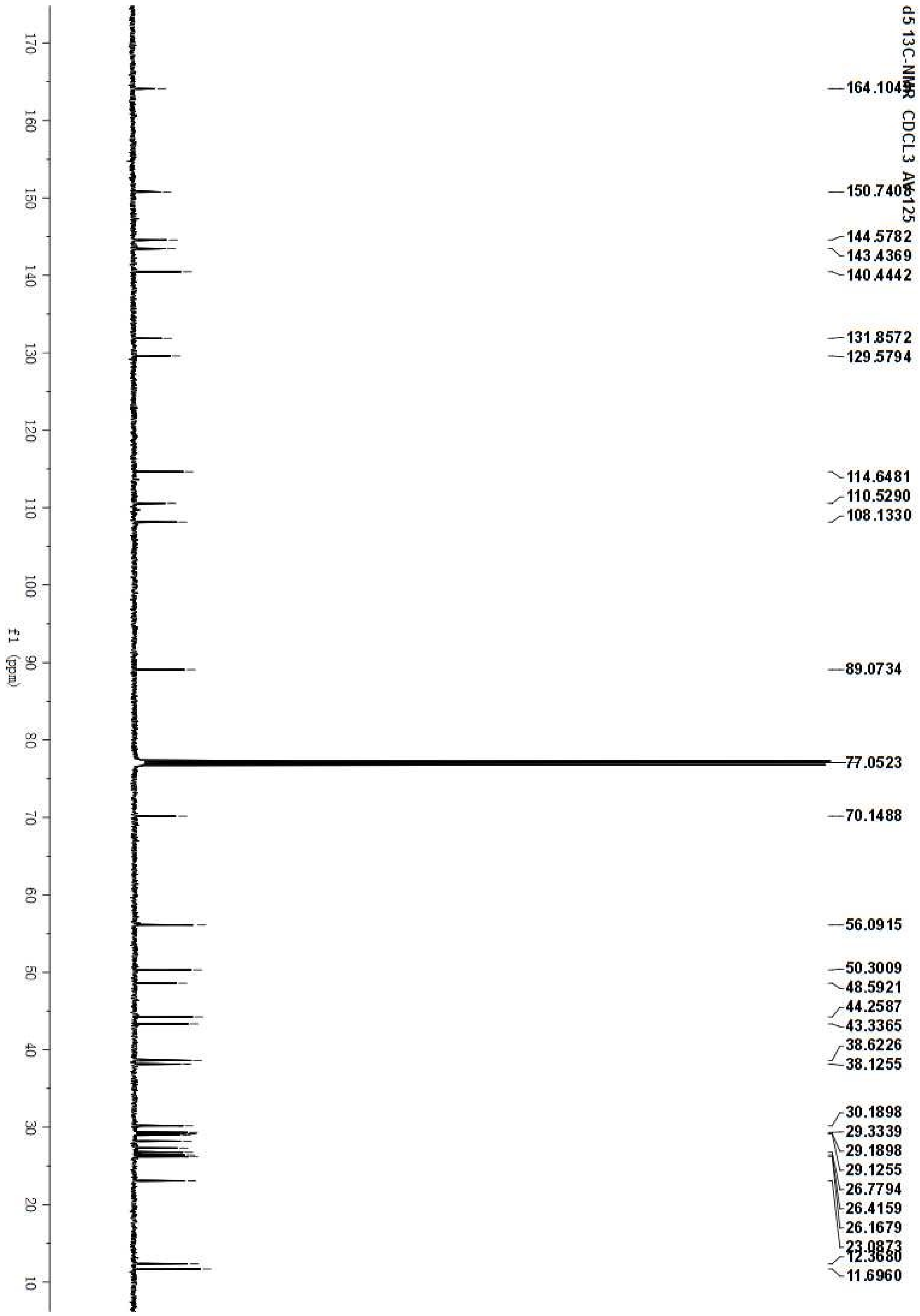

HRMS spectrum of **15a**

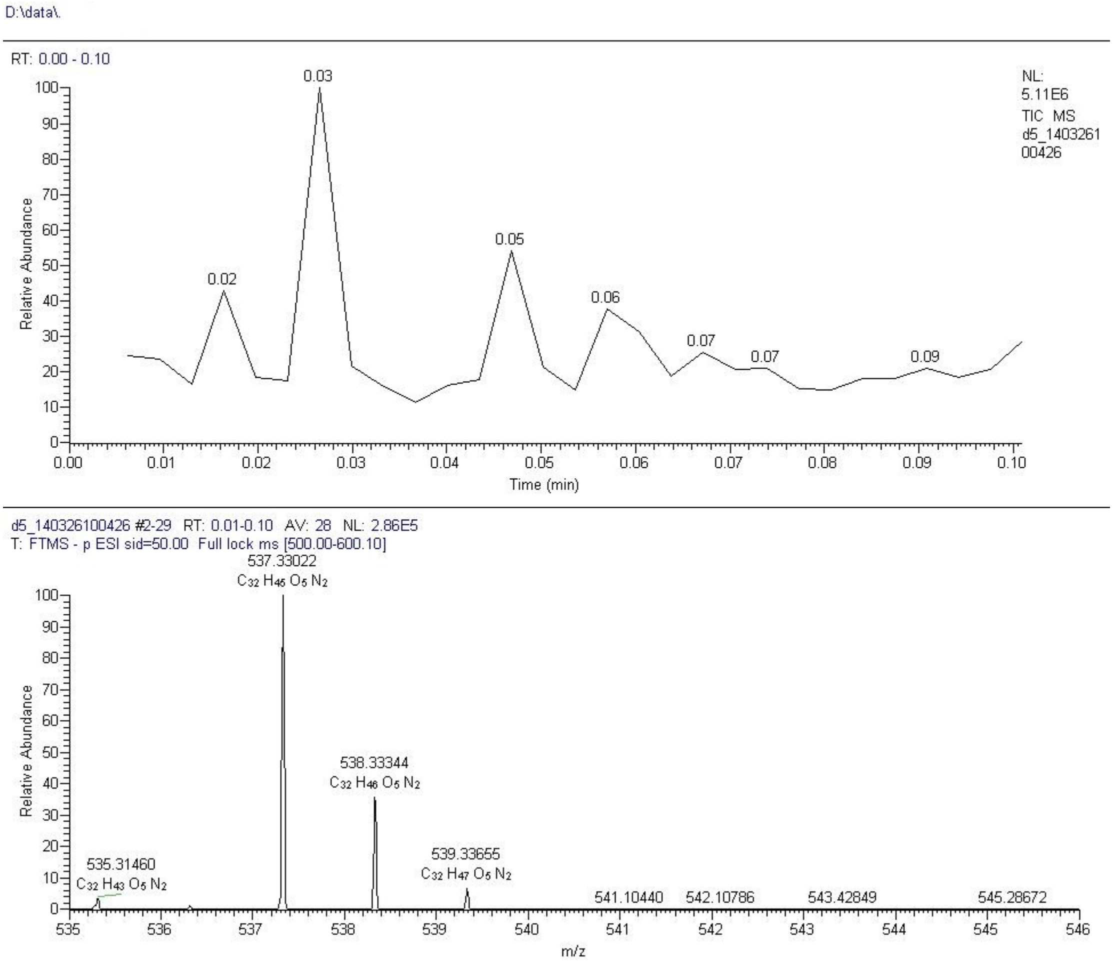

^{1}H NMR spectrum of **15b**

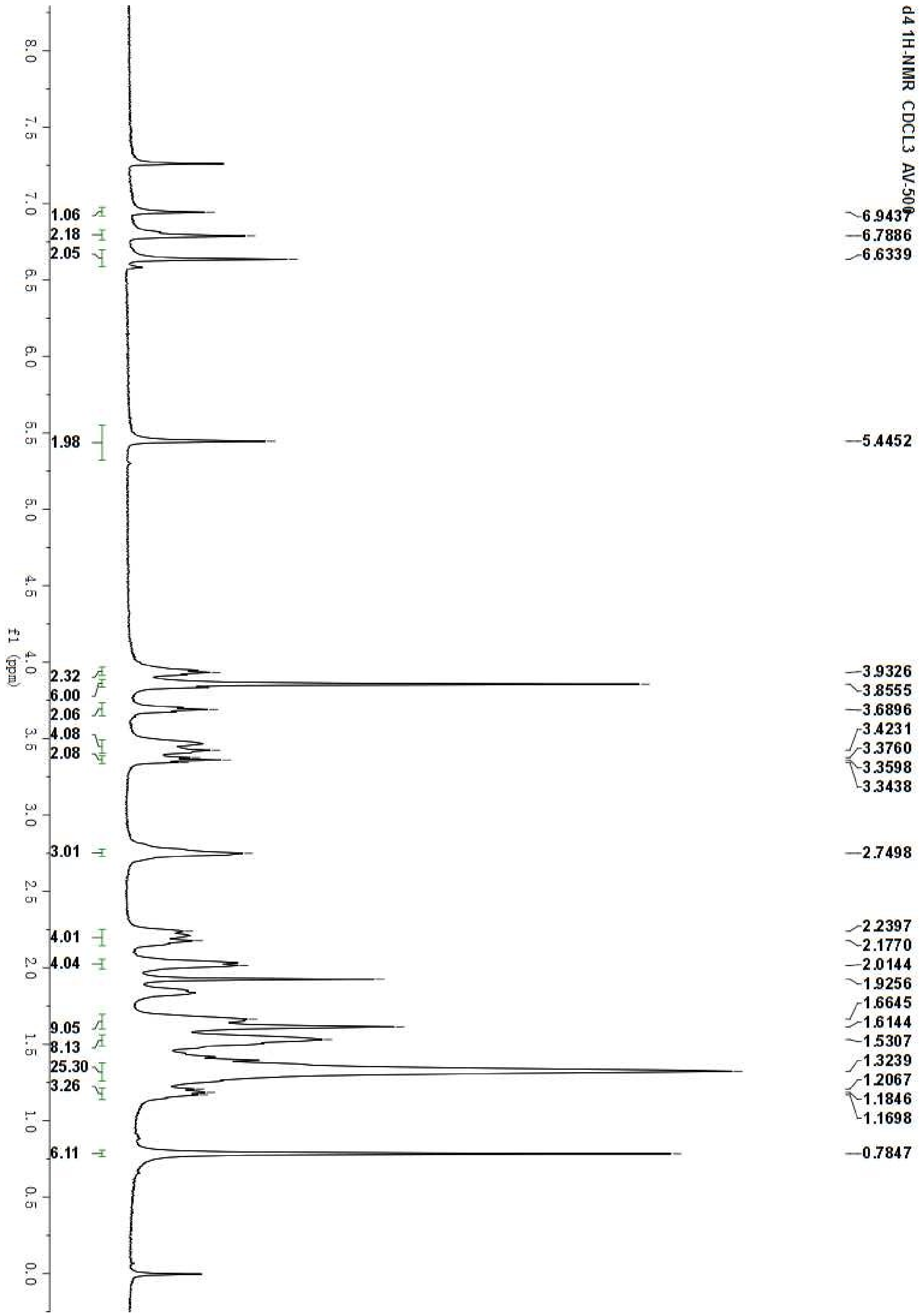

^{13}C NMR spectrum of **15b**

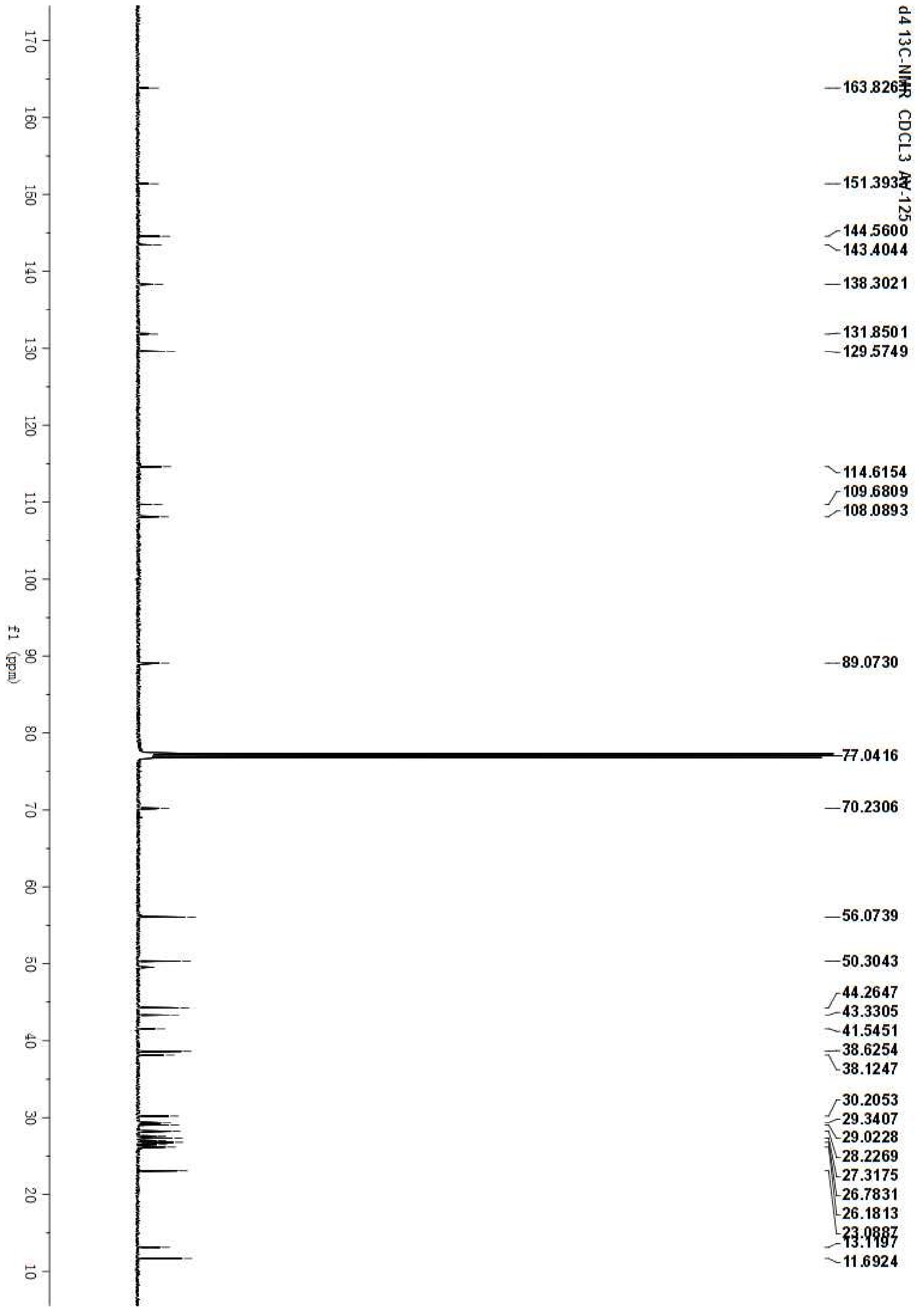

HRMS spectrum of **15b**

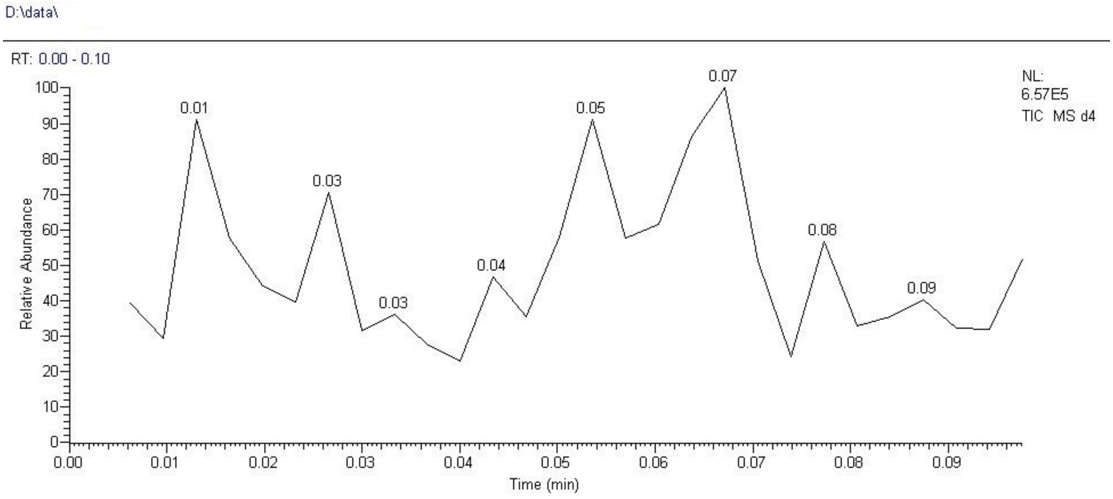

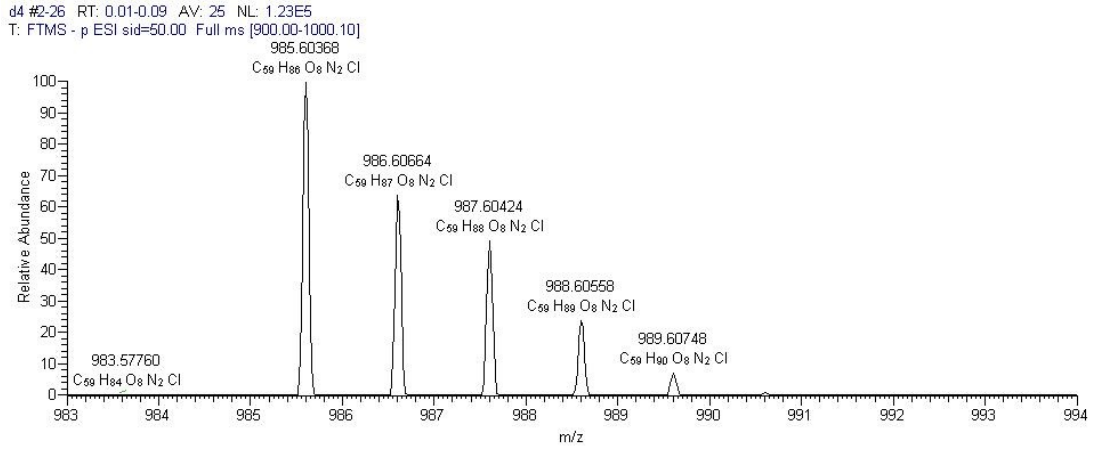

^{1}H NMR spectrum of **16**

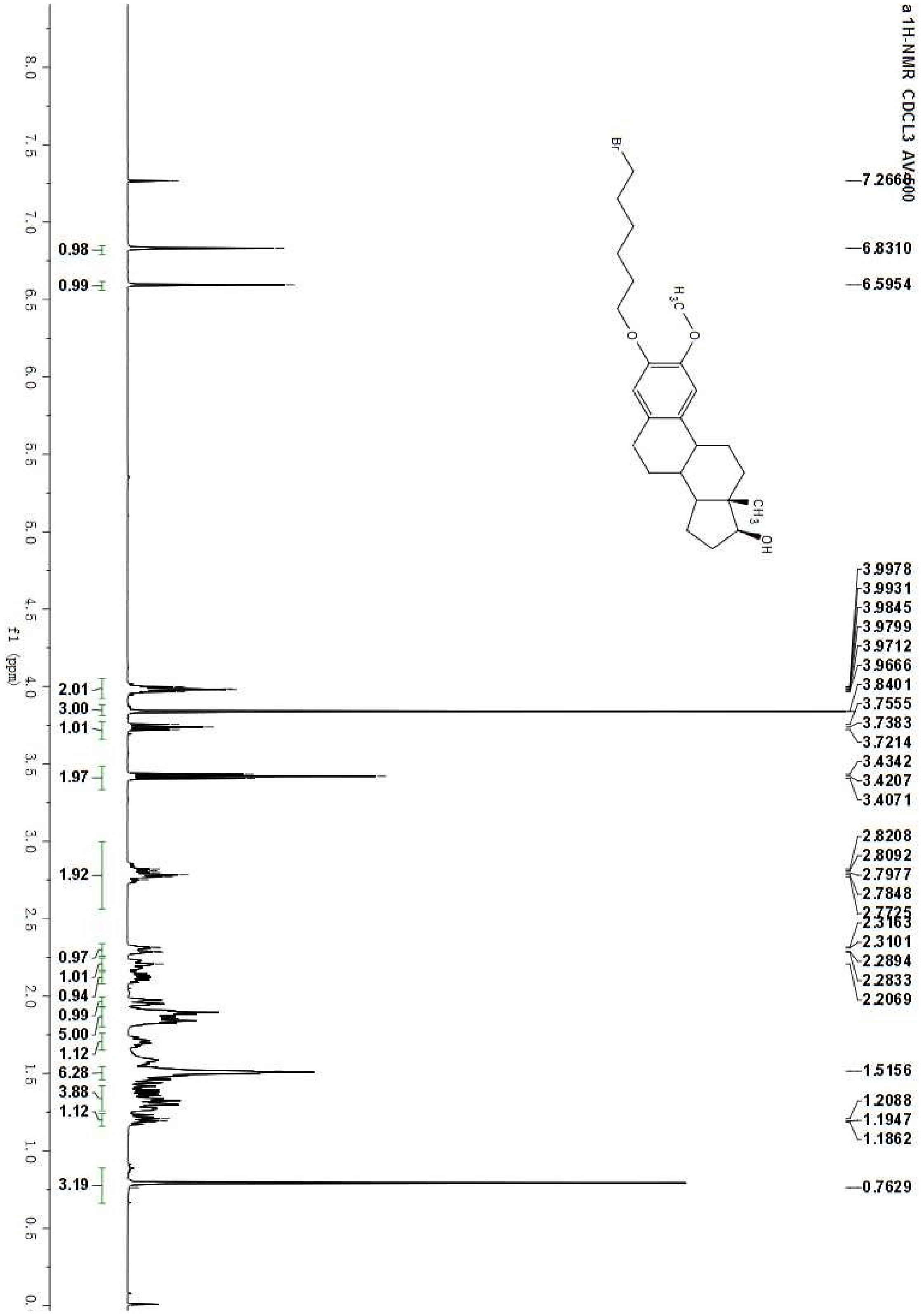

HRMS spectrum of **16**

**Sample Name**		**Position**	P2-A1	**Instrument Name**	Instrument 1	**User Name**	
**Inj Vol**	0.1	**InjPosition**		**SampleType**	Sample	**IRM Calibration Status**	Success
**Data Filename**	a-p.d	**ACQ Method**		**Comment**		**Acquired Time**	

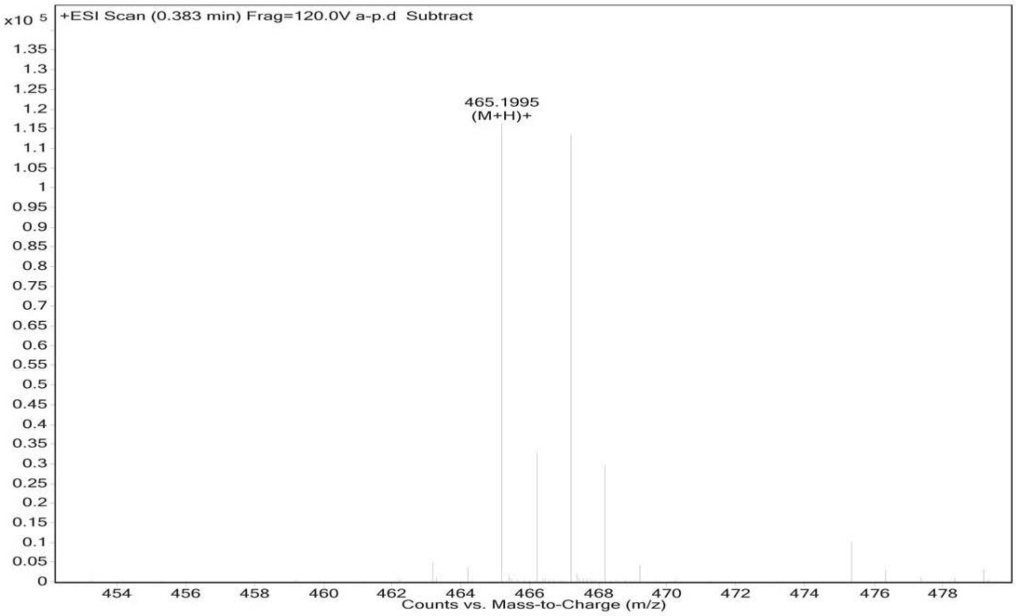

^{1}H NMR spectrum of **17**

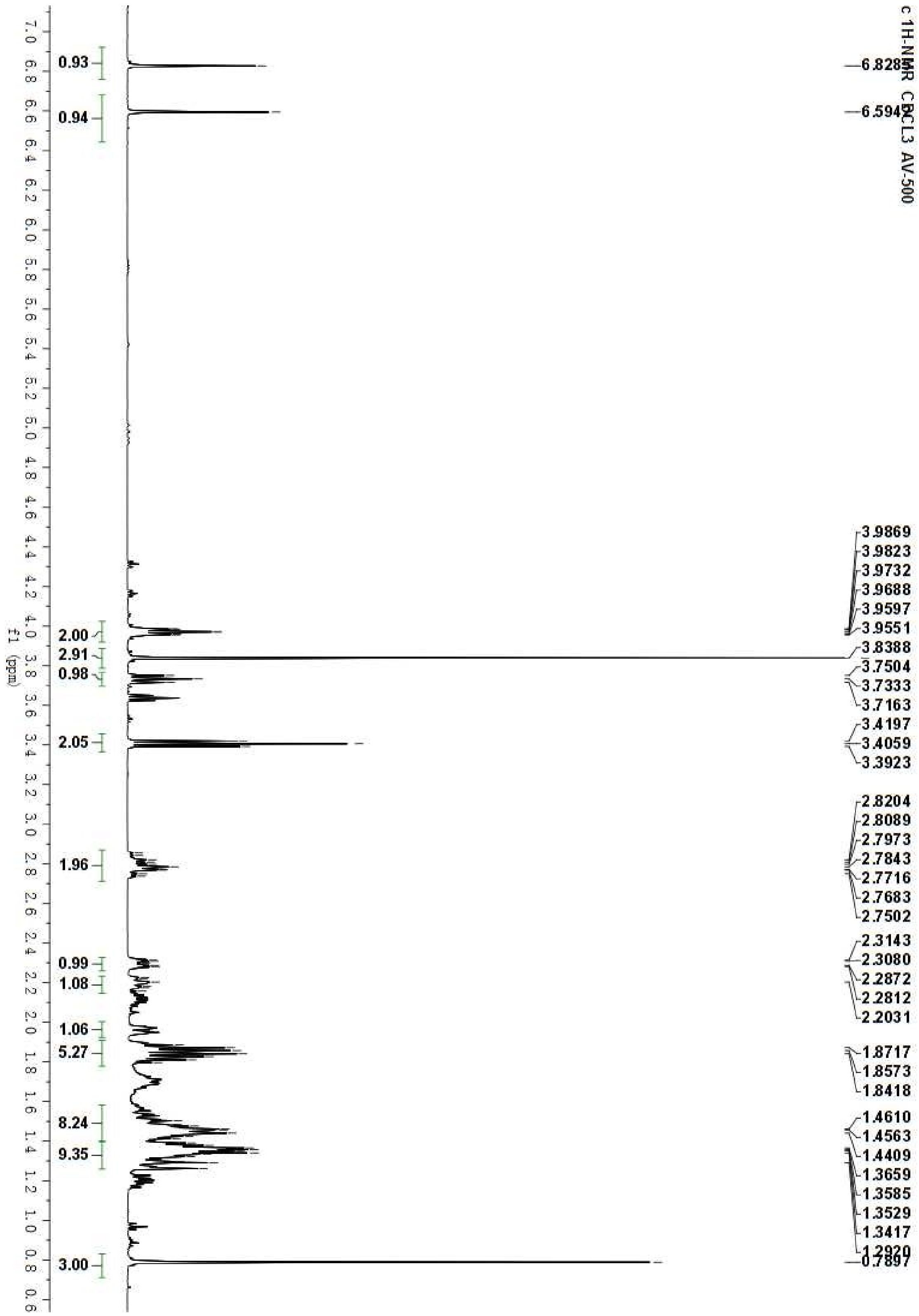

HRMS spectrum of **17**

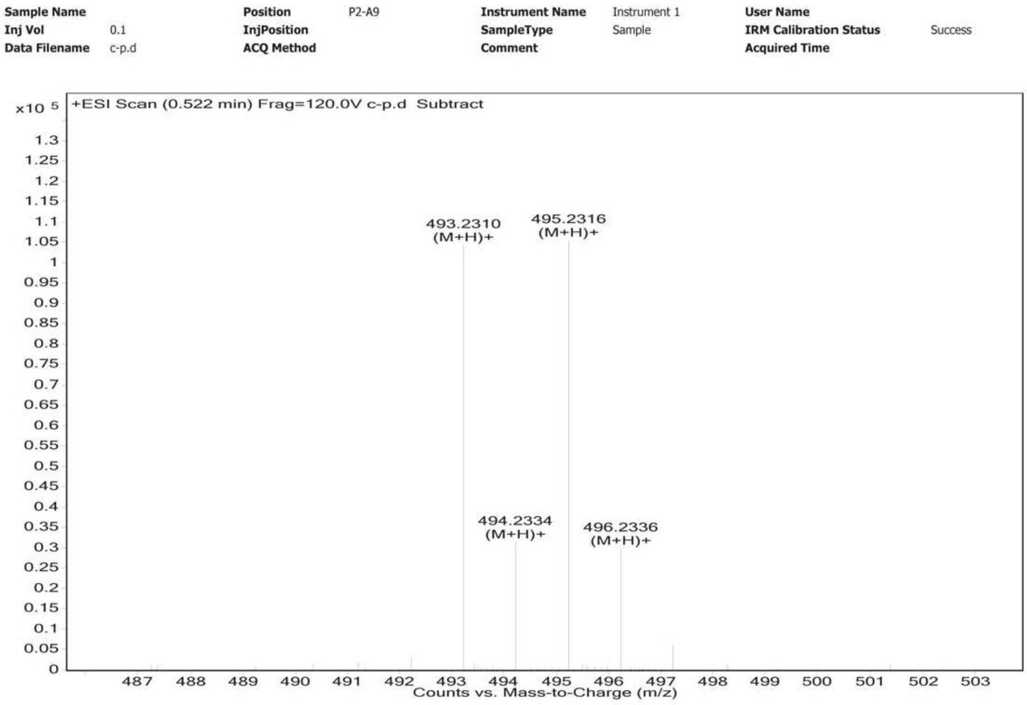

^{1}H NMR spectrum of **18**

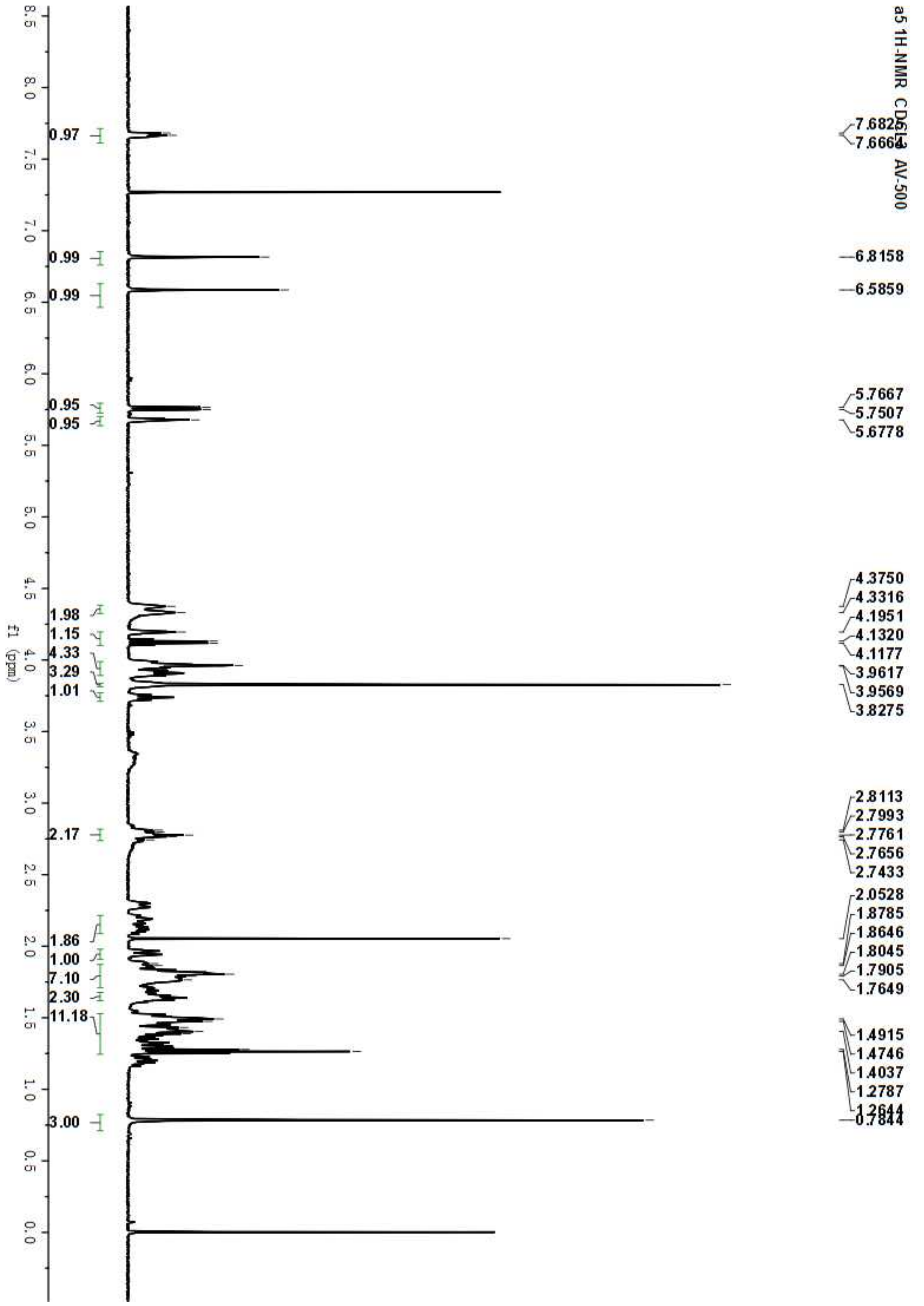

^{13}C NMR spectrum of **18**

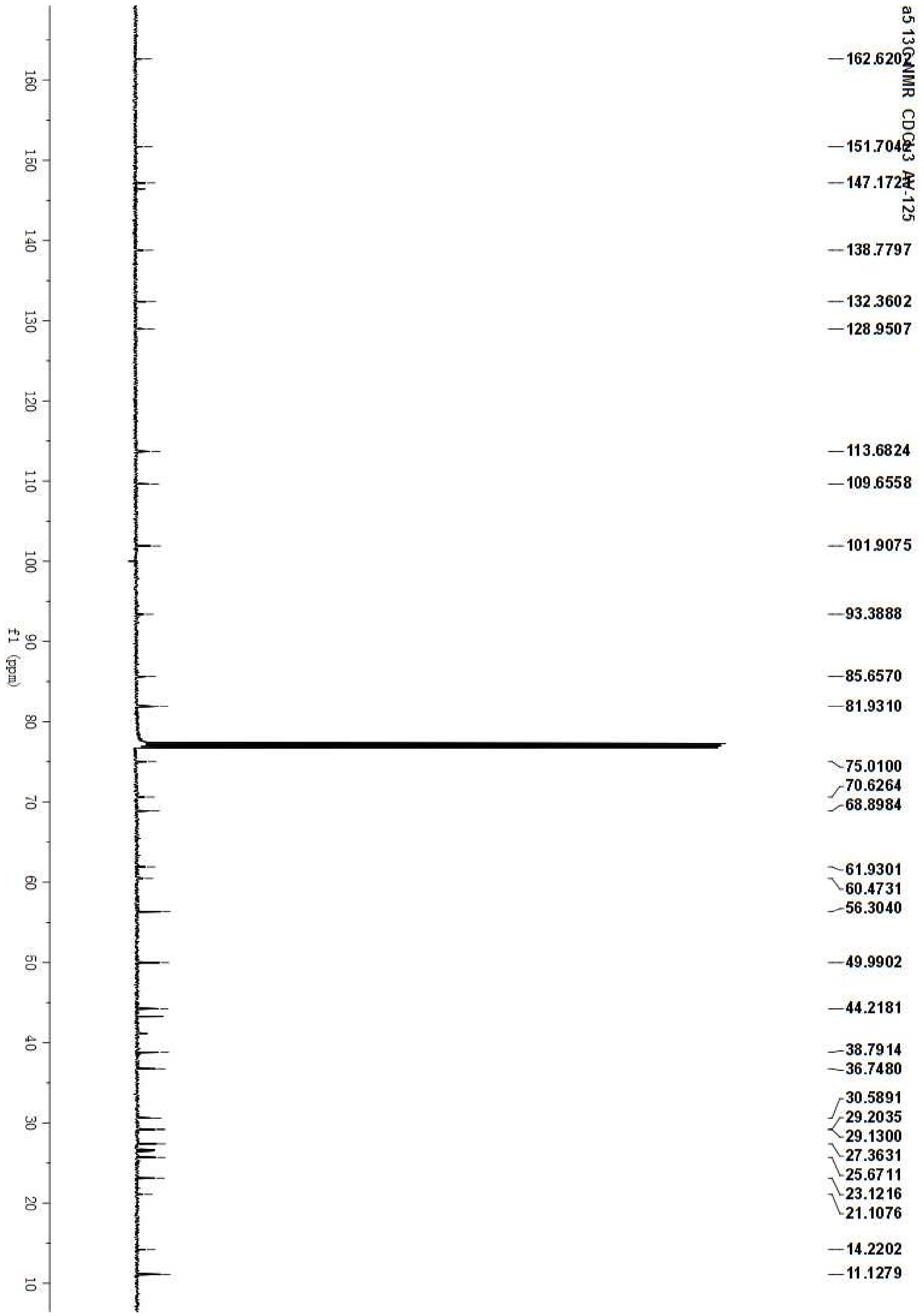

HRMS spectrum of **18**

Sample Name		Position	P2-A5	Instrument Name	Instrument 1	User Name	
Inj Vol	0.1	InjPosition		SampleType	Sample	IRM Calibration Status	Success
Data Filename	a5-p.d	ACQ Method		Comment		Acquired Time	

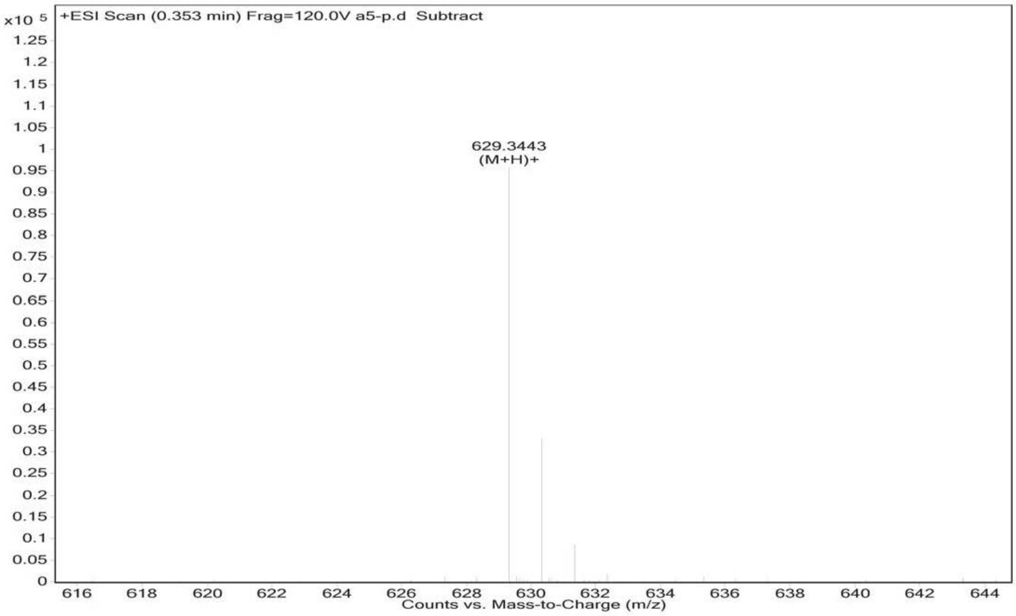

^{1}H NMR spectrum of **19**

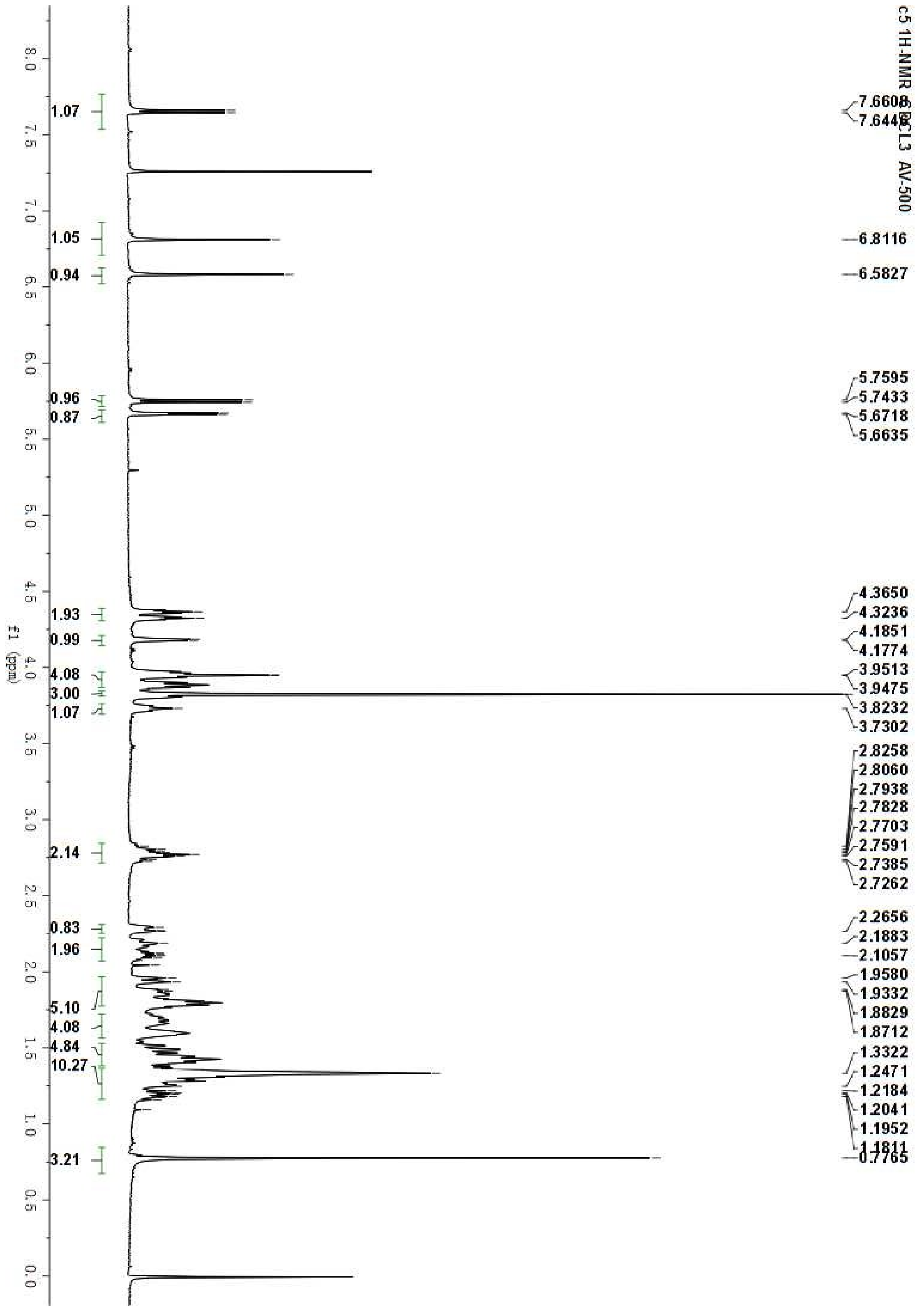

^{13}C NMR spectrum of **19**

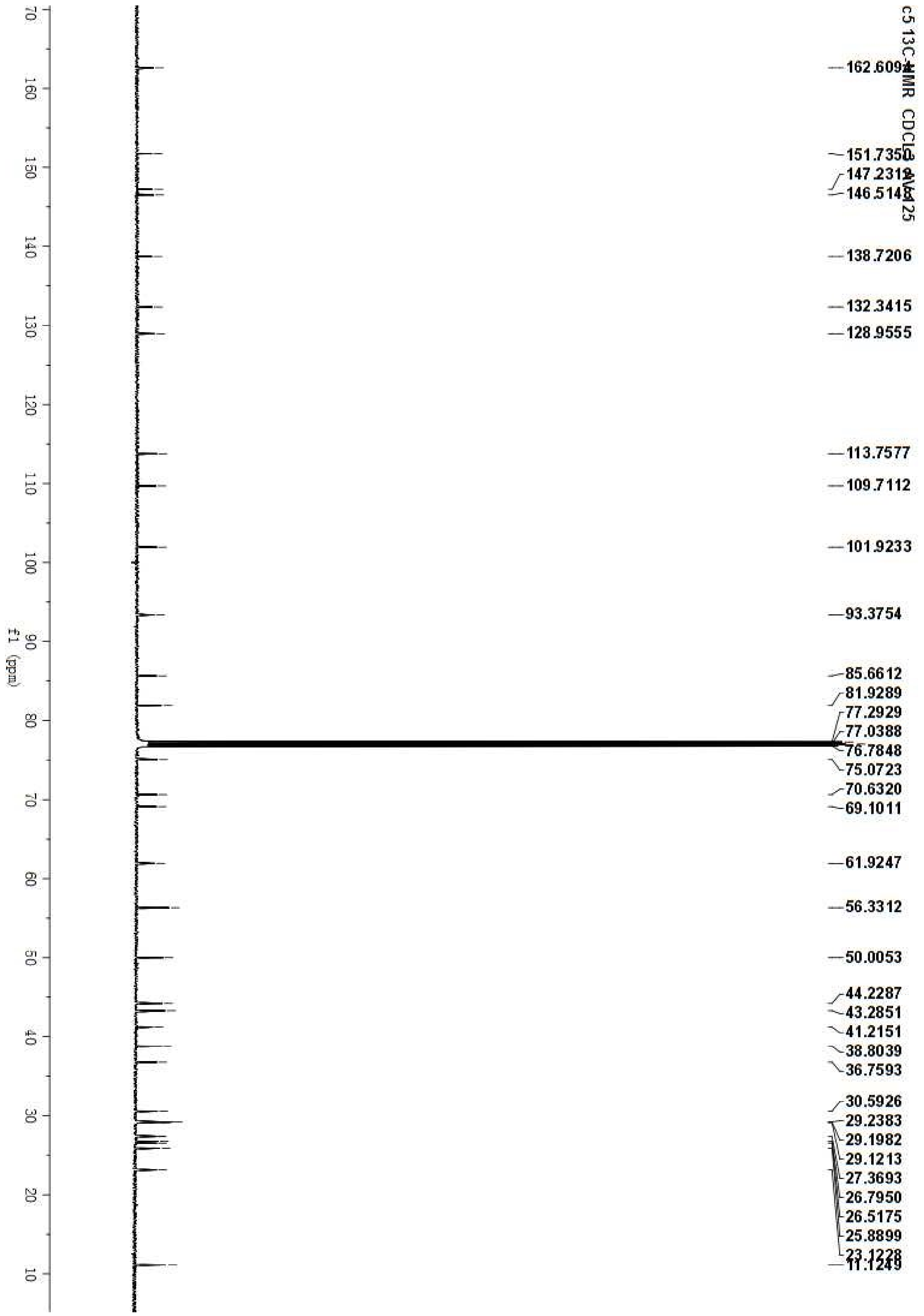

HRMS spectrum of **19**

Sample Name		Position	P2-B5	Instrument Name	Instrument 1	User Name	
Inj Vol	0.1	InjPosition		SampleType	Sample	IRM Calibration Status	Success
Data Filename	c5-p.d	ACQ Method		Comment		Acquired Time	

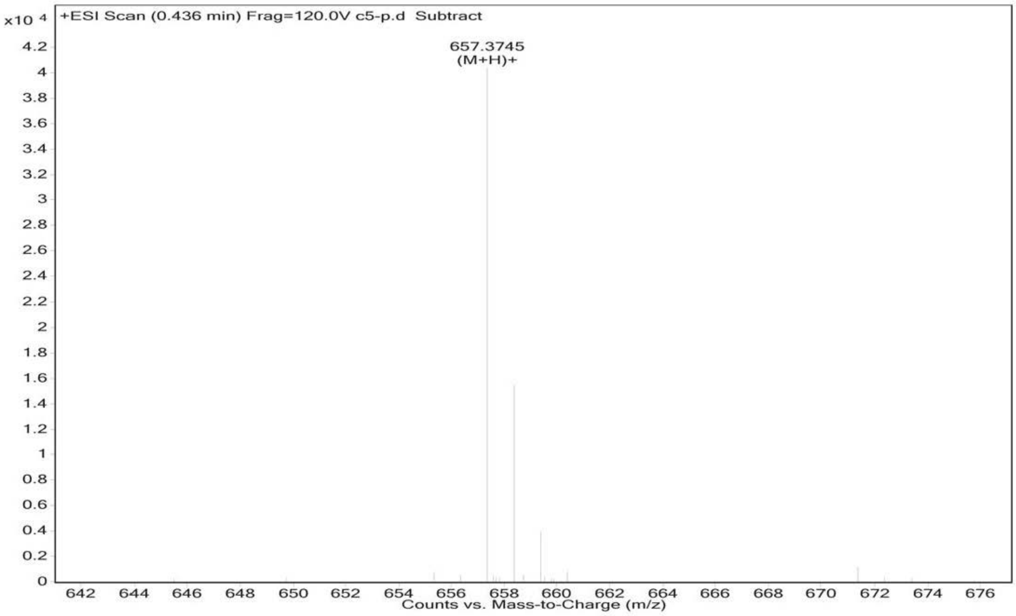

^{1}H NMR spectrum of **20a**

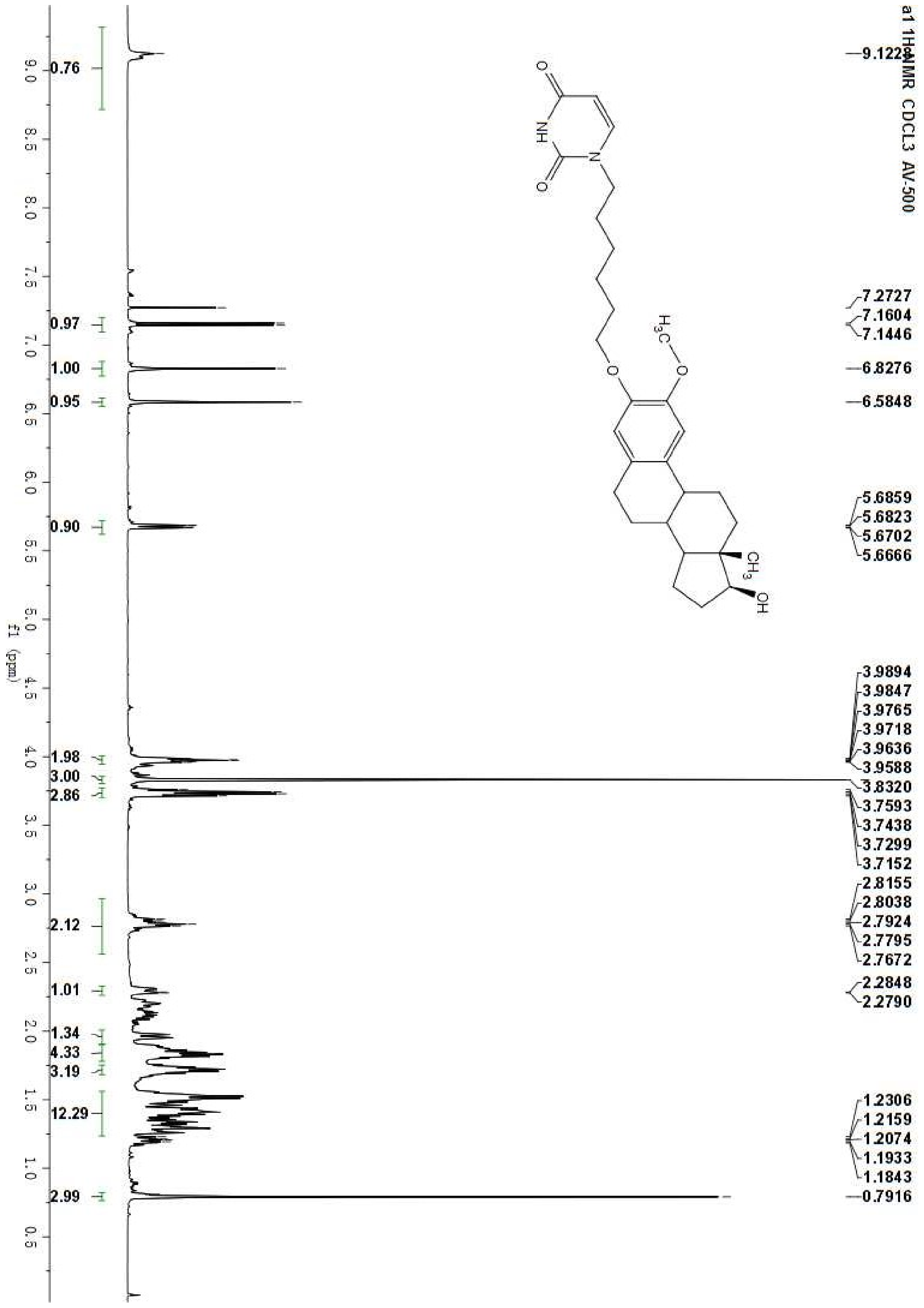

^{13}C NMR spectrum of **20a**

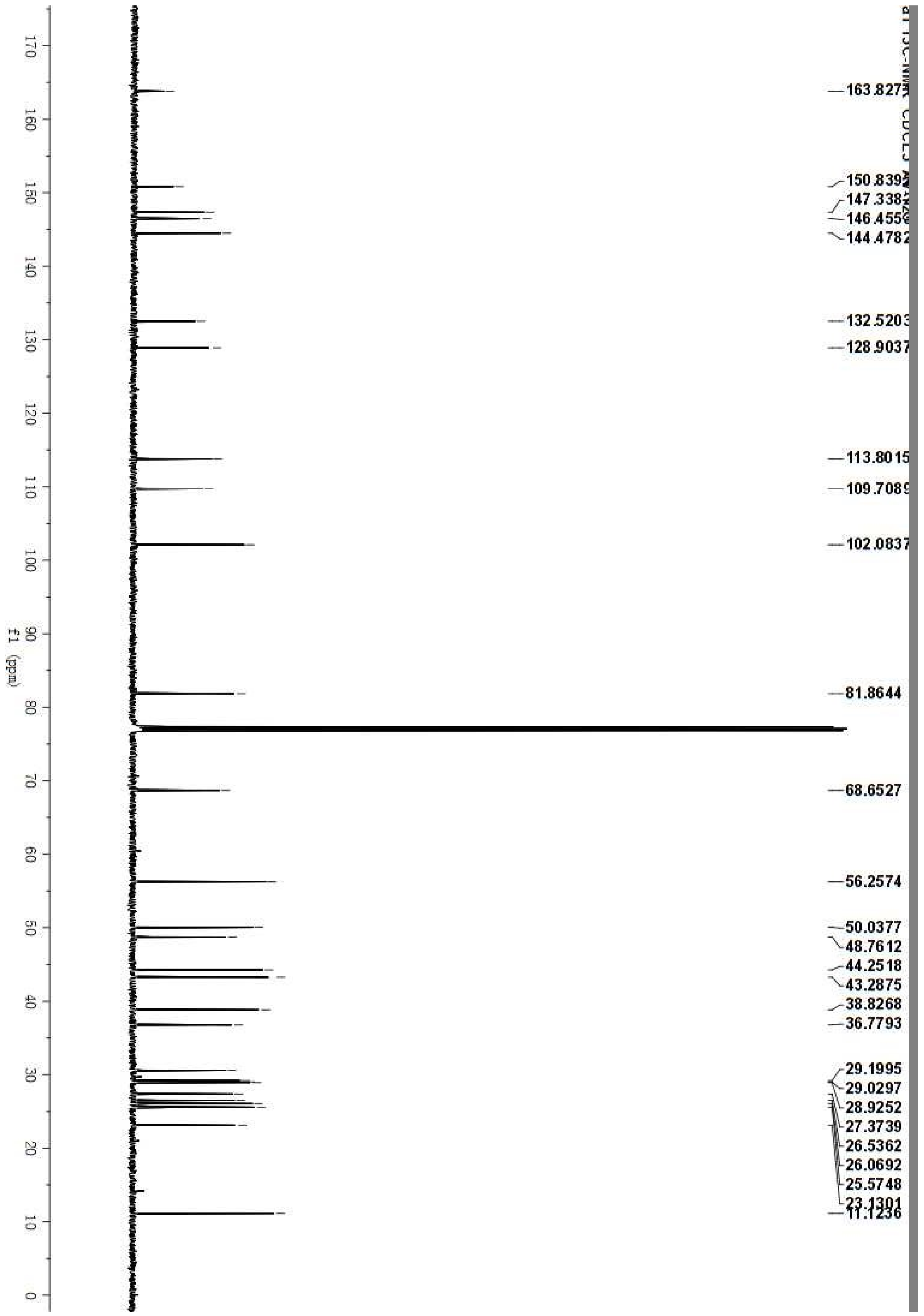

HRMS spectrum of **20a**

**Sample Name**		**Position**	P2-A2	**Instrument Name**	Instrument 1	**User Name**	
**Inj Vol**	0.1	**InjPosition**		**SampleType**	Sample	**IRM Calibration Status**	Success
**Data Filename**	a1-p.d	**ACQ Method**		**Comment**		**Acquired Time**	

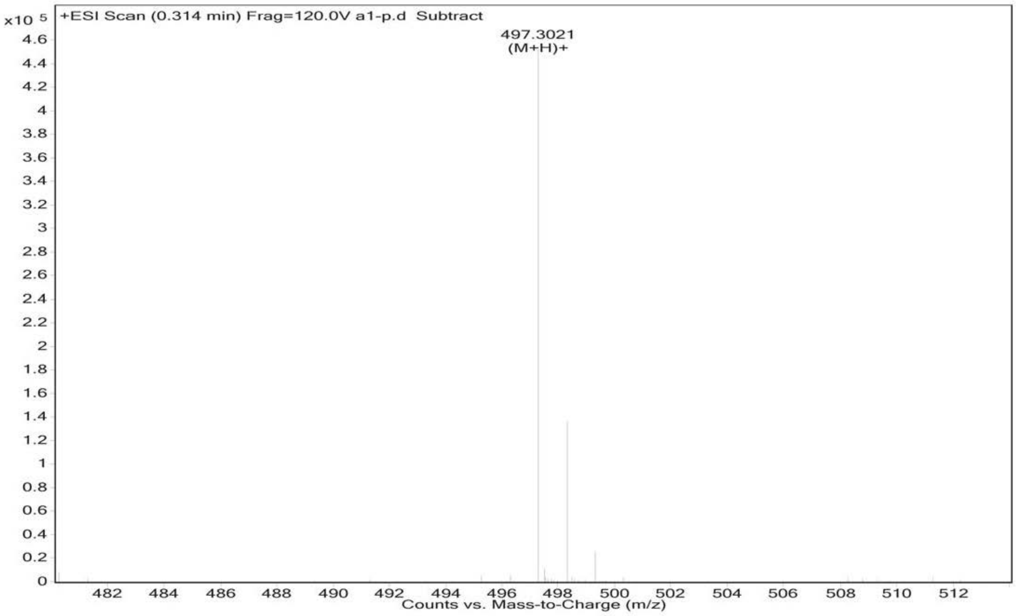

^{1}H NMR spectrum of **20b**

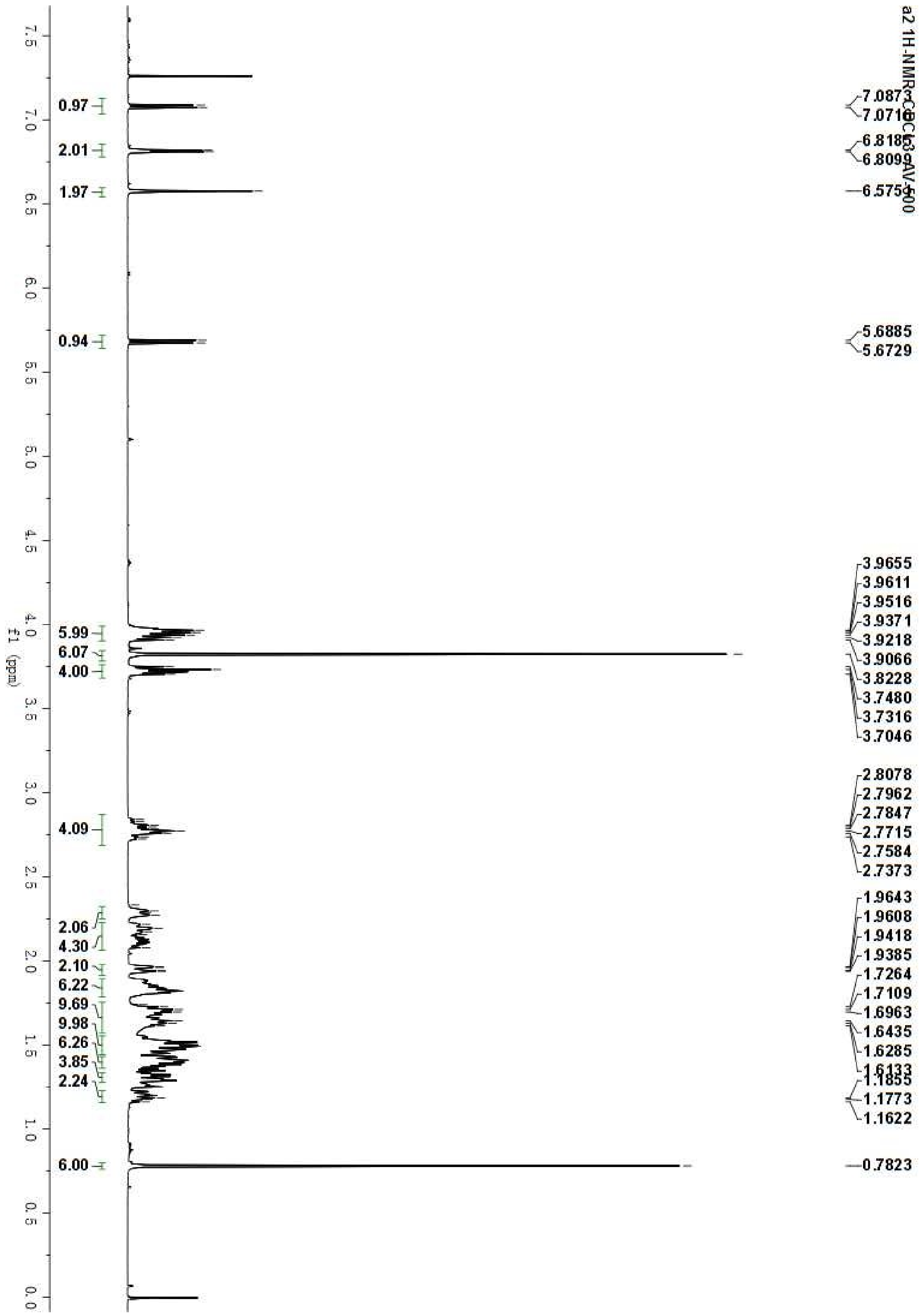

^{13}C NMR spectrum of **20b**

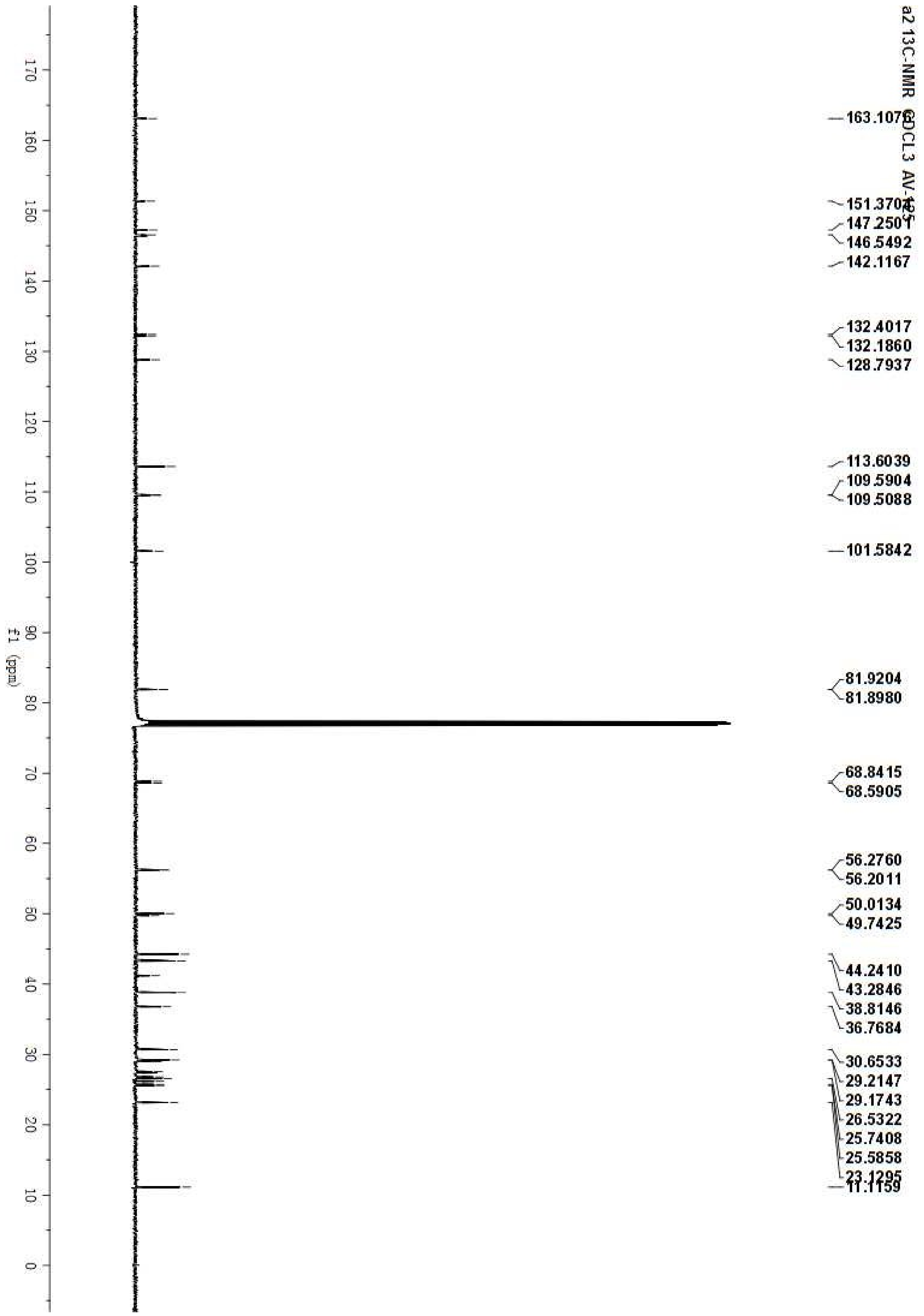

HRMS spectrum of **20b**

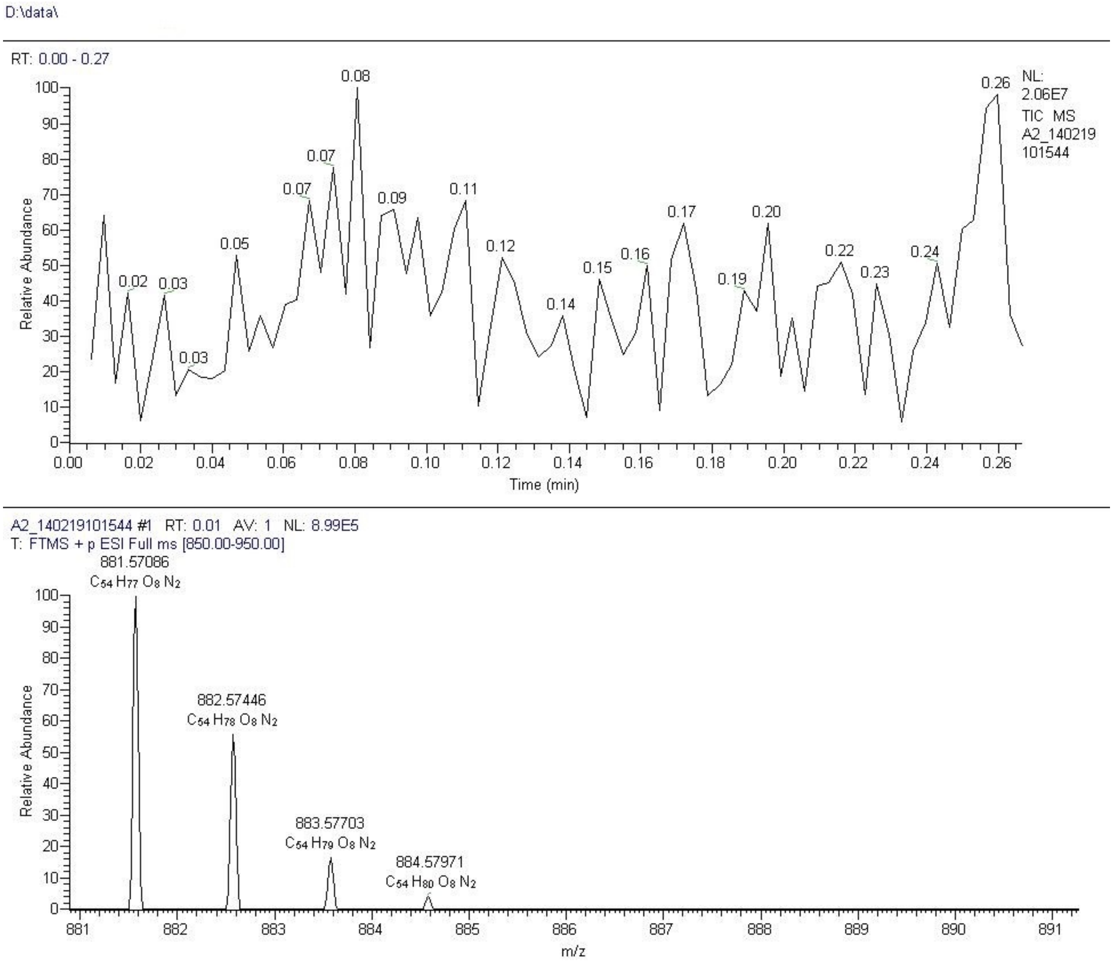

^{1}H NMR spectrum of **21a**

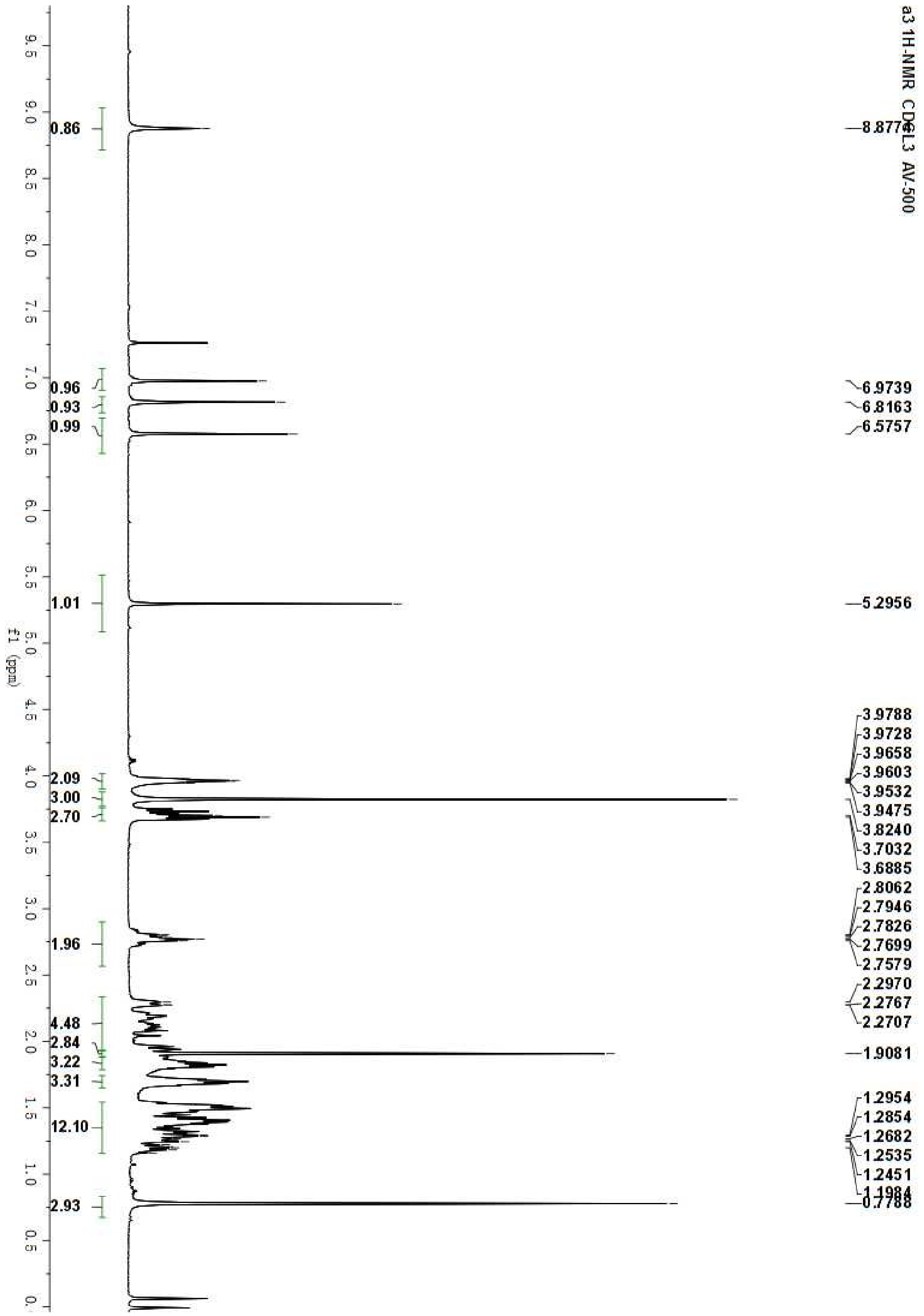

^{13}C NMR spectrum of **21a**

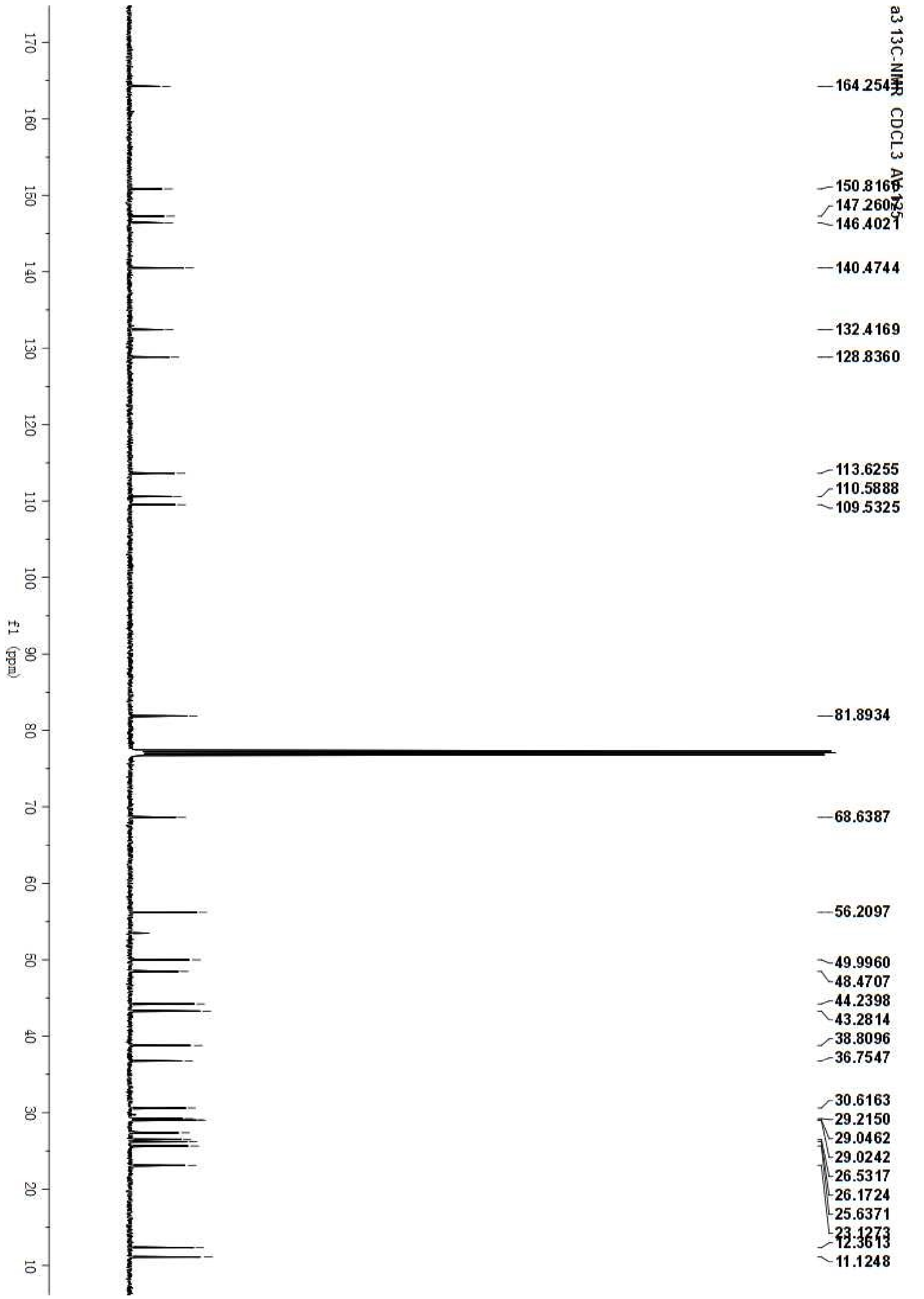

HRMS spectrum of **21a**

**Sample Name**		**Position**	P2-A3	**Instrument Name**	Instrument 1	**User Name**	
**Inj Vol**	0.1	**InjPosition**		**SampleType**	Sample	**IRM Calibration Status**	Success
**Data Filename**	a3-p.d	**ACQ Method**		**Comment**		**Acquired Time**	

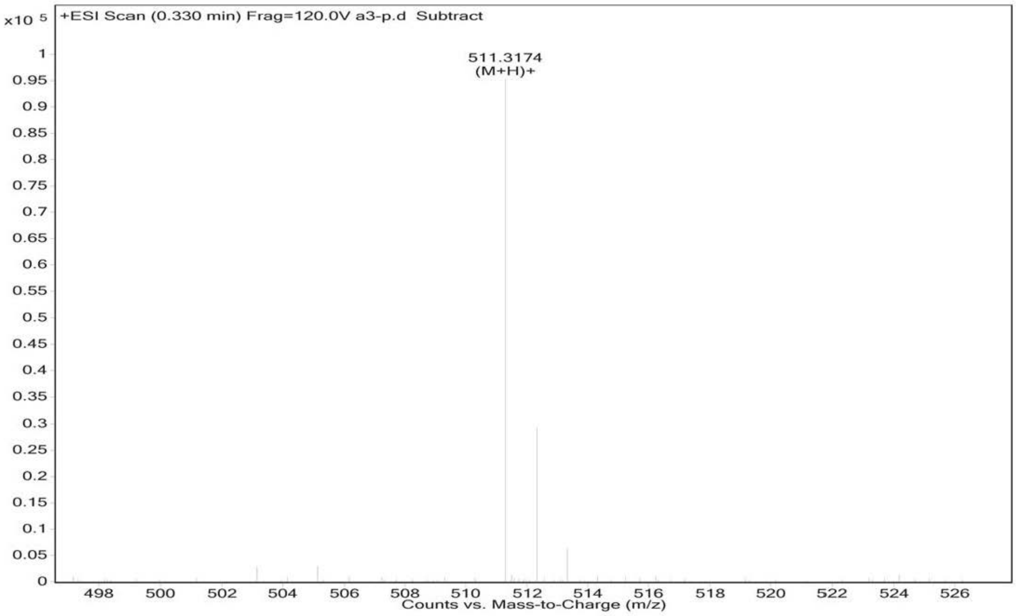

^{1}H NMR spectrum of **21b**

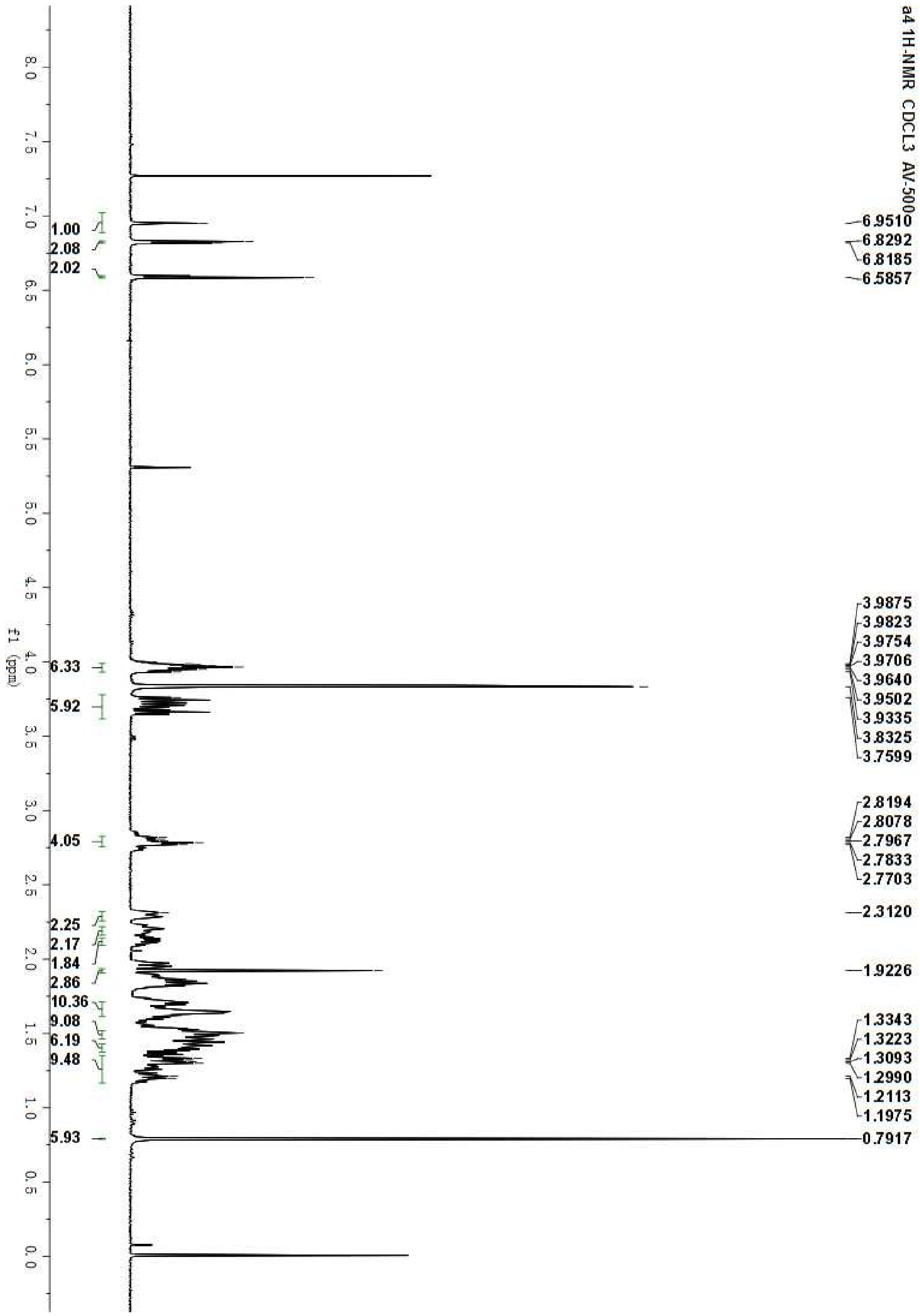

^{13}C NMR spectrum of **21b**

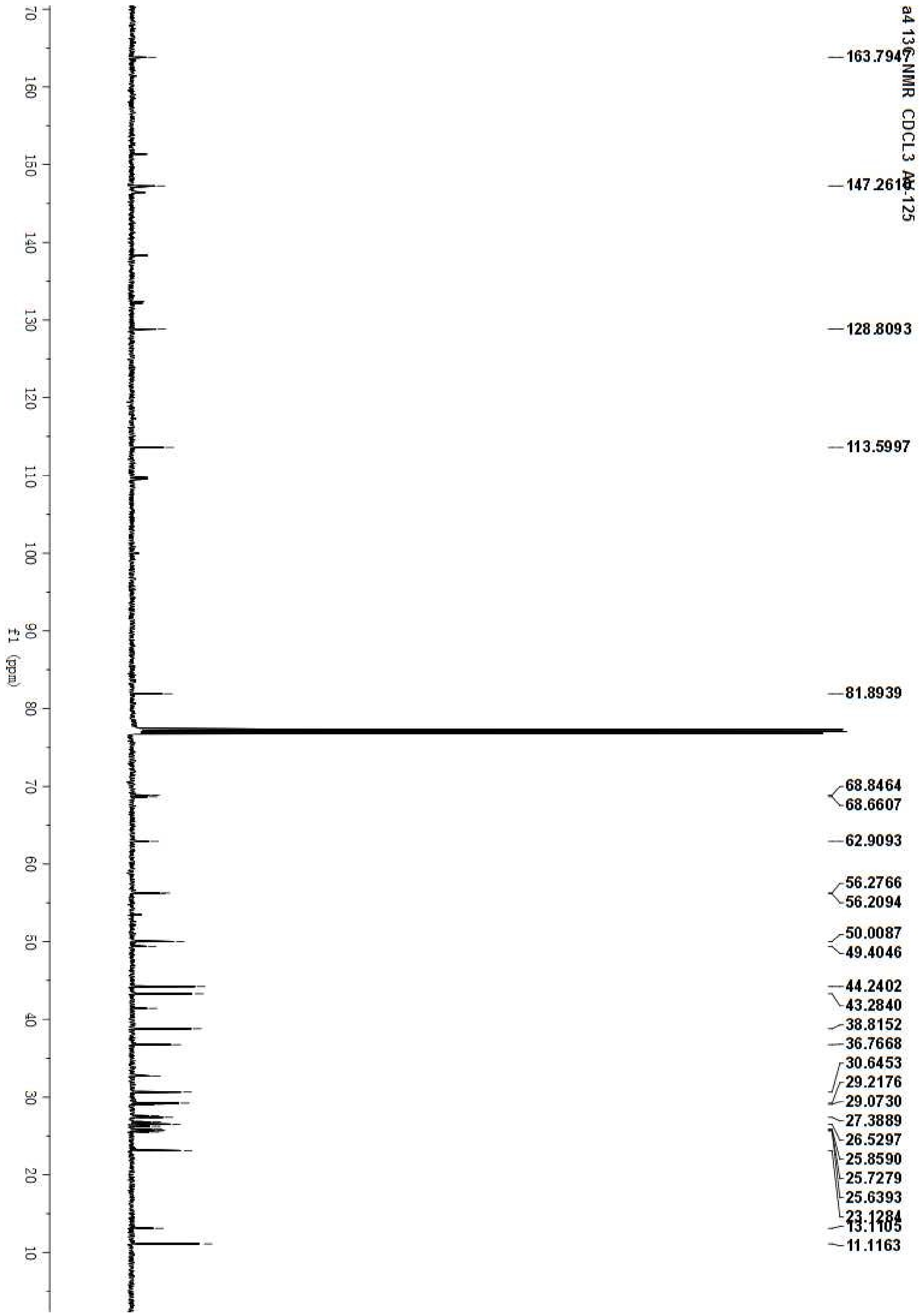

HRMS spectrum of **21b**

**Sample Name**		**Position**	P2-A4	**Instrument Name**	Instrument 1	**User Name**	
**Inj Vol**	0.1	**InjPosition**		**SampleType**	Sample	**IRM Calibration Status**	Success
**Data Filename**	a4-p.d	**ACQ Method**		**Comment**		**Acquired Time**	

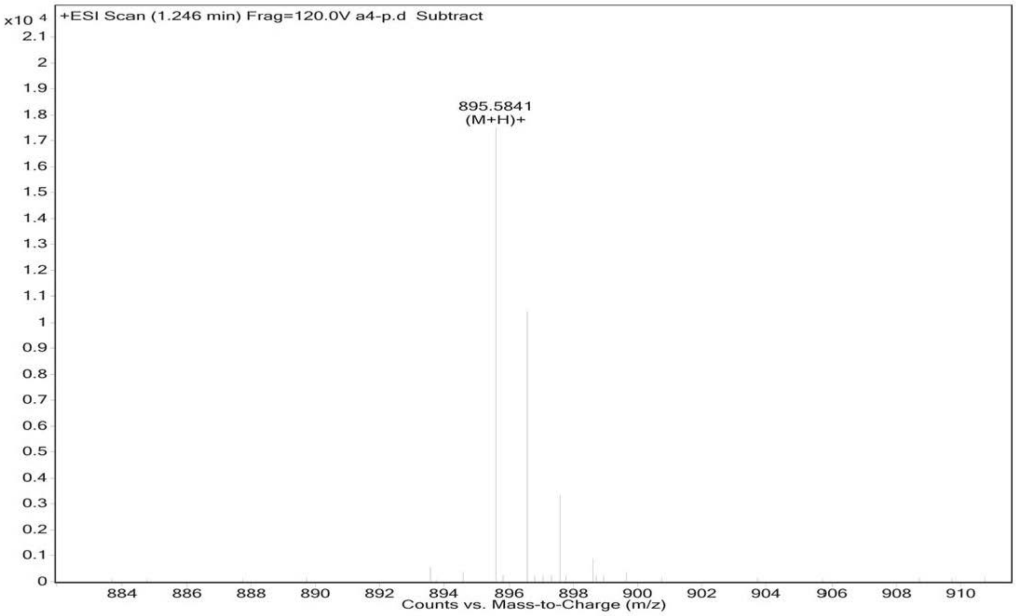

^{1}H NMR spectrum of **22a**

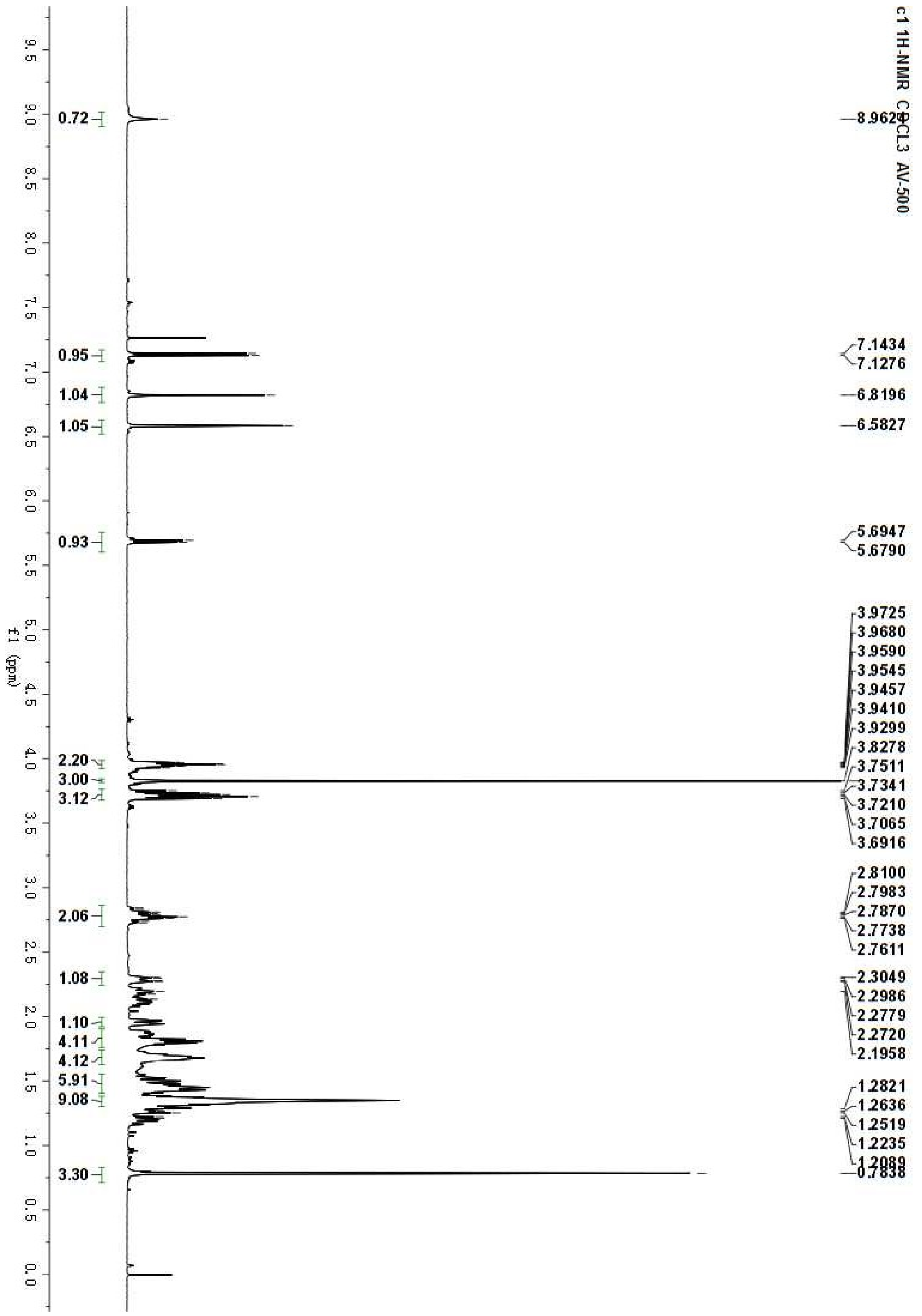

^{13}C NMR spectrum of **22a**

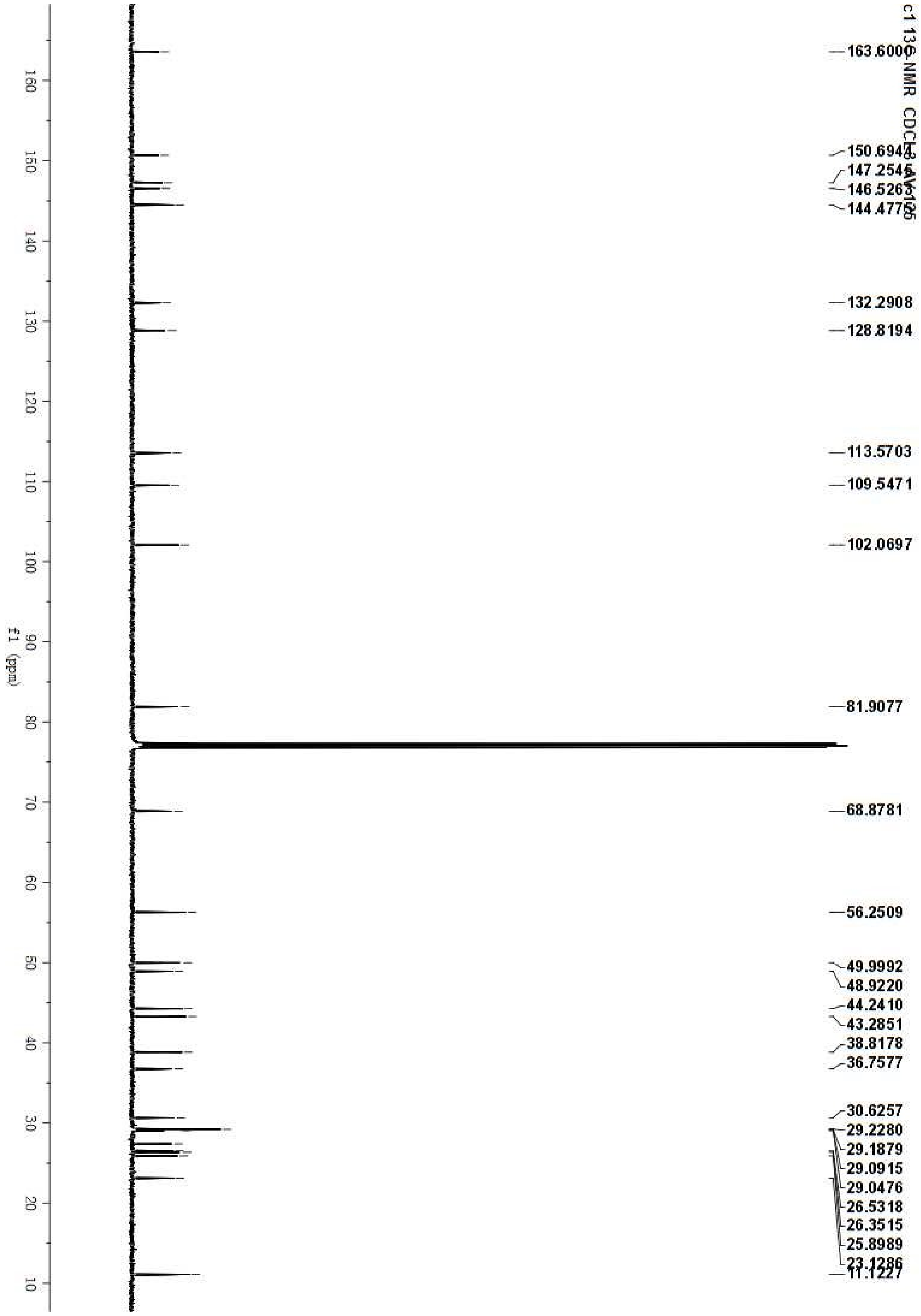

HRMS spectrum of **22a**

**Sample Name**		**Position**	P2-B1	**Instrument Name**	Instrument 1	**User Name**	
**Inj Vol**	0.1	**InjPosition**		**SampleType**	Sample	**IRM Calibration Status**	Success
**Data Filename**	c1-p.d	**ACQ Method**		**Comment**		**Acquired Time**	

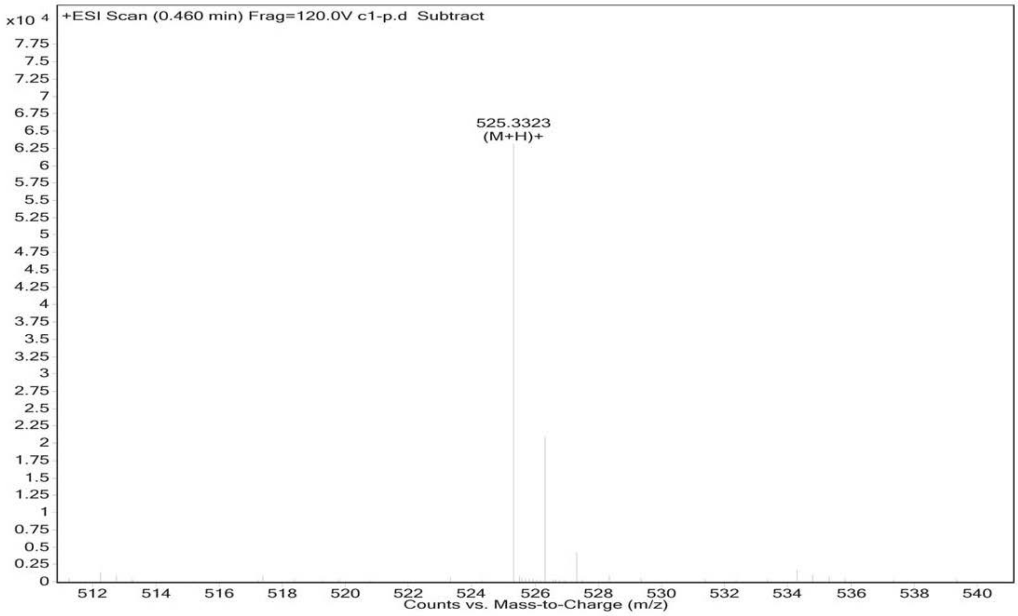

^{1}H NMR spectrum of **22b**

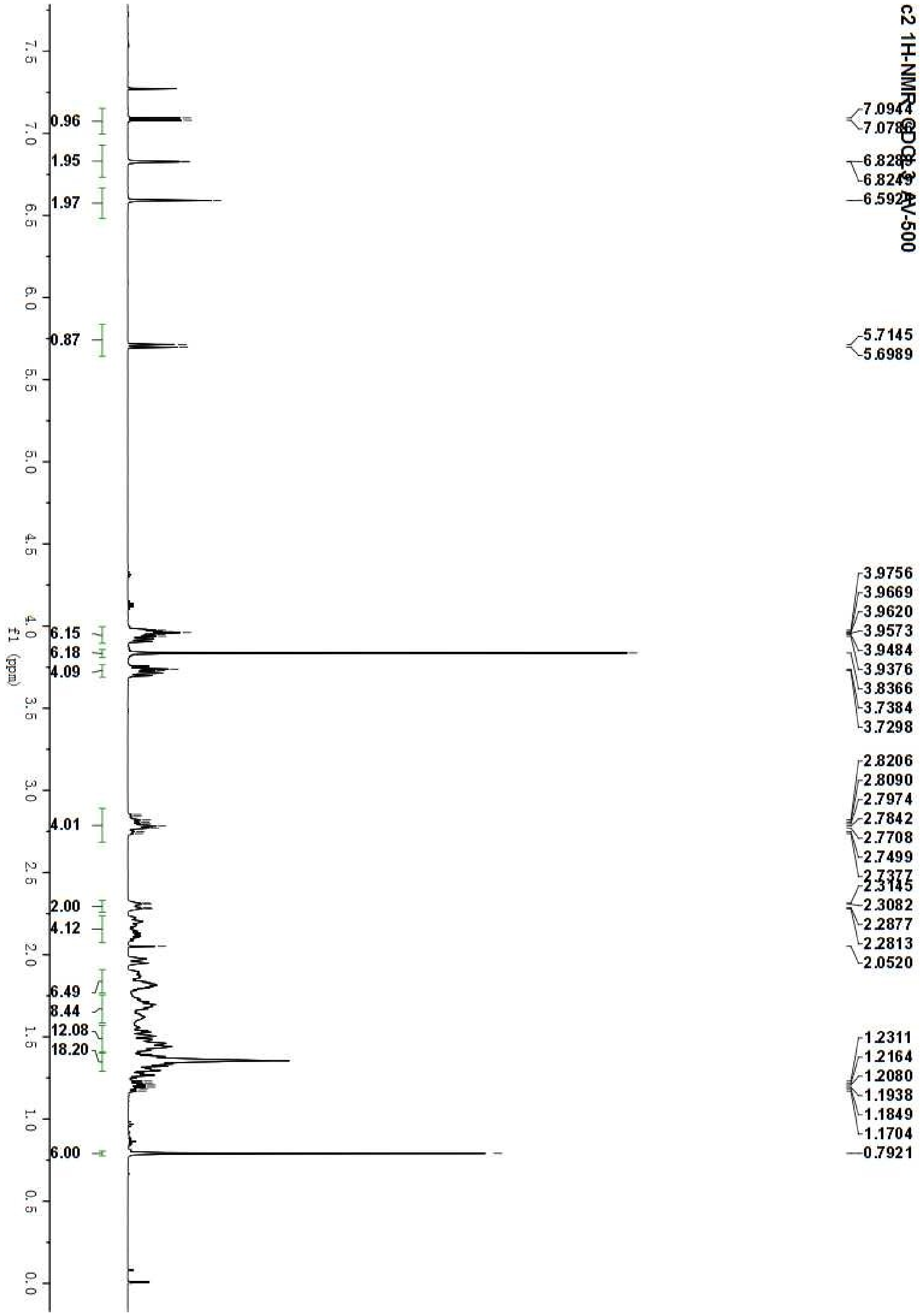

^{13}C NMR spectrum of **22b**

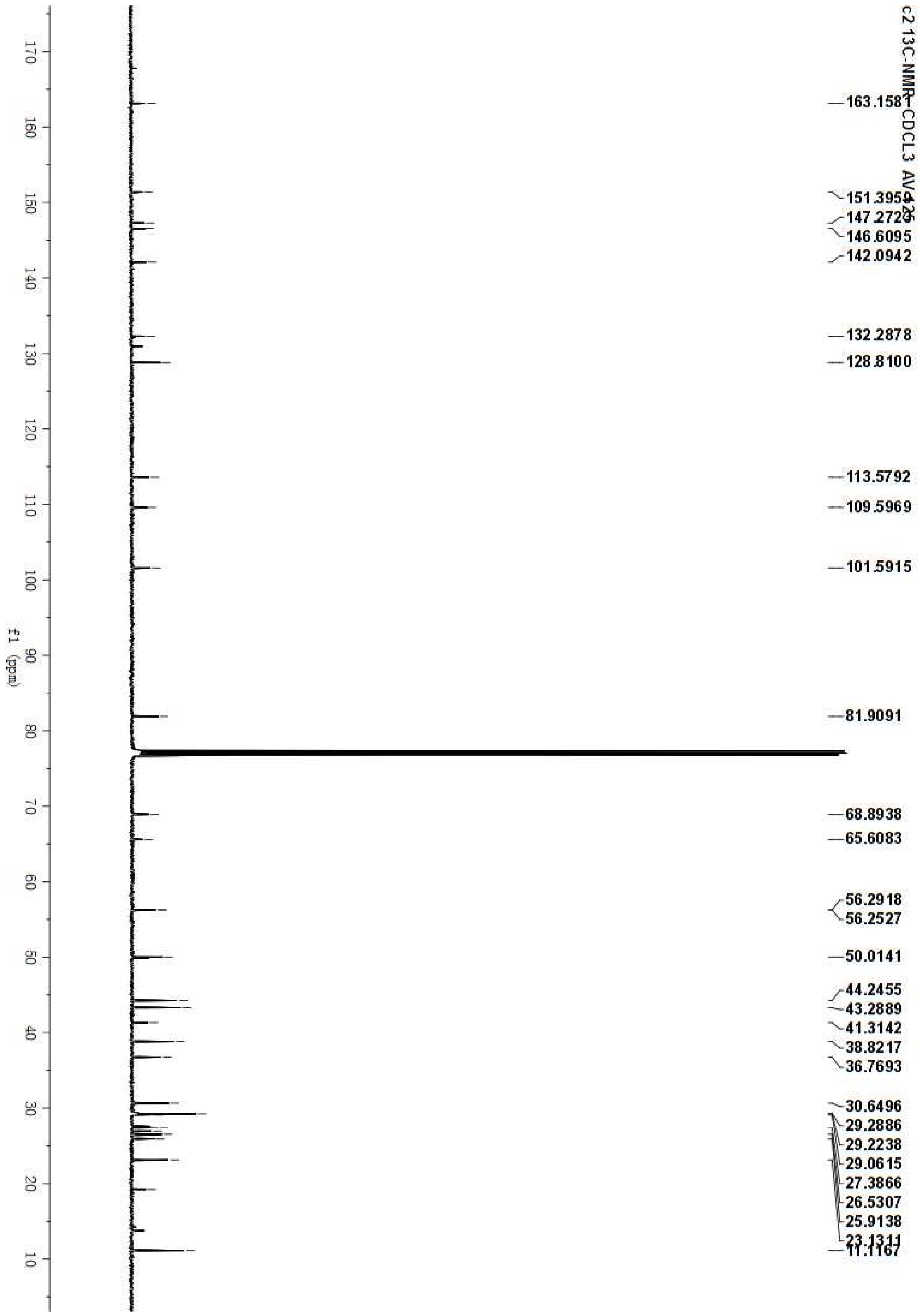

HRMS spectrum of **22b**

**Sample Name**		**Position**	P2-B2	**Instrument Name**	Instrument 1	**User Name**	
**Inj Vol**	0.1	**InjPosition**		**SampleType**	Sample	**IRM Calibration Status**	Success
**Data Filename**	c2-p.d	**ACQ Method**		**Comment**		**Acquired Time**	

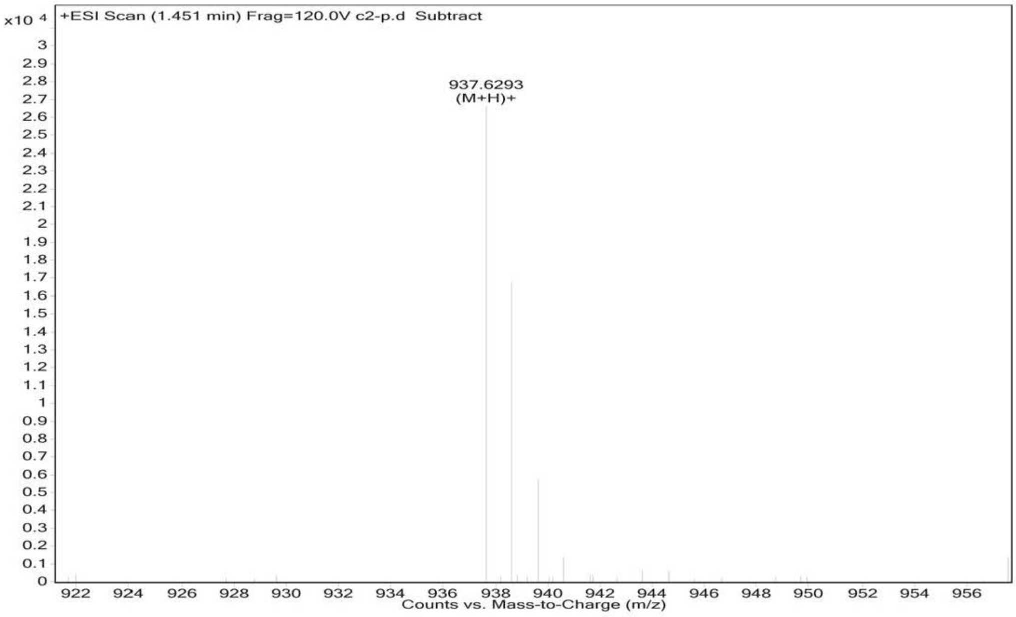

^{1}H NMR spectrum of **23a**

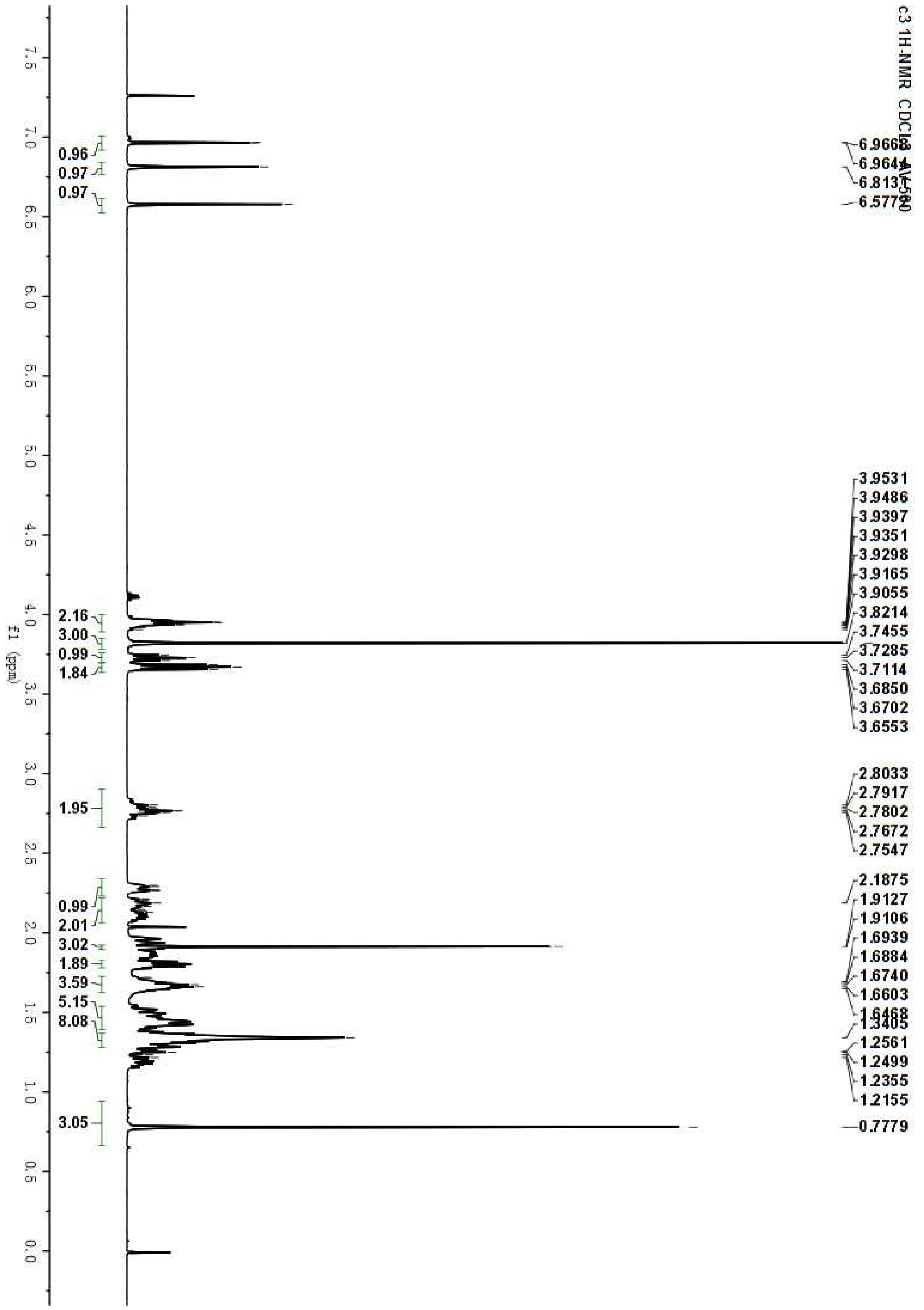

^{13}C NMR spectrum of **23a**

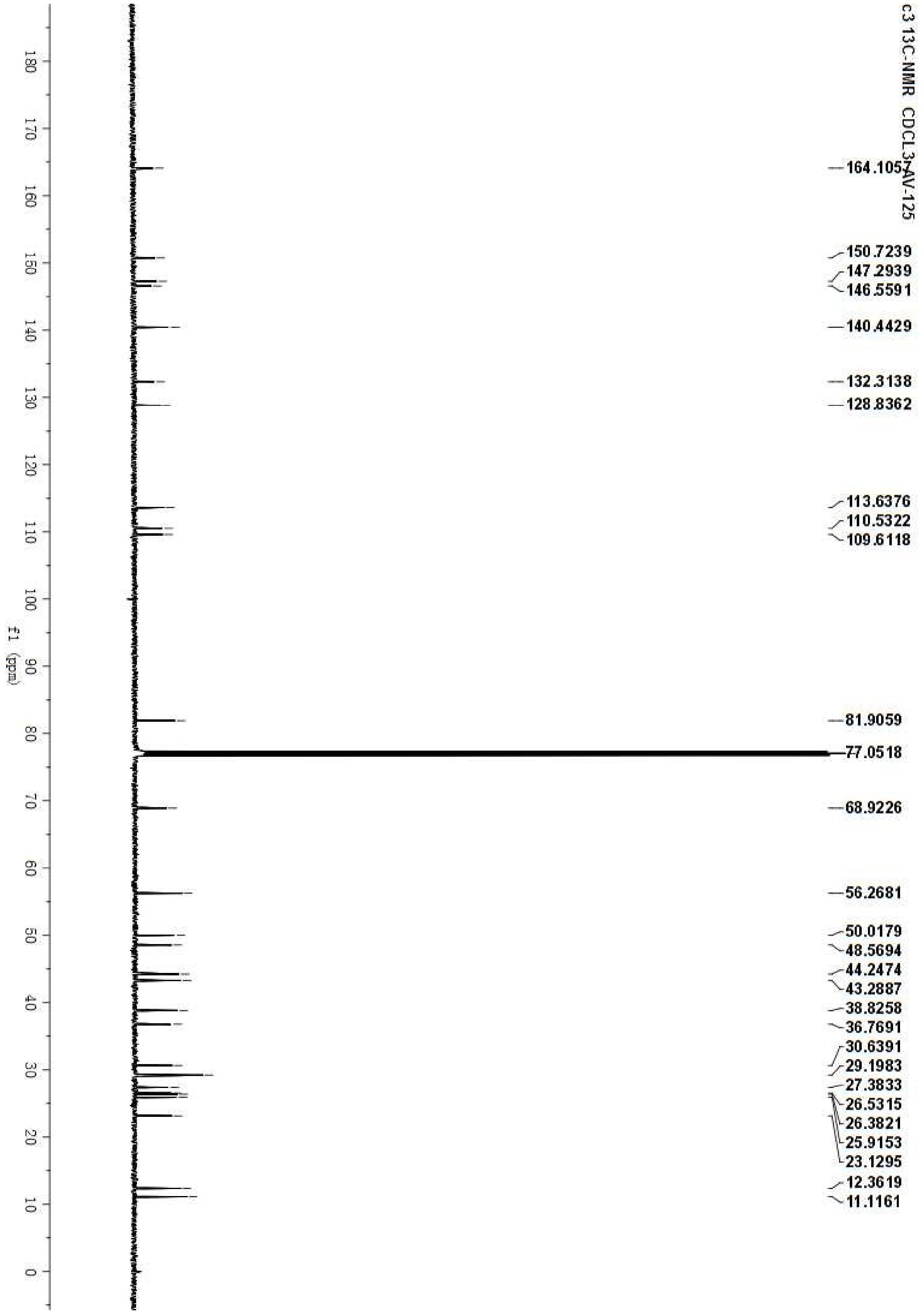

HRMS spectrum of **23a**

**Sample Name**		**Position**	P2-B3	**Instrument Name**	Instrument 1	**User Name**	
**Inj Vol**	0.1	**InjPosition**		**SampleType**	Sample	**IRM Calibration Status**	Success
**Data Filename**	c3-p.d	**ACQ Method**		**Comment**		**Acquired Time**	

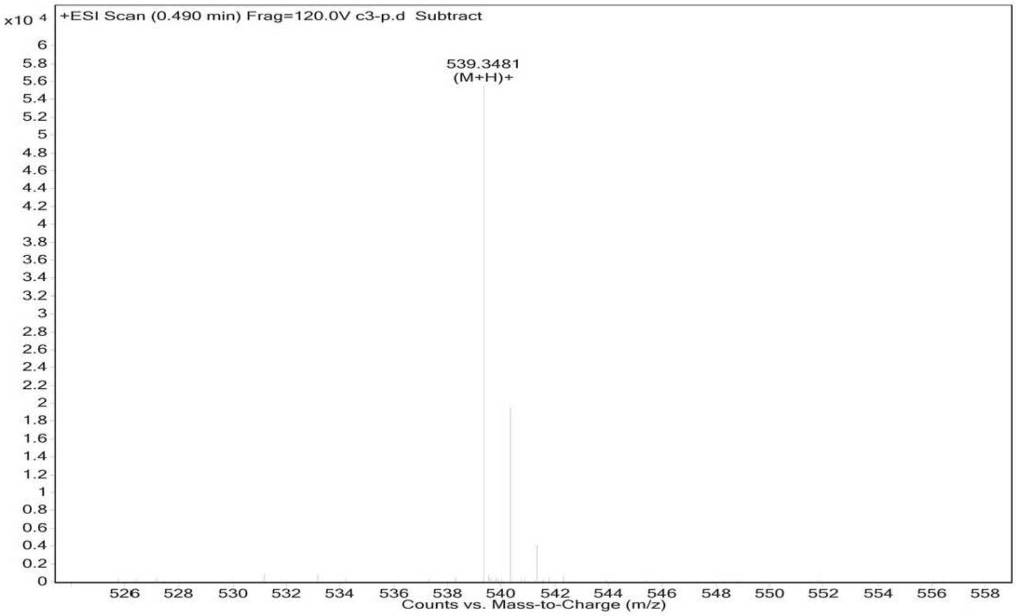

^{1}H NMR spectrum of **23b**

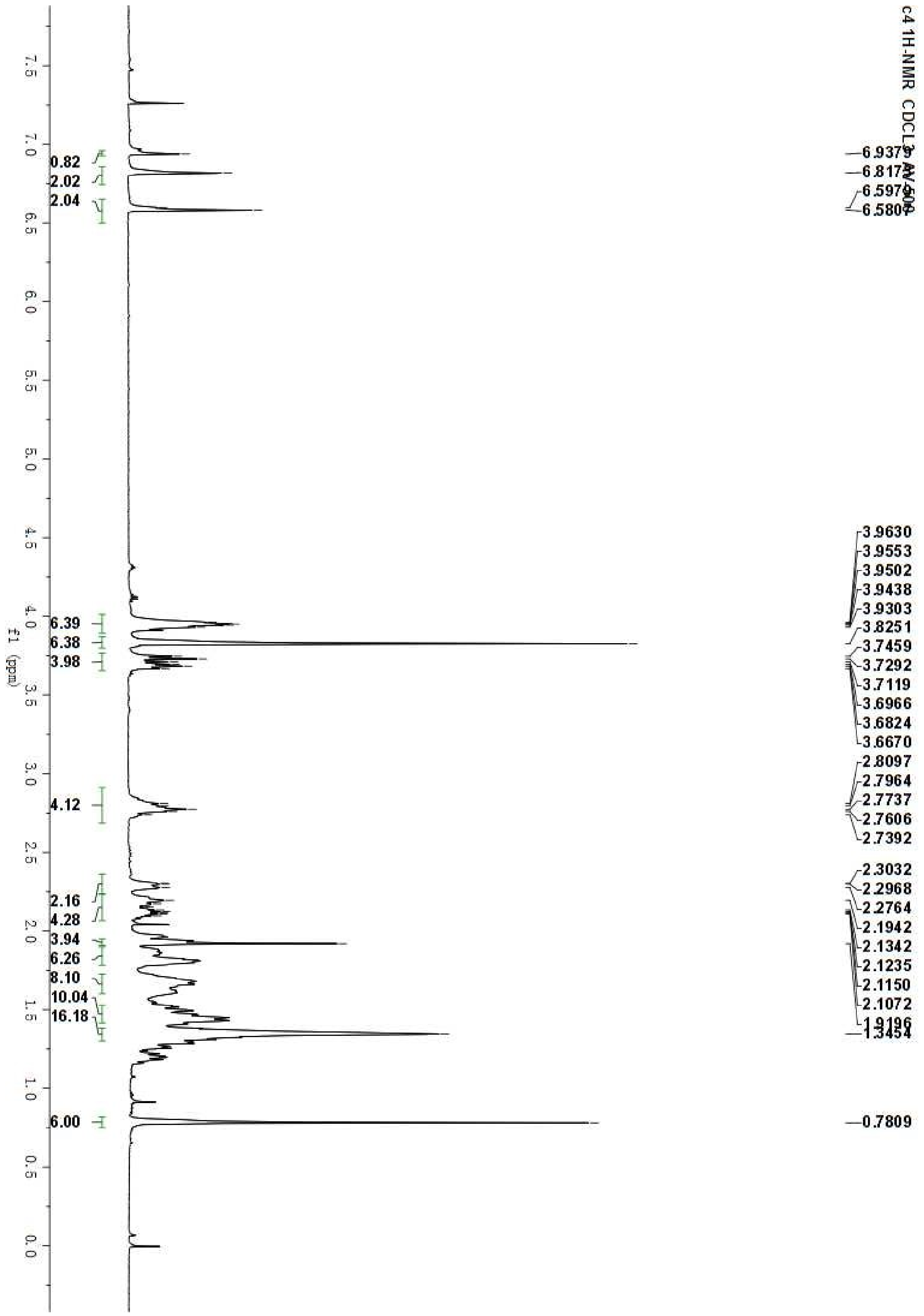

HRMS spectrum of **23b**

**Sample Name**		**Position**	P2-B4	**Instrument Name**	Instrument 1	**User Name**	
**Inj Vol**	0.1	**InjPosition**		**SampleType**	Sample	**IRM Calibration Status**	Success
**Data Filename**	c4-p.d	**ACQ Method**		**Comment**		**Acquired Time**	

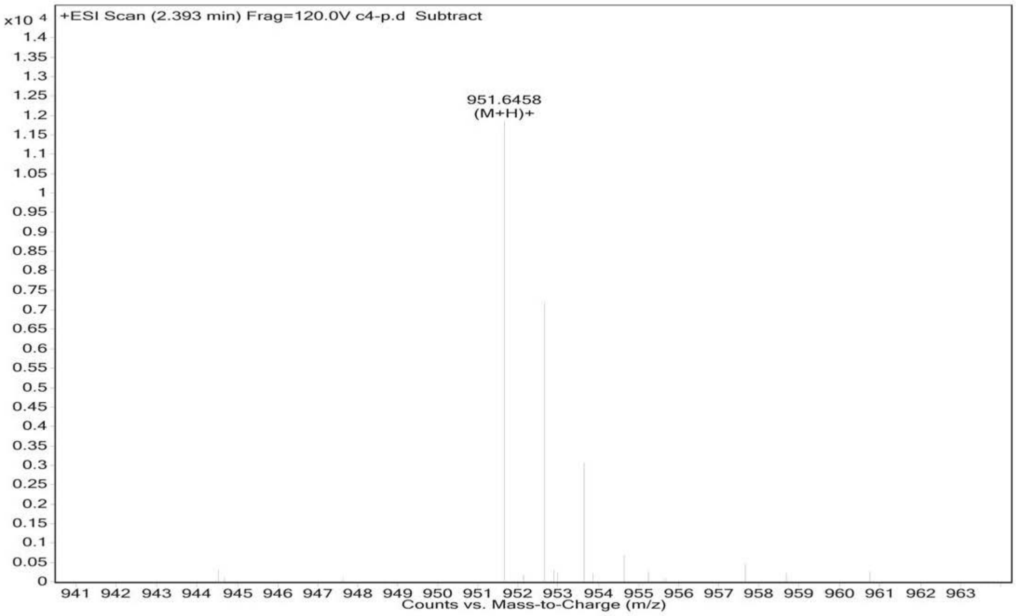

Supplement: Supplementary file 1 [file biomolecules-10-00123-s001.pdf]
